# Role of NAT10-mediated ac4C-modified HSP90AA1 RNA acetylation in ER stress-mediated metastasis and lenvatinib resistance in hepatocellular carcinoma

**DOI:** 10.1038/s41420-023-01355-8

**Published:** 2023-02-10

**Authors:** Zhipeng Pan, Yawei Bao, Mengyao Hu, Yue Zhu, Chaisheng Tan, Lulu Fan, Hanqing Yu, Anqi Wang, Jie Cui, Guoping Sun

**Affiliations:** 1 Department of Medical Oncology, the First Affiliated Hospital of Anhui Medical University, Hefei, Anhui 230022 China; 2 Department of Radiation Oncology, the First Affiliated Hospital of USTC, Division of Life Sciences and Medicine, University of Science and Technology of China, Hefei, China; 3 Department of Integrated Traditional Chinese and Western Medicine, the First Affiliated Hospital of Anhui Medical University, Hefei, Anhui 230022 China

**Keywords:** Cancer metabolism, Acetylation

## Abstract

Emerging evidence showed that epigenetic regulation plays important role in the pathogenesis of HCC. *N*4-acetocytidine (ac4C) was an acetylation chemical modification of mRNA, and NAT10 is reported to regulate ac4C modification and enhance endoplasmic reticulum stress (ERS) in tumor metastasis. Here, we report a novel mechanism by which NAT10-mediated mRNA ac4C-modified HSP90AA1 regulates metastasis and tumor resistance in ERS of HCC. Immunohistochemical, bioinformatics analyses, and in vitro and in vivo experiments, e.g., acRIP-Seq, RNA-Seq, and double luciferase reporter experiment, were employed to investigate the effect of NAT10 on metastasis and drug resistance in HCC. The increased expression of NAT10 was associated with HCC risk and poor prognosis. Cell and animal experiments showed that NAT10 enhanced the metastasis ability and apoptosis resistance of HCC cells in ERS and ERS state. NAT10 could upregulate the modification level of HSP90AA1 mRNA ac4C, maintain the stability of HSP90AA1, and upregulate the expression of HSP90AA1, which further promotes the metastasis of ERS hepatoma cells and the resistance to apoptosis of Lenvatinib. This study proposes a novel mechanism by which NAT10-mediated mRNA ac4C modification regulates tumor metastasis. In addition, we demonstrated the regulatory effect of NAT10-HSP90AA1 on metastasis and drug resistance of ERS in HCC cells.

## Introduction

Hepatocellular carcinoma (HCC) is the most common digestive system malignant tumor in the world, and the sixth-largest malignant tumor in China [1]. It is estimated 906,000 new cases and 830,000 deaths occurred worldwide in 2020 [1]. Radical surgery is the best treatment, but HCC is mostly diagnosed in the advanced stage, with limited treatment options and distant metastasis. Although the development of novel targeted therapies, such as lenvatinib, has improved the survival rate of HCC patients, metastasis and acquired drug resistance still lead to recurrence and poor prognosis. Therefore, it is urgent to explore the causes of HCC metastasis and drug resistance. More and more studies have shown that epigenetic regulation may play an important role in HCC. Understanding the molecular mechanisms of HCC metastasis and tumor drug resistance may provide new therapeutic targets for advanced HCC.

Recent studies indicated that mRNA modification plays a key role in transcriptional regulation, stability, subcellular localization, and translation of mRNA [2–5]. There are various chemical modifications of mRNA, including m6A [6], m5C [7], N4-Achylcytidine (ac4C) [8], m7G [9], m1A [10], etc. Previous studies were mostly focused on the mRNA modifications as m6A and m5C modifications, but study about the ac4C modification was still scarce [11–14]. ac4C is a conserved chemical modification in eukaryotic prokaryotes [4]. Early studies suggested that ac4C mainly exists on tRNA and 18 S rRNA, but recent studies found that there is a large amount of ac4C medication on mRNA, which plays an important role in promoting protein translation, affecting RNA stability and alternative splicing, and regulating gene expression stability [4]. ac4C is also reported in the pathogenesis of a series of human diseases, such as osteoporosis [15], bladder cancer [16], and gastric cancer [17]. However, there are few studies on the role and mechanism of NAT10-mediated mRNA ac4C modification in the above studies.

Endoplasmic reticulum stress (ERS) is a suborganelle pathological state caused by a variety of physical and chemical factors inside and outside the cell, mainly manifested as calcium homeostasis imbalance and excessive protein synthesis. HCC cells have abundant endoplasmic reticulum in their lumen, which is prone to ERS under conditions such as inflammation, ischemia, hypoxia, and oxidative stress [18]. Studies also found that ERS is considered to have tumorigenic and immunosuppressive effects in cancer treatment [19]. In addition, ERS is closely associated with drug resistance in multiple tumors, such as multiple myeloma, breast cancer, kidney cancer, and gastric cancer [20–23].

NAT10 is the only known nuclear protein that has been extensively studied to act as a lysine acetyltransferase. Previous studies also indicated NAT10 may participate in the modifier of RNA ac4C [4]. NAT10 has been reported to delay aging [24], prevent osteoporosis [15] and promote tumor metastasis [25]. In 2021, Zhang et al. found that NAT10 could promote the metastasis of gastric cancer through ac4C acetylation of COL5A1 [17], and Wei Rongfang et al. found that NAT10 could regulate cell proliferation and cell cycle distribution through acetylation of CEP170mRNA in multiple myeloma and improve translation efficiency [26]. In HCC, loss of nucleolar localization of NAT10 promotes cell migration and invasion [27], and the up-regulation of NAT10 can promote the metastasis of HCC cells from epithelial cells to mesenchymal cells [28], enhance the doxorubicin resistance of human HCC cell lines [29], and also promote the metastasis of bladder cancer and laryngeal cancer cells. Besides, NAT10 was reported to could inhibit cell apoptosis in acute myeloid leukemia by strengthening ERS [30]. Additionally, some studies also found that ERS is considered to have tumorigenic and immunosuppressive effects in cancer [19], and NAT10 can enhance ERS and inhibit cell apoptosis in acute myeloid leukemia [30]. In breast cancer, NAT10-mediated acetylation of MORC2 regulates cell cycle checkpoint control and resistance to DNA-damaging chemotherapy and radiotherapy [31].

The HSP90AA1 gene is a subtype of HSP90α. Previous studies have shown that HSP90AA1 signaling is closely related to drug resistance of osteosarcoma [32] and distant metastasis of breast cancer cells [33]. In addition, studies have found that the interaction between HSP90AA1 and phospholipids can stabilize cell membranes under stress conditions [34]. Changes in the extracellular matrix play a key role in tumor angiogenesis and tumor metastasis, and can promote tumor metastasis. Wang Long et al. [35] suggested that HSP90AA1 promoted the progression of lung squamous cell carcinoma. HSP90AA1 is an oncogenic factor that can drive the carcinogenesis and metastasis of esophageal squamous cell carcinoma [36]. In our pilot study, we found that the expression of NAT10 was associated with risk and prognosis of HCC using data from the TCGA database, and acRIP-Seq assay also indicated that the HSP90AA1 was the potential target of the ac4C modification of NAT10 in HCC. Therefore, we hypothesized that NAT10 could participate in the metastasis and Lenvatinib resistance of HCC by regulating the ac4C modification of HSP90AA1 and affecting the ERS process.

## Result

### NAT10 is highly expressed in HCC

**Fig. 1:**
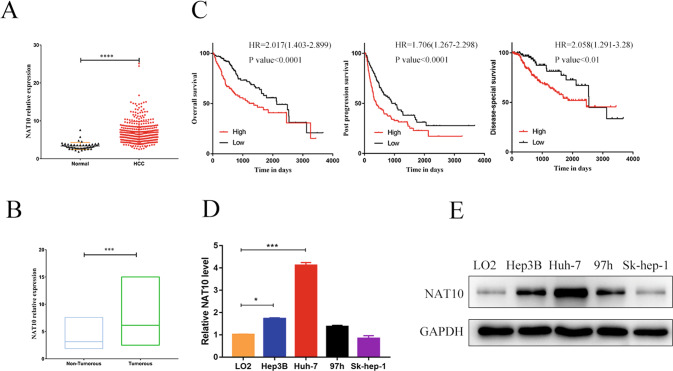
NAT10 was highly expressed in HCC and associated with a worse prognosis. **A** TCGA STAD dataset analysis showed that the expression of NAT10 in HCC patients was significantly higher than that in healthy people (*P* < 0.0001). **B** GEO database (GSE 7186) was used to evaluate the expression of NAT10 in HCC tissues and corresponding adjacent tissues (*n* = 50), and the expression of NAT10 in cancer tissues significantly upregulated *P* < 0.001). **C** Kaplan–Meier analysis showed that the OS, PPS, and DSS of HCC patients with high NAT10 expression were significantly lower than those with low NAT10 expression. **D** qRT-PCR showed that the expression levels of Huh-7 and Hep3B NAT10 in four HCC cell lines were significantly upregulated compared with those in normal hepatocytes LO2. **E** Protein analysis of NTA10 in LO2 and different HCC cell lines by Western blot showed significant expression of NAT10 protein in Hep3b and Huh-7. Each experiment was performed in triplicate, and data are shown as mean ± standard deviation. *P < 0.05, **P < 0.01, ***P < 0.001.

Analysis of RNA-Seq data in the TCGA STAD database showed that NAT10 expression was significantly upregulated in HCC patients compared with healthy people (*P* < 0.0001) (Fig. 1A). The expression of NAT10 in 50 HCC tissues and corresponding adjacent tissues was evaluated using GEO database (GSE 7186), and the expression of NAT10 in cancer tissues was significantly upregulated (*P* < 0.0001, Fig. 1B).

Kaplan–Meier survival analysis was used to analyze the relationship between NAT10 expression and the clinical prognosis of HCC patients using data from the TCGA STAD database. The results suggested that HCC patients with high NAT10 expression had significantly lower OS (overall survival), PPS (post-progression survival), and DSS (disease-specific survival) than HCC patients with low NAT10 expression (Fig. 1C).

Immunohistochemistry was used to evaluate the expression of NAT10 in 100 HCC tissues and corresponding adjacent tissues. There were 57 cases with high expression of NAT10, 35 cases with low expression, and eight cases with negative expression of NAT10. The expression of NAT10 in patients with HCC complicated with hepatitis B and cirrhosis was significantly higher than that in patients with HCC alone (*Z* = −2.433, *P* = 0.015), but not significantly associated with other factors (Table 1).

**Table 1:** Relationship between NAT10 expression level and clinical characteristics of HCC patients.

Clinical features	Number	NAT10			Positive rate (%)	Z/χ^2^	*P*
		Negative	Low	High			
Sex						0.265	0.791
Male	80	6	28	46	92.5		
Female	20	2	7	11	90.0		
Age						0.542	0.588
≤60 years	48	2	18	28	95.8		
>60 years	52	6	17	29	88.5		
History of hepatitis						7.217	0.027*
Positive	76	3	27	46	96.0		
Negative	24	5	8	11	79.0		
History of cirrhosis						8.371	0.015*
Positive	78	3	29	46	96.2		
Negative	22	5	6	11	77.3		
Tumor size						1.139	0.255
≤5 cm	59	5	23	31	91.5		
>5 cm	40	3	11	26	92.5		
Differentiation						2.166	0.339
Low	30	2	13	15	93.3		
Middle	64	6	21	37	90.6		
High	6	0	1	5	100.0		
Single/Multiple tumor						1.416	0.157
Single	83	8	30	45	90.4		
Multiple	17	0	5	12	100.0		
AFP						0.987	0.324
Normal	43	6	14	23	86.0		
Abnormal	57	2	21	34	96.5		
ALT						1.296	0.195
Normal	35	2	10	23	94.3		
Abnormal	65	6	25	34	90.8		
AST						0.304	0.761
Normal	40	4	12	24	90.0		
Abnormal	60	4	23	33	93.3		

*NAT10* *N*-acetyltransferase 10, *AFP* alpha-fetoprotein, *ALT* alanine aminotransferase, *AST* aspartate aminotransferase.

*p value <0.05.

Western blot and qPCR in normal hepatocytes LO2 and four HCC cell lines (Huh-7, Hep3B, MHCC97h, and SK-HEP-1) also showed that protein and RNA levels of NAT10 were upregulated in Huh-7 and hep3B HCC cell lines compared with LO2 cell line (Fig. 1D, E). Therefore, Huh-7 and Hep3B cells were used as experimental subjects in subsequent experiments.

These results indicate that the expression of NAT10 is significantly upregulated in HCC tissues and human HCC cell lines, which may be related to the occurrence, development, and prognosis of HCC.

### NAT10 promotes ERS in HCC cells

**Fig. 2:**
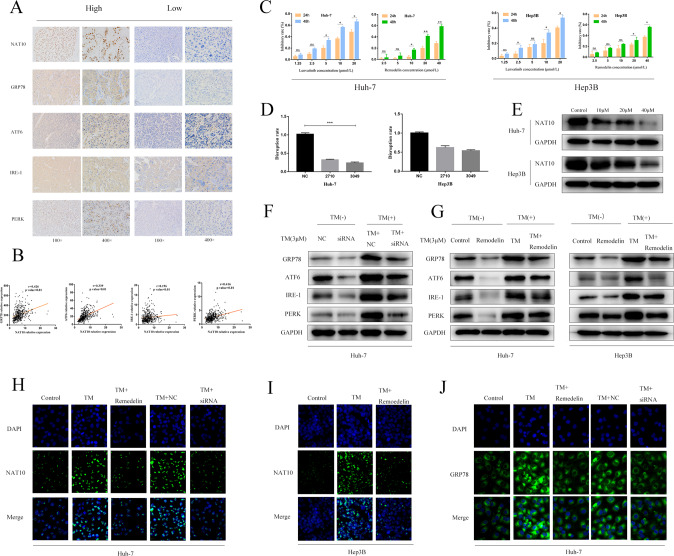
NAT10 promotes ERS in HCC cells. **A** Immunohistochemistry (IHC) was used to analyze the clinicopathologically collected HCC tissues (*n* = 100), and the results showed that the expression of four ERS marker proteins was positively correlated with NAT10. **B** Using the TCGA database, it was found that the correlation analysis between NAT10 and ERS marker proteins in HCC tissues was positive. **C** CCK-8 method was used to detect the inhibition rate of Lenvatinib and Remodelin on the two cell lines. **D** qPCR was used to detect the knockdown effect of siRNA on the expression of NAT10 in the two cell lines, and the results showed that Huh-7 alone could knock down the expression of NAT10 by more than 70%. **E** Western blot showed that Remodelin, a NAT10 inhibitor, significantly inhibited the expression of NAT10 in Huh-7 and hep3B cell lines. **F** huh-7 cells were treated with NAT10-siRNA for 24 h. Total cell extracts were prepared and analyzed by western blotting using antibodies against GRP78, ATF6, IRE-1, PERK, and GAPDH. **G** huh-7 and Hep3B cells were treated with Remodelin (20 μmmol/L) for 24 h. Total cell extracts were prepared and analyzed by western blotting with antibodies to GRP78, atf-6, ire-1, PERK, and GAPDH. **H** The percentage of NAT10(green) in HuH-7 cells increased 24 h after TM (3 μmmol/L) induction, and decreased 24 h after siRNA and Remodelin treatment. **I** The percentage of NAT10(green) in hep3B cells increased 24 h after TM (3 μmmol/L) induction, and decreased 24 h after siRNA and Remodelin treatment. **J** After 24 h of TM (3 μmmol/L) induction, the percentage of GRP78(green) in Huh-7 cells increased, and after 24 h of TM induction, the percentage of GRP78(green) in HuH-7 cells decreased after 24 h of siRNA and Remodelin treatment. Each experiment was conducted in triplicate. Data were shown as mean ± standard deviation. *P < 0.05, **P < 0.01, ***P < 0.001.

Immunohistochemistry in 100 HCC tissues showed that NAT10 was positively correlated with ERS marker proteins (GRP78, ATF-6, IRE-1, and PERK; Supplementary Table S3 and Fig. 2A). Using the TCGA database, the mRNA level of NAT10 was positively correlated with the mRNA level of ERS markers (GRP78, ATF6, IRE-1, and PERK) in HCC tissues was positive (Fig. 2B).

In order to investigate the effect of NAT10 on ERS status of liver cancer cells, siRNA-NAT10-1 (2710), siRNA-NAT10-2(3049), and SI-NC were established. The corresponding stable cell lines were obtained by Lentivirus infection. The expression of NAT10 was confirmed by Western blot and qPCR. The results showed that the expression of NAT10 were significantly decreased after the transfection of siRNA-NAT10-2(3049) and siRNA-NAT10-1(2710) plasmids in the Huh-7 cell line, with knockout efficiency larger than 70%, but not hep3B cell line (Fig. 2D). Therefore, only Huh-7 cell line was used as the experimental object in subsequent siRNA transfection experiments. Considering the effect of si-NAT10-2(3049) was better than siRNA-NAT10-1(2710), the siRNA-NAT10-2(3049) plasmid was selected for subsequent experiments. Remodelin is a special NAT10 inhibitor, could significantly inhibit the expression of NAT10 in both cell lines (Fig. 2E). Therefore, only the Huh-7 cell line was used as the experimental object in subsequent siRNA transfection experiments.

The results showed that the protein and RNA expression levels of NAT10 were positively associated with ERS intensity. Knockdown of NAT10 and NAT10-siRNA inhibitors could decrease the protein expression levels of GRP78, ATF-6, IRE-1, and PERK(Fig. 2E, G). The fluorescence intensity of GRP78 after NAT10 inhibition was lower than that before inhibition. All these results showed that NAT10 was positively correlated with the expression of ERS (Fig. 2H–J). Therefore, we conclude that NAT10 plays a role in promoting ERS in HCC cells. These results suggest that NAT10 may promote the occurrence and development of ERS in HCC cells.

### NAT10 enhances the migration, invasion, and cell cycle arrest of HCC cells in ERS state

**Fig. 3:**
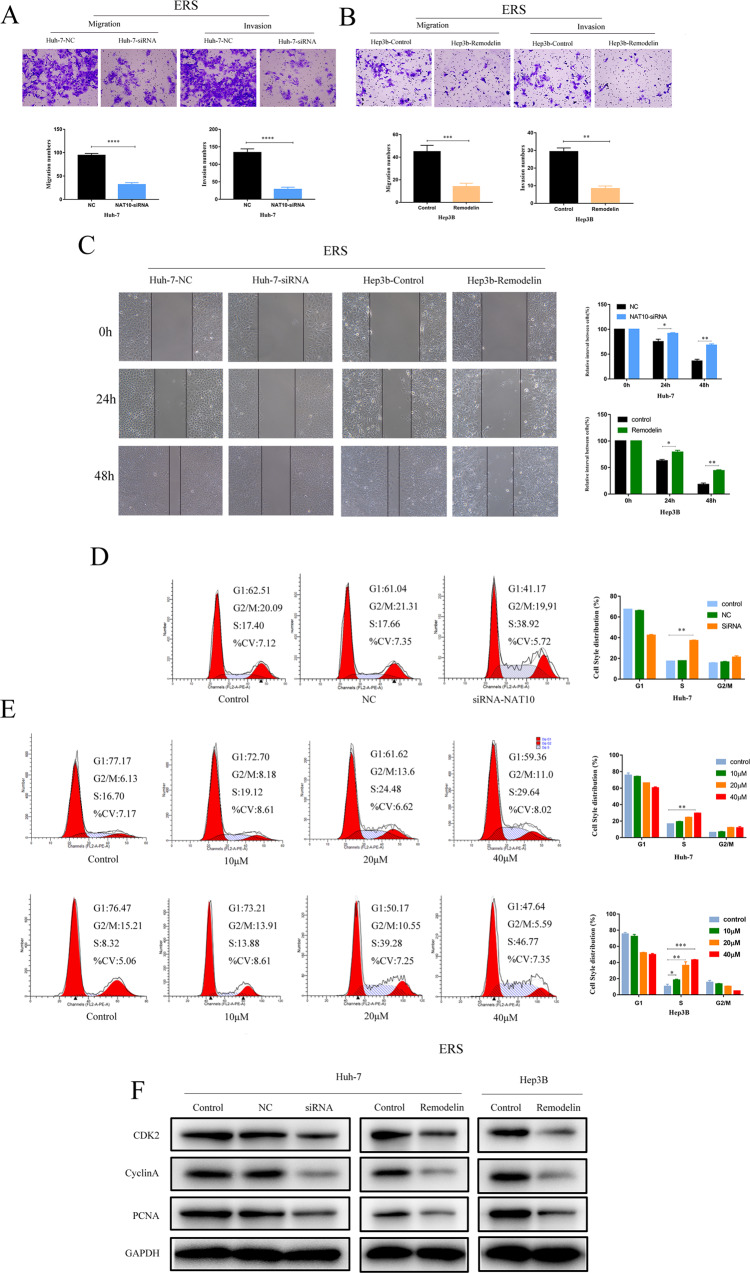
NAT10 enhances the migration, invasion, and cell cycle arrest of hepatocellular carcinoma cells in ERS state. After 24 h of TM (3 μmmol/L) induction, after siRNA-NAT10 or Remodelin treatment, the migration and invasion of Huh-7 (**A**) and Hep3B cells (**B**) were analyzed by transwell, scale = 200 μm. **C** Similarly, the migration of Huh-7 and Hep3B cells was analyzed by wound healing assay (scale = 100 μm). The effect of siRNA-NAT10 (**D**) and its inhibitor Remodelin (**E**) on cell cycle arrest (*n* = 3) was analyzed by flow cytometry. Huh-7 and hep3B cells were treated with different concentrations of Remodelin for 48 h to conduct cell cycle arrest assay. **F** Western blot showed the protein levels of CDK2, CyclinA, PCNA, and other related cell cycle genes in Huh-7 and hep3B cells after NAT10 knockdown or inhibition. All cells were induced by TM 24 h in advance, and each experiment was conducted in three times. Data were shown as mean ± standard deviation. *P < 0.05, **P < 0.01, ***P < 0.001.

In the HCC model in vitro, transwell assay showed that knockdown of NAT10 could significantly inhibit the migration and invasion ability of ERS HCC cells (Fig. 3A, B), and cell scratch assay showed that silencing of NAT10 could significantly inhibit the migration ability of Huh-7 and Hep3b cells in ERS state (Fig. 3C). Cell cycle analysis showed that NAT10-siRNA treatment significantly increased the percentage of S-phase cells in HuH-7 cell lines compared with untreated control and NC groups. Cell cycle analysis showed that the proportion of S-phase in Huh-7 and hep3B cells was significantly increased after the transfection of Remodelin in a dose-dependent manner (Fig. 3D, E). After knockdown and inhibition of NAT10, the expressions of CDK2, CyclinA, and PCNA were decreased in WB (Fig. 3F). In the in vitro model experiment, the tumor growth of nude mice injected with Remodelin was significantly slower than that of the control group(Fig. 6F, G). These results indicate that NAT10 can promote the migration, invasion, and S-phase cell cycle arrest of ERS HCC cells in vitro.

### NAT10 promoted the anti-apoptotic ability and Lenvatinib resistance of ERS hepatoma cells

**Fig. 4:**
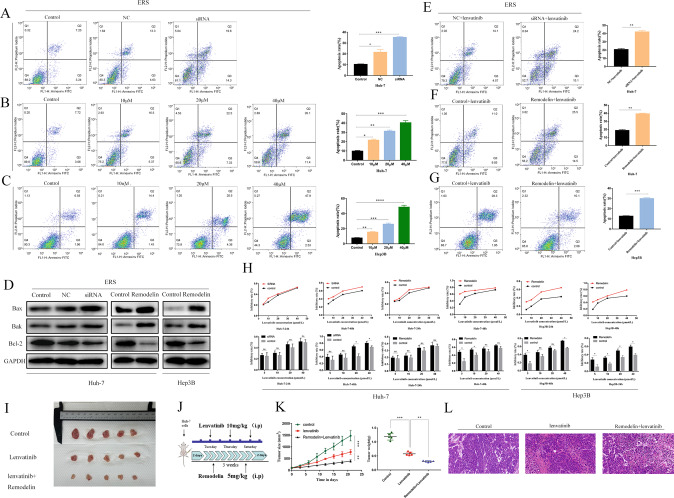
NAT10 promoted the anti-apoptotic ability and Lenvatinib resistance of ERS hepatoma cells. **A**–**C** The effects of siRNA-NAT10 and its inhibitor Remodelin (**B**, **C**) on the apoptosis of Huh-7 cells and Hep3B cells after NAT10 knockdown and inhibition were analyzed by flow cytometry (*n* = 3). **D** Western blot showed the expression levels of Bax, Bak apoptotic protein, and Bcl-2 anti-apoptotic protein genes in Huh-7 and hep3B cells after NAT10 knockdown or inhibition. The effect of siRNA-NAT10 (**E**) and its inhibitor Remodelin (**F**, **G**) on the sensitivity of Huh-7 cells and Hep3B cells to Lenvatinib after NAT10 knockdown and inhibition was analyzed by flow cytometry (*n* = 3). **H** CCK-8 assay was used to study the effects of NAT10 knockdown or inhibition on the viability of Huh-7 and Hep3B cells treated with different concentrations of Lenvatinib for 24 and 48 h. **I** Photographic images of xenograft tumors collected (*n* = 5). **J** Schematic diagram of mouse xenograft tumor administration. **K** Average tumor volume and weight of xenograft mice in the groups on day 21. **L** HE staining of mouse metastatic tumors. All groups of cells were subjected to TM induction 24 h in advance, and each experiment was conducted three times. Data were shown as mean ± standard deviation. *P < 0.05, **P < 0.01, ***P < 0.001.

In Huh-7 HCC cells, the apoptosis rate of the NAT10-siRNA knockout group was significantly higher than that of untreated control and NC (Fig. 4A). Similar results were observed after Remodelin treatment (Fig. 4B, C). Remodelin treatment induced a dose-dependent increase in the apoptosis rate of Huh-7 and hep3B cells. WB also showed that the expressions of apoptotic proteins Bak and Bax were decreased and the expression of anti-apoptotic protein Bcl-2 was decreased after NAT10 inhibition (Fig. 4D).

We also verified the effect of NAT10 on the sensitivity and apoptosis of Huh-7 and Hep3B cell lines to Lenvatinib. After the treatment of siRNA and Remedelin in Huh-7 and Hep3B for 24 and 48 h, flow cytometry showed that Lenvatinib could significantly increase the apoptosis rate of Huh-7 and Hep3B (Fig. 4E–G).

CCK-8 experiment showed that Huh-7 and Hep3B cells treated with NAT10-siRNA and Remodelin had the effect of sensitivity to Lenvatinib within 24 and 48 h. The results showed that only Hep3B cells showed decreased sensitivity to Lenvatinib within 24 h. There was no significant difference in the sensitivity of Huh-7 cells to Lenvatinib. Within 48 h, only Huh-7 cells treated with siRNA showed decreased sensitivity to Lenvatinib, and hep3B showed decreased sensitivity to Lenvatinib (Fig. 4H).

In the in vitro model experiment, the growth of xenograft tumors in nude mice treated with Lenvatinib was significantly slower than that in the control group, and the growth of xenograft tumors in nude mice treated with Remodelin was slower than that in the Lenvatinib group (Fig.4I–L).

### HSP90AA1 is a downstream regulatory target of NAT10

**Fig. 5:**
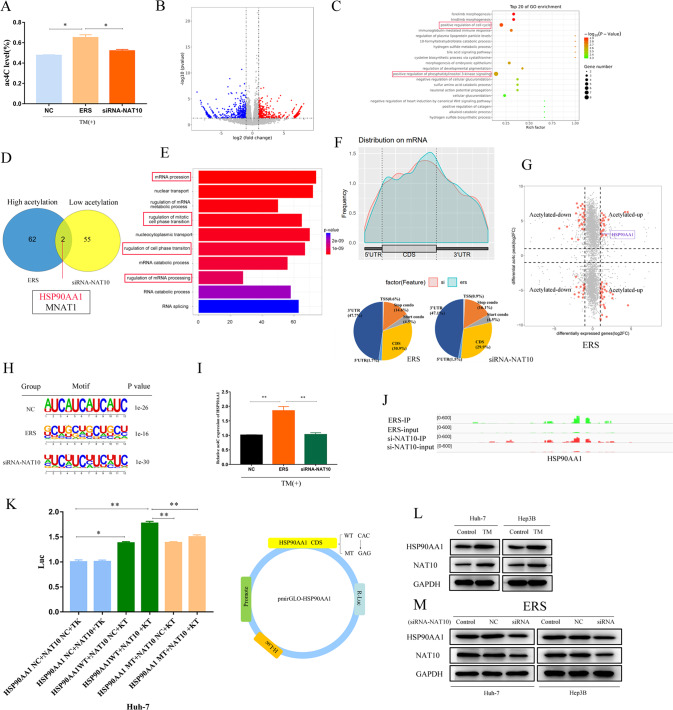
HSP90AA1 is a downstream regulatory target of NAT10. Huh-7 cells induced by TM and knocked down by NAT10 were subjected to liquid chromatography–tandem mass spectrometry, ACRIP-Seq, and RNA-Seq. **A** Compared with the control group, the total amount of mRNA ac4C modification in Huh-7 cells was significantly increased after ERS transfection and significantly decreased after siRNA knockout (*P* < 0.05). **B** RNA-seq Volcano plots depicting genes with upregulated (red) and downregulated (blue) mRNA levels. **C** RNA-seq GO pathway enrichment analysis revealed that the differential genes were located in the cell cycle and extracellular matrix pathways. **D** Gene overlap was detected by comparing genes upregulated by acetylation in TM-induced HuH-7 cells with genes downregulated by siRNA knockdown. **E** acRIP-Seq RNA sequence data pathway enrichment analysis showed that NAT10 was associated with mRNA modification and cell cycle. **F** ac4C peak mainly appeared in the CDS coding region of Huh-7 cells. **G** The mRNA acetylation level was depicted by the four-quadrant map of ACRIP-Seq analysis. **H** Typical CXX motif pattern of ac4C peak. **I** acRIP-qPCR, **J** HSP90AA1 acetylation peak, **K** double luciferase reporter assay. **L**–**M** HSP90AA1 was positively correlated with NAT10 at the protein level. WB analysis of Huh-7 and Hep3B treated with siRNA-HSP90AA1 compared with control cells transfected with vector. All data were expressed as mean ± standard deviation. *P < 0.05, **P < 0.01, ***P < 0.001.

We used liquid chromatography–tandem mass spectrometry to detect the total amount of ac4C modification before and after NAT10 inhibition in normal HCC cells and HCC cells in ER state (Fig. 5A) and RNA-seq found that after NAT10 knockdown, 2193 differential genes (*p* value <0.05, a fold change >1.5-fold), Volcano map shows genes with significant changes in upregulated (red) and downregulated (blue) mRNA acetylation under NAT10 overexpression (Fig. 5B); GO pathway analysis revealed that differentially expressed genes were mainly enriched in cell cycle and extracellular matrix pathways, indicating that NAT10 gene extensively regulated gene expression(Fig.5C).

Next, we used advanced acRIP-seq technology to compare the enrichment of ac4C gene modification in Huh-7 cells before and after NAT10 gene knockout in ERS state (Fig. 5D).

Compared with Huh-7 cells in unstress status, the overall level of ac4C modification in ERS Huh-7 cells was significantly increased, with genes enriched in ERS and cell cycle pathways. The overall level of ac4C modification in siRNA-NAT10 transfected ERS Huh-7 cells was significantly decreased, with genes enriched in cell cycle pathways and mRNA processing and metabolic regulation pathways (Fig. 5E).

Comprehensive analysis of acRIP-Seq and RNA-Seq data showed that NAT10 silencing downregulated ac4C modifications in the CDS coding region of the HSP90AA1 gene (Fig.5F), Through the intersection analysis of acRIP-seq and RNA-seq before and after the establishment of ERS model and after the interference of NAT10 in ERS model, it was found that the acetylation and expression of HSP90AA1 gene were significantly correlated with the expression of NAT10 gene. The genes with changes in ac modification and genes with changes in RNA expression level were analyzed for an association, and the above four groups were divided according to mRNA acetylation level (Fig. 5G).

**Fig. 6:**
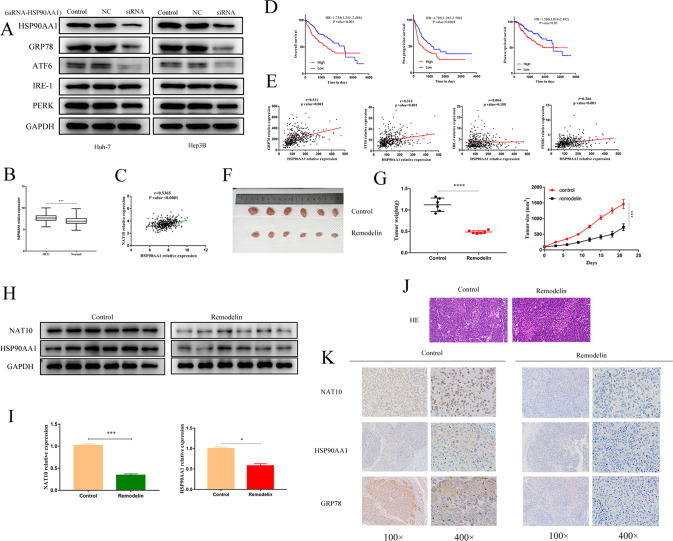
NAT10 can promote the expression of the HSP90AA1 gene in ERS HCC. **A** The effects of Huh-7 and Hep3B cells treated with siRNA-HSP90AA1 on ERS marker genes were analyzed by WB. **B** HSP90AA1 was highly expressed in HCC tissues. **C** There was a positive correlation between HSP90AA1 and NAT10 in HCC tissues. **D** High HSP90AA1 expression was significantly associated with shorter OS, PPS, and DSS. **E** There was a positive correlation between HSP90AA1 and ERS marker proteins GRP78, ATF-6, and PERK in HCC tissues. **F** Photographic images of xenograft tumors collected (*n* = 6). **G** Average tumor volume and weight of xenograft mice in the Control and Remodelin groups on day 21. **H** Western blot showed that the protein levels of NAT10 and HSP90AA1 were decreased in tumor tissues of xenograft mice. **I** qRT-PCR showed that both NAT10 mRNA and HSP90AA1 mRNA decreased in tumor tissues of xenograft mice treated with Remodelin. **J** HE staining of mouse metastatic tumors. **K** IHC staining showed that the protein levels of NAT10, GRP78, and HSP90AA1 in tumor tissues of Remodelin-treated xenografts were decreased. All data were shown as mean ± standard deviation. *P < 0.05, **P < 0.01, ***P < 0.001.

Figure 6H shows the typical CXX motif for the ac4C peak, indicating that the quality of the acRIP-Seq analysis was assured. To further determine the downstream factors of NAT10, we verified the correlation between NAT10 and HSP90AA1 gene expression by acRIP-PCR (Fig. 5I) and found that the expression level of HSP90AAA1 ac4C modification decreased after the knockdown of NAT10 gene expression. In addition, the ac4C peak of HSP90AA1 showed significantly increased enrichment in the ERS IP group compared to si-NAT10 IP (Fig. 5J).

Next, a dual-luciferase reporter gene assay was used to investigate whether NAT10 regulates the expression level of HSP90AA1 through the mRNA ac4C modification pathway. To construct wild-type and mutant HSP90AA1 reporter genes. For the HSP90AA1 mutant, the ac4C consequence CAC was replaced by GAG, thereby abrogating the ac4C modification (Fig. 5K). The luciferase activity of NAT10 and wild-type HSP90AA1 reporter gene was enhanced, and the luciferase activity of NAT10 against mutant HSP90AA1 reporter gene expression was weakened (Fig. 5K). The results showed that NAT10 could bind to HSP90AA1 and promote HSP90AA1 translation. Therefore, NAT10 may modify the CDS coding region of HSP90AA1 through ac4C and positively regulate the expression of HSP90AA1.

Western blot analysis was performed on HCC cell lines Huh-7 and Hep3B in vitro. The expression of HSP90AA1 protein was increased in ERS status (Fig. 5L). Knockdown of NAT10 could reduce HSP90AA1 expression at the protein level (Fig. 5M). In conclusion, HSP90AA1 may be a downstream regulatory target of NAT10-mediated mRNA ac4C modification.

### NAT10 can promote the expression of the HSP90AA1 gene in ERS HCC

WB assay showed that HSP90AA1 silencing downregulated the expressions of GRP78, ATF-6, and PREK, but did not affect the expression of IRE-1 (Fig. 6A), which was consistent with the results of TCGA big data analysis, and the results of NAT10 knockdown.

RNA-Seq data in TCGA STAD dataset also showed that the expression of HSP90AA1 was significantly upregulated in HCC tissues (*P* < 0.001) and was positively correlated with the expression of NAT10 (Fig. 6B, C). Kaplan–Meier survival analysis was used to analyze the correlation between HSP90AA1 expression and the clinical prognosis of HCC patients. The overall survival, post-progression survival, and disease-specific survival of HCC patients with high HSP90AA1 expression were significantly lower than those with low HSP90AA1 expression (Fig. 6D). Meanwhile, RNA-Seq dataset in the TCGA database showed that the expression of HSP90AA1 was significantly positively correlated with the expression of GRP78, ATF-6, and PERK, but no significant correlation with IRE-1 expression in HCC patients (Fig. 6E).

In the HCC model in vivo, IHC, WB, and qPCR were performed using tumor tissues from subcutaneous tumorigenesis experiments in nude mice. Two groups of mice were injected with Remodelin and DMSO respectively, and we found that the expression of GRP78 and HSP90AA1 proteins decreased (Fig. 6H–K).

### HSP90AA1 promotes metastasis, cell cycle arrest, channeling resistance, and Lenvatinib resistance in ERS HCC

**Fig. 7:**
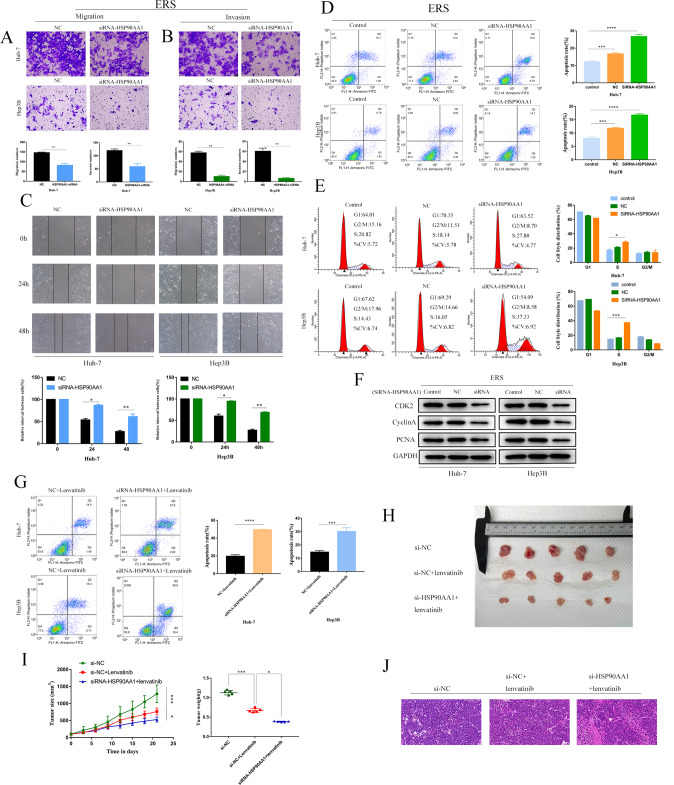
HSP90AA1 promotes metastasis, cell cycle arrest, channeling resistance, and Lenvatinib resistance in ERS hepatocellular carcinoma. After siRNA-NAT10 or Remodelin treatment, Huh-7 (**A**) and Hep3B (**B**) cell migration and invasion were analyzed by transwell, scale = 200 μm. **C** Similarly, the migration of Huh-7 and Hep3B cells was analyzed by wound healing assay (scale = 100 μm). **D** The effect of siRNA-HSP90AA1 knockdown on the apoptosis of Huh-7 and Hep3B cells was analyzed by flow cytometry (*n* = 3). **E** siRNA-HSP90AA1 increased the S-phase fraction of Huh-7 cells by flow cytometry analysis (*n* = 3). **F** Western blot showed the expression levels of CDK2, CyclinA, and PCNA cell cycle proteins in Huh-7 and hep3B cells after HSP90AA1 knockdown or inhibition. **G** Flow cytometry analysis of the effect of siRNA-HSP90AA1 knockdown and inhibition on the sensitivity of Huh-7 cells and Hep3B cells to Lenvatinib (*n* = 3). **H** Photographic images of xenograft tumors collected (*n* = 5). **I** Average weight and volume of the mouse xenograft tumor on day 21. **J** HE staining of mouse metastatic tumors. All groups of cells were induced by TM 24 h in advance. All data were shown as mean ± standard deviation. *P < 0.05, **P < 0.01, ***P < 0.001.

To prove that HSP90AA1 may play a role in promoting metastasis and drug resistance, stable HSP90AA1 knockdown cell lines were established in Huh-7 and Hep3B cells, and the cell scratch assay was performed. Scratch assay showed that HSP90AA1 knockdown significantly inhibited HCC cell migration (Fig. 7C), while transwell assay showed that HSP90AA1 knockdown inhibited HCC cell migration and invasion (Fig. 7A, B). The apoptosis rate of Huh-7 and hep3B cells in the HSP90AA1 knockout group was significantly higher than that in the untreated control group (control) and NC group (Fig. 7D). Cell cycle analysis showed that compared with untreated control and NC groups, Hsp90a1-siRNA treatment significantly increased the percentage of S-phase cells in Huh-7 and hep3B cell lines, and Remodelin treatment induced a dose-dependent increase in the percentage of S-phase in HuH-7 and hep3B cells (Fig. 7E). Quantitative data showed that there were differences between the siRNA-HSP90AA1 group and the control group. Similar results were obtained in the detection of apoptotic proteins Bak, Bax, anti-apoptotic protein Bcl-2, and cyclins CDK2, CyclinA, and PCNA by WB (Fig. 7F). Flow cytometry showed that Lenvatinib increased the apoptosis rate of Huh-7 and Hep3B after HSP90AA1 knockdown (Fig. 7G). In vivo experiments showed that the tumor size of nude mice injected with siRNA-HSP90AA1 was smaller than that of lenvatinib (Fig. 7H–K). Therefore, these results suggest that HSP90AA1 may have similar promoting effects as NAT10 in metastasis, cell cycle arrest, apoptosis resistance, and lenvatinib resistance of ERS HCC.

## Discussion

Chemical modification of eukaryotic prokaryotes is a hot topic in epigenetic transcriptomics, and mRNA modification is one of the most common chemical modifications. With the development of molecular biology and high-throughput sequencing technologies, researchers can accurately quantify and localize mRNA modifications and identify modification sites to regulate a variety of biological functions [37–39]. In recent years, more and more RNA modifications have been discovered. Among them, studies related to mRNA modification mainly focus on m6A and other modifications (such as M5C, ac4C, M7G, M1A, etc.). Little has been done to investigate their potential roles and mechanisms in other diseases [6, 7]. In this study, the expression of NAT10, the only known mRNA ac4C-modified protein, was significantly higher in HCC tissues than in normal liver tissues. In particular, high expression of NAT10 is associated with hepatitis, cirrhosis, and poor prognosis in patients with HCC. These results suggest that NAT10 is an adverse prognostic factor for patients with HCC, and can be used as a biomarker to study the development and progression of HCC, or as a potential target for future HCC diagnosis and drug development.

In this study, we successfully constructed a cell line stably expressing NAT10, tested the effect of NAT10 expression on ERS cells, verified whether NAT10 was involved in the regulation of metastasis and apoptosis resistance of ERS HCC, and tested the effect of NAT10 expression on migration, invasion and Lenvatinib resistance of ERS HCC cells. The results showed that NAT10 was positively correlated with ERS in HCC, and NAT10 could promote the invasion and migration of ERS HCC cells and the resistance to apoptosis of Lenvatinib. Similarly, it has been reported that NAT10 is positively correlated with ERS in acute myeloid leukemia [30] and that high expression of NAT10 induces platinum-based drug resistance in breast cancer [40]. As a member of the GCN5-related N-acetyltransferase superfamily, NAT10 is involved in the regulation of telomerase activity, DNA damage repair, ribosomal RNA synthesis, chromosome regulation, cytokinesis regulation, apoptosis resistance, and cell cycle regulation [41]. It has been considered that NAT10 is an oncogene in solid tumors [42]. In this study, siRNA and Remodelin, a small-molecule inhibitor of NAT10, are also used to inhibit cell proliferation in HCC cells in ERS state, and further, induce S-phase arrest of the cell cycle and apoptosis. The molecular mechanism of NAT10 tumorigenesis in HCC in ERS state was investigated. It was found that inhibition of NAT10 attenuated the UPR pathway of ERS unfolded protein response and further promoted cell apoptosis. However, our study highlights the role of NAT10 in metastasis and tumor resistance in HCC under ERS and reveals the therapeutic potential of NAT10 in HCC. In vivo study, the subcutaneous tumor-forming assay was used in nude mice to detect the changes in the subcutaneous tumor-forming ability of HCC cells after NAT10 inhibition, and the changes in metastasis ability of HCC cells and the efficacy of lenvatinib after NAT10 downregulation. The results showed that the deletion of the NAT10 gene significantly inhibited the tumorigenesis and metastasis of mouse HCC cells. In addition, immunohistochemistry also showed that NAT10 promoted ERS levels in HCC cells in vivo. Based on these results, we found that NAT10 promoted HCC metastasis and drug resistance through ERS at the cellular level. However, further studies are needed to determine its regulatory mode and NAT10 signal transduction pathway in HCC metastasis and drug resistance.

Previous studies have shown that ERS can reduce the sensitivity of HCC cells to doxorubicin, oxaliplatin, and sorafenib. Studies have shown that NAT10 can promote tumor metastasis and drug resistance through ERS, but the molecular mechanism is still unclear. NAT10 is an RNA ac4C-modifying enzyme, and whether mRNA ac4C modification is involved in the regulation of these processes remains unclear. Previous studies have shown that mRNA ac4C regulator NAT10 plays an important role in prognosis and immune function in pan-cancer [8]. Earlier studies suggested that ac4C modification mainly exists in non-coding RNA, such as tRNA and rRNA [4, 43, 44]. In 2018, Arango et al. first reported the existence of acetylated ac4C modification on mRNA to promote the efficiency of RNA translation [4]. In 2021, Zhang et al. found that NAT10 could promote the metastasis of gastric cancer through ac4C acetylation of COL5A1 [17], and Wei Rongfang et al. found that NAT10 could regulate cell proliferation and cell cycle distribution through acetylation of CEP170mRNA in multiple myeloma and improve translation efficiency [26]. In 2022, Cheng Yu et al. found that NAT10 can regulate the formation of angiointima. However, it remains unclear whether mRNA ac4C modification plays a regulatory role in the development and progression of human HCC. In order to further reveal the relevant biological mechanism, RNA-seq, and ACRIP-Seq were performed to identify HSP90AA1 as an intermediate molecular target for promoting drug resistance in ERS HCC during NAT10-mediated acetylation. The results showed that NAT10 binds and interacts with RNA molecules extensively, suggesting that NAT10 may play a key regulatory role in cell life activities through the action of RNA-binding protein. acRIP-seq combined with GOKEGG pathway enrichment analysis showed that after downregulation of NAT10, ac4C-modified genes in cell cycle pathway enrichment gene changes were more significantly and phosphatidylinositol 3-kinase signaling pathway. Dual-luciferase labeling assay showed that NAT10 regulated the expression level of HSP90AA1 through the ac4C modification pathway of mRNA, suggesting that NAT10 may play an important role in HCC metastasis through the ac4C gene.

Our team demonstrated that NAT10 could regulate the modification of HSP90AA1 mRNA ac4C through direct interaction. The biological effects of this rule, however, remain unclear. Quantitative polymerase chain reaction (QPCR), Western blotting (WB), and immunohistochemistry (IHC) were used to detect the expression of HSP90AA1 and the expression of the NAT10 gene. The results showed that the expression of NAT10 was positively correlated with that of HSP90AA1. Dual-luciferase reporter analysis also showed that NAT10 directly regulated HSP90AA1 expression, which was dependent on ac4C modification of the CDS coding region of the HSP90AA1 gene. According to these experimental results, we conclude that NAT10 can regulate the stability of HSP90AA1 mRNA and improve the efficiency of mRNA translation by regulating the level of mRNA ac4C to modify HSP90AA1 mRNA, thereby upregulating the expression level of HSP90AA1. The findings are consistent with those of the reported studies. ac4C modification of mRNA can upregulate the expression level of target genes [45].

mRNA ac4C modification is the core content of this study. The results of Arango et al. suggest that ac4C ON mRNA binds to the CDS region of mRNA [4]. In this study, two potential regulatory targets of NAT10 obtained through the ERS process of hepatoma cells screened by acRIP-Seq showed ac4C modification in the CDS region of mRNA. In addition, some mRNA in Huh-7 cells underwent ac4C modification after NAT10 downregulation. This study demonstrated that NAT10 maintained the stable expression of the HSP90AA1 gene and promoted its expression in HCC cells by mediating ac4C modification. However, the recognition proteins that interact with ac4C-modified HSP90AA1 mRNA to maintain its stability remain uninvestigated. HSP90AA1, a subtype of HSP90, is located in the cytoplasm and is also a functional gene of HSP90α. This study identified HSP90AA1 as a downstream target of NAT10-mediated mRNA ac4C modification in the regulation of HCC metastasis and ERS-related tumor resistance. However, the regulatory role of HSP90AA1 was initially unclear. GOKEGG pathway enrichment analysis showed that HSP90AA1 played an important role in the repair of vascular endothelial injury and intimal hyperplasia. Changes in the extracellular matrix play a key role in tumor angiogenesis and tumor metastasis [46–48]. This study showed that HSP90AA1 was positively correlated with the expression of ERS marker proteins GRP78, ATF-6, and PERK, and promoted the metastasis of HCC cells. HSP90AA1 also plays a downstream regulatory role in the proliferation and metastasis of gastric cancer [49]. It has been reported that HSP90AA1, a chronic obstructive pulmonary disease-related gene, can promote the progression of squamous cell lung cancer [35]. At present, studies have shown that changes in extracellular matrix components are important regulatory nodes for cell migration and invasion [47]. Unfortunately, this study is based only on cell-level experiments and bioinformatic predictions, focusing on the regulation of HSP90AA1 molecular processes by mRNA ac4C modification of NAT10. Studies have shown that HSP90AA1 plays an important role in the ERS process of HCC cells, and HSP90AA1 is located downstream of NAT10-HSP90AA1 regulatory axis. However, the specific mechanism by which HSP90AA1 regulates ERS in HCC was not explored in this study.

In conclusion, this study demonstrated that NAT10, an ac4C-modifying protein, plays an important role in HCC metastasis, lenvatinib resistance, and ERS of HCC cells by regulating the mRNA ac4C-modifying pathway. However, given that a large number of genes are involved in the ERS, metastasis, and drug resistance of HCC cells, it is not excluded that the NAT10-mediated mRNA ac4C pathway also affects the ERS and metastasis of HCC cells and drug resistance by regulating other genes. Remodelin, a specific small-molecule NAT10 inhibitor, was also found to inhibit cell proliferation and induce apoptosis of HCC cells. These data further reveal the potential value of Remodelin in future clinical treatment by reducing NAT10 activity. In conclusion, targeting NAT10 promotes proliferation and inhibits apoptosis by enhancing ERS in HCC cells, further causing drug resistance. Our data elucidate the oncogenic and drug-resistant role of NAT10 in HCC and highlight the importance of ac4C modification as a novel gene epigenetic regulatory pathway in tumor development, providing new insights into further mechanisms of tumorigenesis and progression.

## Materials and methods

### Patient specimens and immunohistochemical (IHC) assay

A total of 100 HCC patients were recruited from December 2017 to December 2021 in the First Affiliated Hospital of Anhui Medical University. HCC patients should be primary HCC, have not received any chemotherapy, intervention therapy, or immunity therapy before surgery, and with complete clinicopathological data. The experiment was approved by the Ethics Committee, and written informed consents were signed by each participant before participation. All tissue microarrays were composed of HCC tissues and matched normal tissues. IHC staining intensity was evaluated by IHC cytoplasmic staining intensity and the proportion of positive cells in the global visual field. Negative expression: no target protein staining or weak positive ≤20%. Low expression: weak positive staining area 20–80% or strong positive staining area ≤20%; High expression: weak positive staining area more than 80% or strong positive staining area ≥20%. The scoring standard of the immunohistochemical assay was listed in Supplementary Fig. S1.

### Cell culture

Human HCC cell lines Huh-7, hep3B, MHCC97h, and SK-Hep-1 were purchased from Guangzhou Seku Biotechnology Co., LTD. (Guangzhou, China). All cells were identified by STR for mycoplasma detection. Cultures were routinely cultured in DMEM (Hyclone, USA) containing 10% fetal bovine serum (Gibco) and 100 μ/ml each with penicillin and streptomycin (Gbico). The cells were cultured in 5% CO_2_ and humidified in an incubator at 37°C.

### Induced ERS

About 5 × 10^5^ HCC cells were seeded in 6-well plates and left overnight. About 3-μm TM (T8480; Solebo, Beijing, China) was added and co-cultured for 24 or 48 h.

### Cell proliferation assay

Huh-7 and Hep3B cells (1 × 10^4^ cells/well) were seeded in 96-well plates and cultured for 24 h. When the Cell volume was 70–80% for drug treatment and CCK-8 detection, cells were added with 10 μL/well Cell Counting KIT-8 (CCK-8; The whole gold, Beijing, China) solution was mixed with an oscillator and incubated for 2 h at 37 °C. Finally, the absorbance value at 450 nm was measured by a microplate reader, and the data were recorded. The inhibition rate of cell growth was: growth inhibition rate IR = [1 − (average OD value of experimental group)/(average OD value of control group) × 100%, and the IC50 value was calculated.

### siRNA

NAT10, HSP90AA1 and negative control small interfering RNA were synthesized by GenePharma (Shanghai, China). For lentiviral transfection, 1 × 10^6^ cells/well was seeded on six-well plates, diluted with 250 μl Opti-MEM for NC and siRNA (the final concentration of transfected cells was 100 nM). The transfection reagent was gently reversed and diluted with 250 μl Opti-MEM and 5 μl Lipofectamine 2000 (Thermo Fisher, USA) was mixed and allowed to stand for 5 min at room temperature. The transfection reagent and NC/siRNA diluent were mixed, and the mixture was allowed to stand for 20 min at room temperature. The transfection complex was added to a six-well plate (500 μL/well) and gently mixed before and after shaking. Transfection was terminated after 48 h of culture at 37 ° c in a 5% CO_2_ incubator.

### Western blot

According to the experimental purpose and the results of protein quantification of each sample, each protein sample was added to the corresponding Wells of SDS-PAGE. After electrophoresis, membrane transformation, and sealing, the PVDF membrane containing the target protein was incubated with the corresponding primary antibody at 4 °C overnight. The PVDF membrane was removed from the primary antibody, the secondary antibody was incubated, and the fluorescence signal was detected and imaged using an ImageQuant™LAS − 4000 luminescence imaging system. The antibodies in this study are shown in Supplementary Table S1.

### RNA extraction and qRT-PCR

The total RNA of cells or tissues was extracted by the Trizol-chloroform-isopropanol method, and the RNA with D260/D280 = 1.8–2.1 met the experimental requirements. Refer to the manual of Trizol total RNA Kit (The whole gold, Beijing, China). The DNA sequence of Primers in this study are shown in Supplementary Table S2.

### Liquid chromatography–tandem mass spectrometry

One microgram of the sample was added to the buffer, S1 nuclease, phosphodiesterase, and alkaline phosphatase, and RNA was completely enzymatically decomposed to nucleoside at 37 °C. The hydrolyzed sample was extracted with chloroform and the aqueous solution was added. The resulting solution was placed in injection vials for LC-ESI-MS/MS analysis. An ion flow chromatogram (XIC) was obtained. The molar content of the substance was obtained by substituting all detected integrated peak areas into the linear equation of the standard curve. The molar content of ac4C-modified nucleoside was calculated. Liquid chromatography–tandem mass spectrometry (LC-MS) was performed by Wuhan Metville Biotechnology Co., LTD.

### acRIP-Seq and acRIP-qPCR

We optimized the operating protocol of the Magna meriptmm6A kit (17-10,499, Million Wells) by replacing the m6A antibody with the ac4C antibody (AB252215, Abcam). acRIP-qPCR analysis was performed on NAT10 knockdown cells and ERS HCC cells. The concentration of total RNA was measured by Qubit RNA HS assay kit (Invitrogen, Q32852). Total RNA was extracted, and total RNA (200 μg) was randomly digested into 100–200nt nucleotide chains. After incubation at 70 °C for 6 min, the reaction was terminated immediately by the addition of EDTA, and a mixture of 5 μg of ac4C antibody and magnetic beads was incubated with the disrupted RNA. RNA was extracted from phenol-chloroform lysate and analyzed by qRT-PCR. The resulting products were removed with ribosomal RNA, the first-strand cDNA was synthesized by the SMART principle, and the library fragments were amplified by PCR. A DNA-purified magnetic bead library fragment was used to obtain a superfine RNA acetylated ac4C detection library. The Immunoglobulin G group was used as a negative control, and the input group was used as endogenous control. The library was prepared by EpiTM mini longRNA-seq kit (Epibiotek, E1802). Both the input samples without IP and the ac4C IP samples were subjected to 150-bp, paired-end sequencing on an Illumina NovaSeq 6000 sequencer. Acrip-qpcr primers are shown in Table S2. acRIP-seq was performed by Guangzhou Epigenetic Co., LTD.

### Immunofluorescence

Huh-7 and hep3B cells (3 × 10^5^/well) were seeded on cell climbing plates in 12-well plates. Cells were washed twice with PBS. Then 1 mL of 4% paraformaldehyde was added to each well and fixed at room temperature for 30 min. Then wash with PBS three times, 5 min/time. After absorbing and abandoning PBS, 500 μl PBSA (containing 1%BSA) of 0.2% Triton X-100 was added to each well for cell permeation, and the cells were placed on ice or in a 4-degree refrigerator for 5 min. After that, 1 mL PBSA was added to each well for blocking. After 1 h at room temperature, it was washed with PBS three times, 5 min/time. NAT10 and GRP78 protein were diluted with PBSA (dilution ratio 1:1000), 200 μL was added to each well, and incubated for 1 h at 37 °C in an incubator. Then, the FITC-labeled secondary antibody was diluted with PBSA (dilution ratio: 1:50), 200 μL was added to each well, and DAPI (final concentration: 10 ug/mL) was added, wrapped in tin foil, and incubated for 1H in an incubator at 37 °C. Then it was washed with PBS three times and then observed and photographed under an inverted fluorescence microscope.

### Luciferase reporter assay

After the successful construction of NAT10 knockdown and ERS Huh-7 cells, the cDNA containing the full-length coding region of HSP90AA1 CDS was cloned into the pmirGLO vector to obtain the wild-type vector. For mutant plasmids, to predict potential ac4C mutation sites, C was replaced by G in the CAC ac4C sequence of the CDS coding region of the HSP90AA1 gene. Dual-luciferase reporter assay system (Promega) was used to detect luciferase activity. The double luciferase assay was provided by Shanghai Jiman Biotechnology Co., LTD

### Wound healing assay

The cells were seeded in a scratch chamber at 5 × 10^4^/well, and the scratch chamber was placed in a 24-well plate. Drug administration and transfection were carried out when the degree of cell fusion was 90%. The cells were cultured at 37 °C and 5% CO_2_ incubator, and the photos were taken at 0, 24, and 48 h after scratch. A 24-well cell culture plate was taken, and a 600 μl complete culture medium containing 10%FBS was added to each well.

### Transwell migration and invasion assay

Transwell chamber (corning) with 8μm pore size PET film. According to experimental groups, 200 μL cell suspension/well was added for transfection and then fixed with 4% paraformaldehyde for 15 min. After staining with 0.1% crystal violet solution for 20 min, penetrating cells in three random areas were counted under an inverted microscope (100×) and photographed for preservation. The only difference between the Transwell invasion assay and the migration assay is that the top layer of the PET membrane in the Transwell chamber is uniformly covered with matrigel.

### Cell cycle assay

Cells were collected and washed with PBS (centrifuged at 2000 rpm for 5 min), the cell concentration was adjusted to 1 × 10^6^/ml, and 1 ml single-cell suspension was taken. The prepared single-cell suspension was centrifuged, the supernatant was removed, and the cells were fixed by adding 500 μl of 70% cold ethanol (volume fraction) for 2 h overnight. The fixed solution was washed with PBS before staining. Centrifuge at 2000 rpm for 5 min. About 500 μL of PI/RNase A staining solution was added, and the solution was kept away from light for 30–60 min at room temperature. The red light fluorescence at the excitation wavelength of 488 nm was recorded.

### Flow cytometry

huh-7 and Hep3B cells (1 × 10^6^ cells/well) were tiled and cultured in six-well plates for 24 h, and transfected and administered when the degree of cell confluence was 50–60%. Cells were digested with trypsin (trypsin without EDTA), washed with PBS, and apoptotic cells were stained with Annexin V-FITC Apoptosis Detection Kit (Thermo Scientific, Shanghai, China) according to the manufacturer’s instructions. Cells were harvested, resuspended, and counted in PBS, centrifuged at 1000×*g* for 5 min, the supernatant was discarded, and cells were resuspended by adding 195 µl Annexin v-fitc binding solution. Then 5 µL Annexin V-FITC staining solution was added, mixed, and 10 µL PI staining solution was added, and gently mixed. Incubate for 10–20 min at room temperature (20–25 °C) in the dark and place in an ice bath. Cells could be resuspended two to three times during incubation to improve staining and were analyzed by BDTM LSR ϩ ŝ flow cytometry (BD Biosciences) with Annexin V-FITC as green fluorescence and propidium iodide (PI) as red fluorescence. Subsequently, samples were measured and data recorded with Cell Quest (BD Bioscience, San Jose, CA, USA) software.

### Animal experiment

Balb/ C nude mice (3-4 weeks old, *n* = 42) were purchased from Sbef Biological Co., LTD. (Beijing, China). Mice were randomly divided into a control group and a NAT10 inhibition group. Huh-7 cells (*n* = 6, 2 × 10^6^/100 µl) were collected and injected subcutaneously into the lateral right thigh. When the tumor volume reached 100 mm^3^, mice in the NAT10 inhibitor group were intraperitoneally injected with Remodelin solution (5 mg/kg) every 2 days, while mice in the normal group were injected with DMSO. The body weight and tumor diameter (long diameter A, short diameter B) of mice were recorded every 2 days, and the tumor volume was calculated as V = (A × B2)/2. After 21 days, the mice were sacrificed, and the tumors were collected for HE, IHC (NAT10, HSP90AA1, GRP78), WB, and qPCR detection (NAT10, HSP90AA1). In lenvatinib drug experiment, nude mice were randomly divided into the Control group, lenvatinib group, and NAT10 inhibition group to observe the effect of NAT10 on tumor drug resistance. The same was true for the control group (NC) and the Lenvatinib and HSP90AA1 knockdown groups. Each mouse was intraperitoneally injected with Huh-7 cells (2 × 10^6^/100 µL) in the right thigh. When the tumor grew to 100 mm^3^, Lenvatinib (10 mg/kg) was injected intraperitoneally every Tuesday, Thursday, and Saturday. After 21 days, the mice were sacrificed and the tumor size was observed. When evaluating the results, the researchers did not know the groups. Animal experiments were approved by the Animal Ethics Committee of Anhui Medical University.

### Statistic analysis

All data were expressed as mean ± standard deviation. *T*-test was used to compare the means of two samples, ANOVA was used to compare the means of multiple groups, and a chi-square test or rank-sum test was used to analyze the relationship between NAT10 expression and clinicopathological features of patients. Kaplan–Meier (log-rank) survival curve was used to compare the survival difference between patients in the high NAT10 expression group and patients in the low NAT10 expression group. A *p* value less than 0.05 was considered statistically significant.

## Supplementary information

Table S1

Table S2

Table S3

Figure S1

Quantified results and statistical analysis of Western blot

Quantified results and statistical analysis of immunohistochemical assay

Original Data File

## Data Availability

The datasets used and analyzed during the current study are available from the corresponding author on reasonable request.
