# Supplementary material for: Role of NAT10-mediated ac4C-modified HSP90AA1 RNA acetylation in ER stress-mediated metastasis and lenvatinib resistance in hepatocellular carcinoma
Source: Cell Death Discov. 2023 Feb 10;9:56. doi: 10.1038/s41420-023-01355-8 (PMC9918514; doi:10.1038/s41420-023-01355-8)
Supplement: Supplementary file 1 — Table S1 [file 41420_2023_1355_MOESM1_ESM.docx]

Table S1. Antibody list

| Antibody | Concentration | | Company | Catalog Number |
| --- | --- | --- | --- | --- |
|  | Western blot | IHC |  |  |
| ATF-6 | 1:1000 | 1:50 | Cell Signaling Technology | 65880T |
| GRP78 | 1:1000 | 1:200 | Cell Signaling Technology | 3177T |
| IRE1α | 1:1000 | 1:50 | Cell Signaling Technology | 3294T |
| PERK | 1:1000 | 1:50 | Cell Signaling Technology | 5683T |
| NAT10 | 1:1000 | 1:500 | Abcam | ab194297 |
| HSP90AA1 | 1:1000 | 1:300 | Abnova | PAB10219 |
| Bak | 1:1000 | NA | Cell Signaling Technology | 12105T |
| Bax | 1:1000 | NA | Cell Signaling Technology | 5023T |
| Bcl-2 | 1:1000 | NA | Cell Signaling Technology | 3498T |
| CDK2 | 1:1000 | NA | Cell Signaling Technology | 18048S |
| CyclinA | 1:1000 | NA | Cell Signaling Technology | 67955S |
| PCNA | 1:1000 | NA | Cell Signaling Technology | 13110S |
| GAPDH | 1:3000 | NA | Solarbio | M1000110 |

NA: Not applicable
