# Supplementary material for: Role of NAT10-mediated ac4C-modified HSP90AA1 RNA acetylation in ER stress-mediated metastasis and lenvatinib resistance in hepatocellular carcinoma
Source: Cell Death Discov. 2023 Feb 10;9:56. doi: 10.1038/s41420-023-01355-8 (PMC9918514; doi:10.1038/s41420-023-01355-8)
Supplement: Supplementary file 2 — Table S2 [file 41420_2023_1355_MOESM2_ESM.docx]

Table S2. DNA sequence of Primers of qRT-PCR in our study

| **Primer** | **Sequence（5'-3'）** |
| --- | --- |
| NAT10-F | -CTGTCTGGTGGAAGGGTCGTT- |
| NAT10-R | -GCCTCGCTGCTTACGGTGT- |
| HSP90A-F | 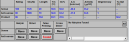-GCCCTTCTATTTGTCCCACG- |
| HSP90A-R | -TCCTCCGAGTCTACCACCC- |
| GAPDH-F | -AATCCCATCACCATCTTCCA- |
| GAPDH-R | -AAATGAGCCCCAGCCTTCT- |
