# Supplementary material for: Role of NAT10-mediated ac4C-modified HSP90AA1 RNA acetylation in ER stress-mediated metastasis and lenvatinib resistance in hepatocellular carcinoma
Source: Cell Death Discov. 2023 Feb 10;9:56. doi: 10.1038/s41420-023-01355-8 (PMC9918514; doi:10.1038/s41420-023-01355-8)
Supplement: Supplementary file 3 — Table S3 [file 41420_2023_1355_MOESM3_ESM.docx]

Table S3. Spearman rank correlation between the expression of NAT10 and ERS marker proteins in IHC

| Gene | Number | Negative | Low expression | High expression | *r_s_* | P |
| --- | --- | --- | --- | --- | --- | --- |
| NAT10 | 100 | 8 | 35 | 57 | - | - |
| GRP78 | 100 | 2 | 19 | 79 | 0.472 | < 0.001 |
| ATF-6 | 100 | 3 | 24 | 73 | 0.302 | < 0.001 |
| IRE-1 | 100 | 4 | 25 | 71 | 0.446 | < 0.001 |
| PERK | 100 | 4 | 40 | 56 | 0.410 | < 0.001 |
