# Supplementary figures and images for: Role of NAT10-mediated ac4C-modified HSP90AA1 RNA acetylation in ER stress-mediated metastasis and lenvatinib resistance in hepatocellular carcinoma
Source: Cell Death Discov. 2023 Feb 10;9:56. doi: 10.1038/s41420-023-01355-8 (PMC9918514; doi:10.1038/s41420-023-01355-8)

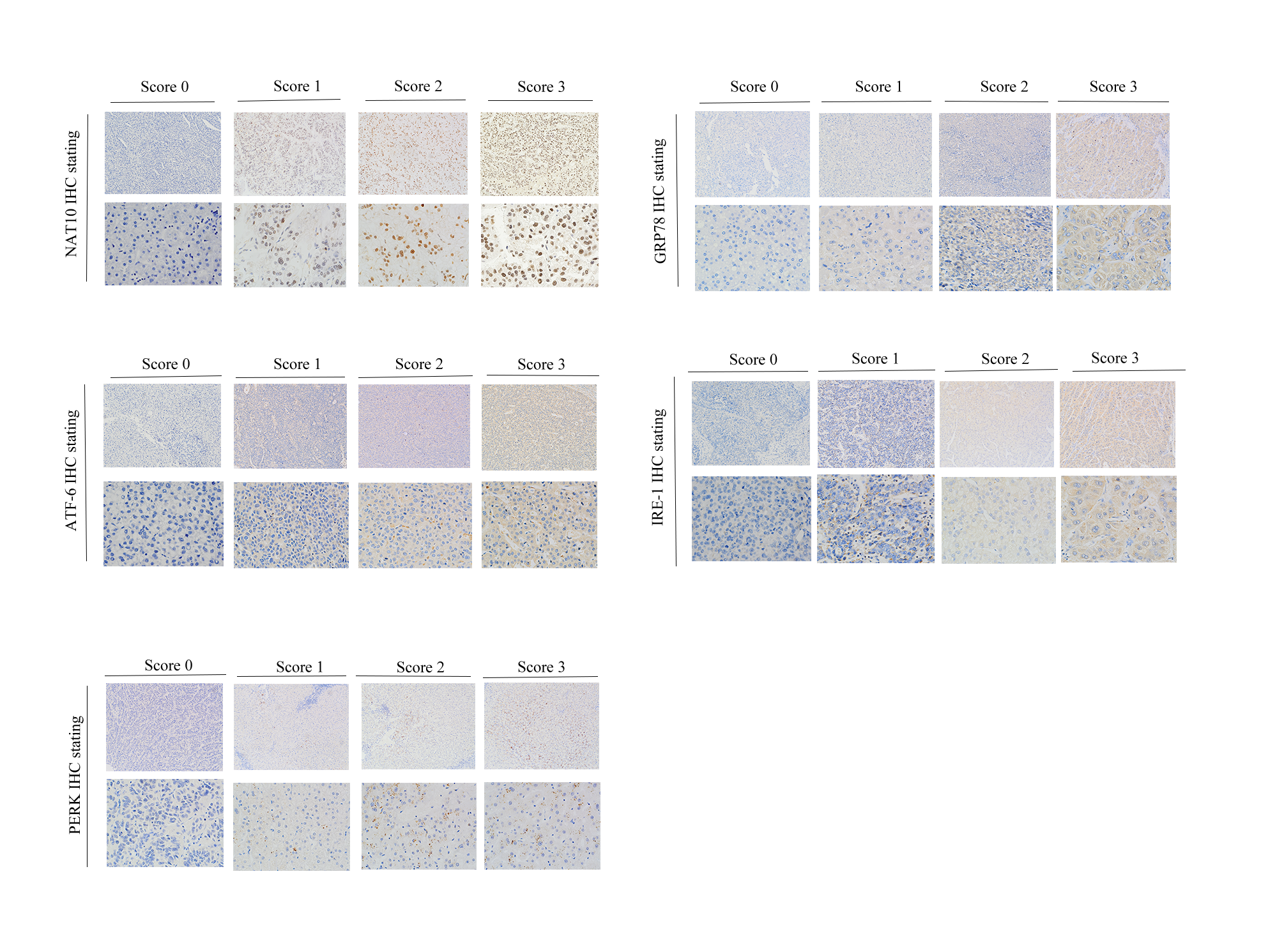


Figure S1. The scoring standards of immunohistochemical assay

Supplement: Supplementary file 4 — Figure S1 [file 41420_2023_1355_MOESM4_ESM.docx]
