# Supplementary material for: Role of NAT10-mediated ac4C-modified HSP90AA1 RNA acetylation in ER stress-mediated metastasis and lenvatinib resistance in hepatocellular carcinoma
Source: Cell Death Discov. 2023 Feb 10;9:56. doi: 10.1038/s41420-023-01355-8 (PMC9918514; doi:10.1038/s41420-023-01355-8)
Supplement: Supplementary file 5 — Quantified results and statistical analysis of Western blot [file 41420_2023_1355_MOESM5_ESM.docx]

**Quantified results and statistical analysis of WB**

**Table of contents**

**Figure 1E**

**Figure 2E**

**Figure 2F**

**Figure 2G**

**Figure 3F**

**Figure 4D**

**Figure 5L**

**Figure 5M**

**Figure 6A**

**Figure 6H**

**Figure 7F**


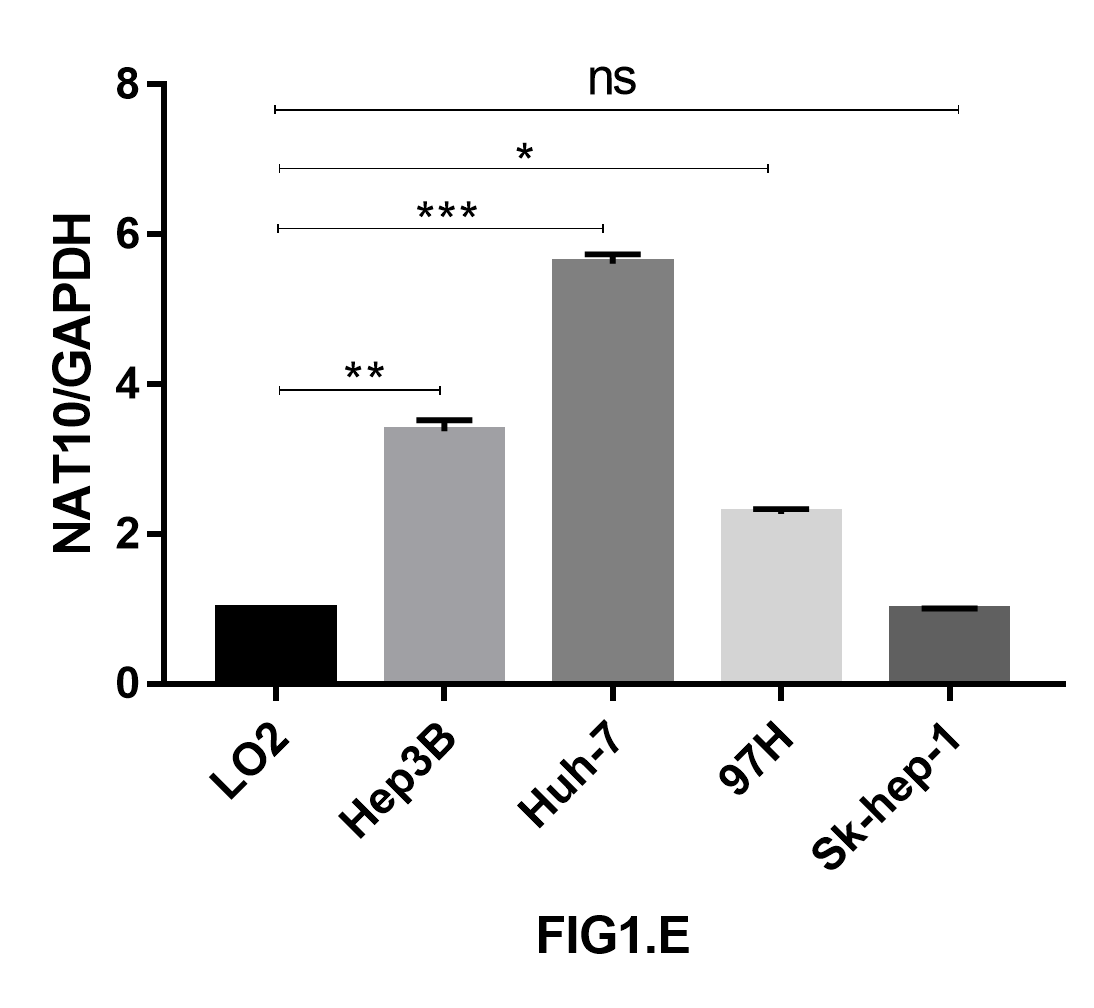


Fig 1E. Hepatocellular carcinoma cell-NAT10


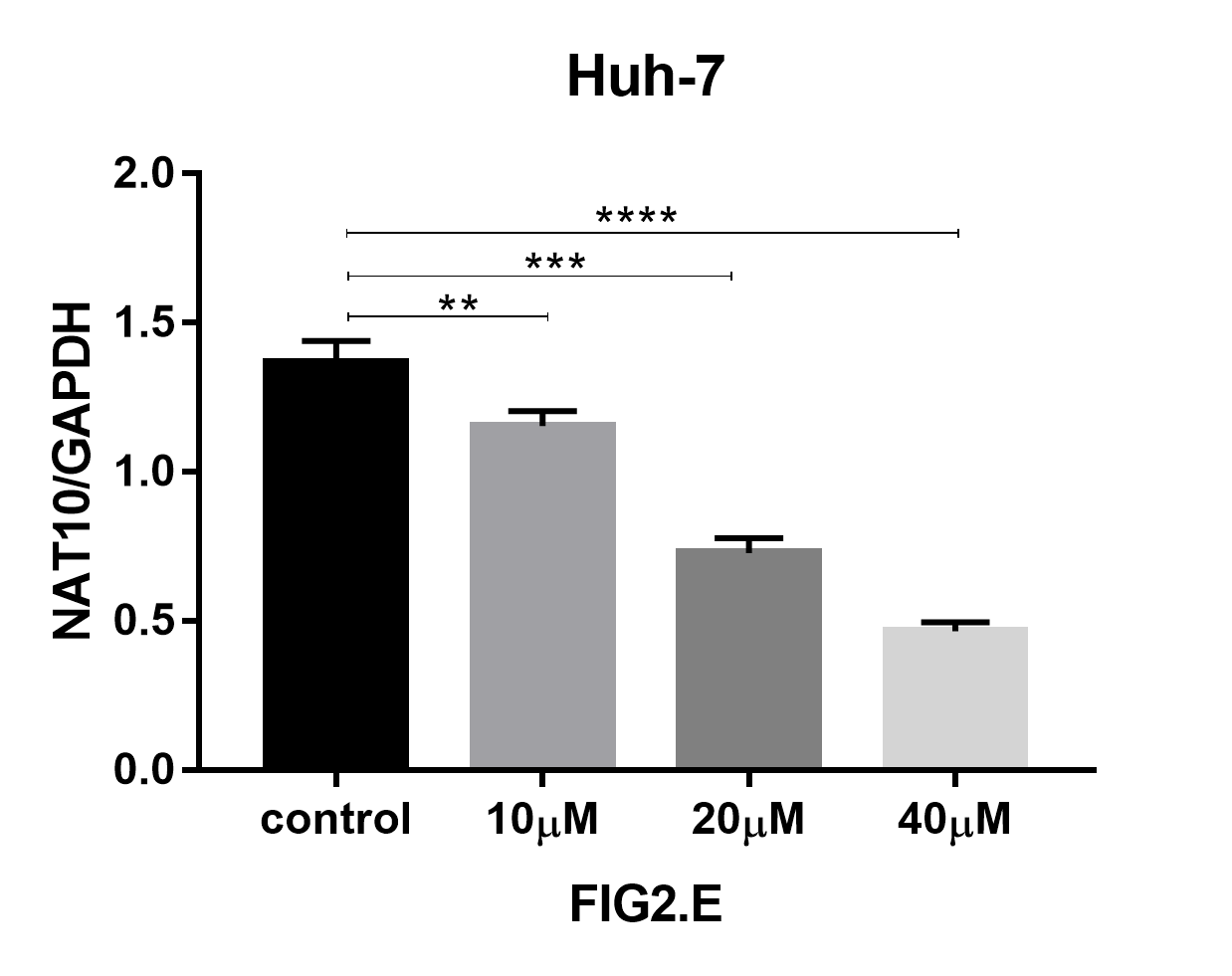


Fig 2E. huh-7-NAT10


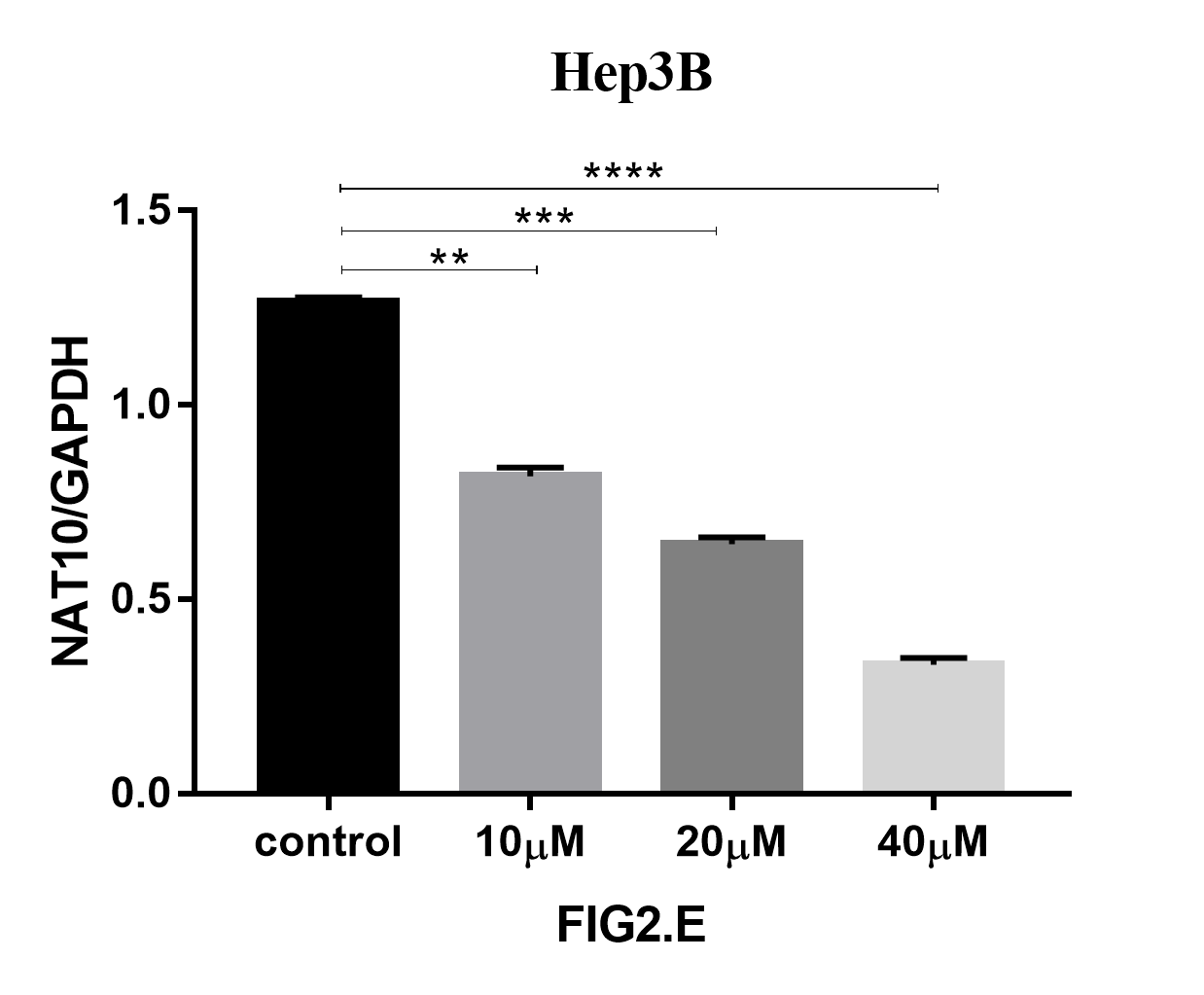


Fig 2E. hep3B-NAT10


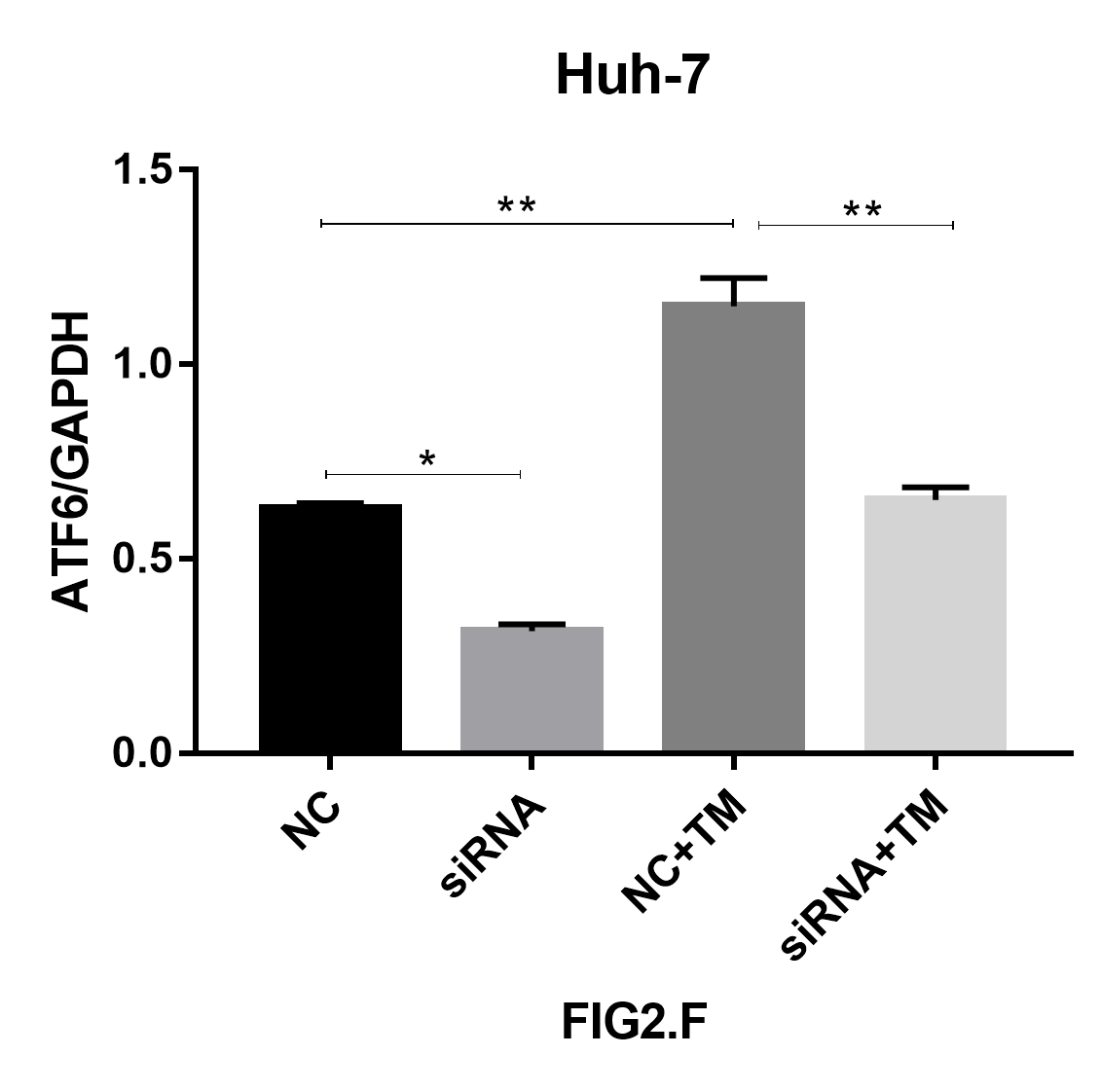


Fig 2F. huh-7-ATF6


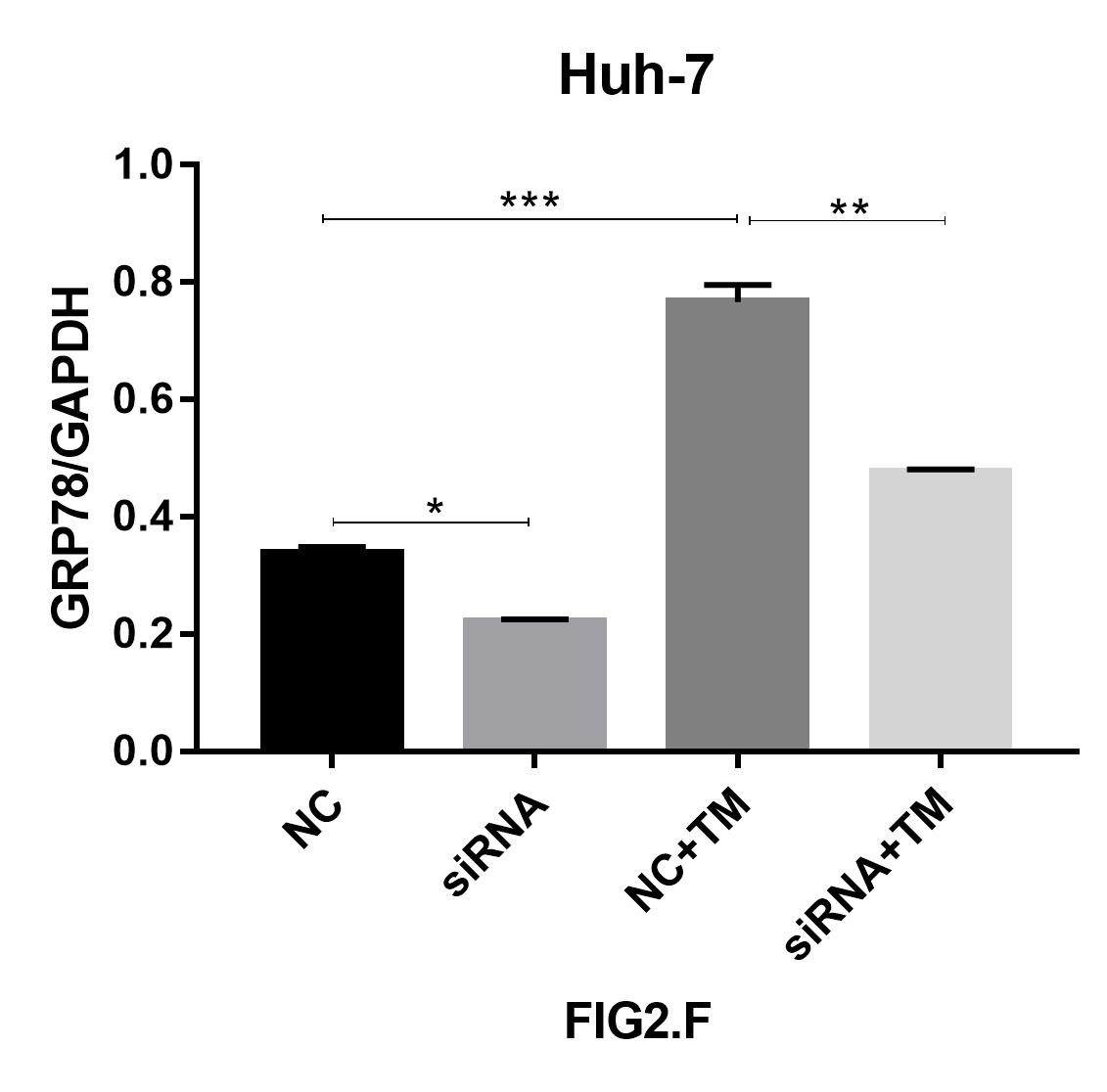


Fig 2F. huh-7-GRP78


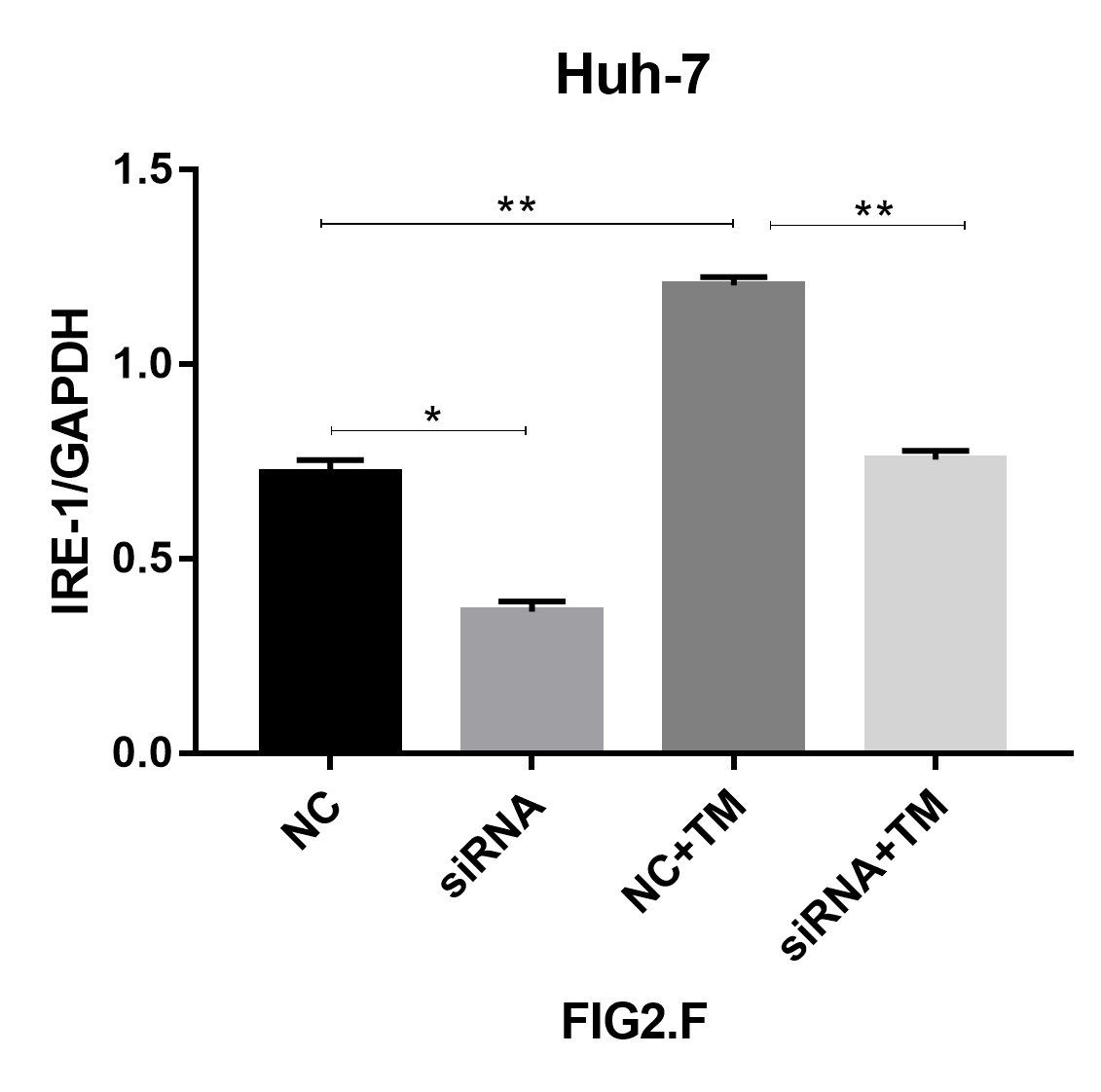


Fig 2F. huh-7-IRE-1


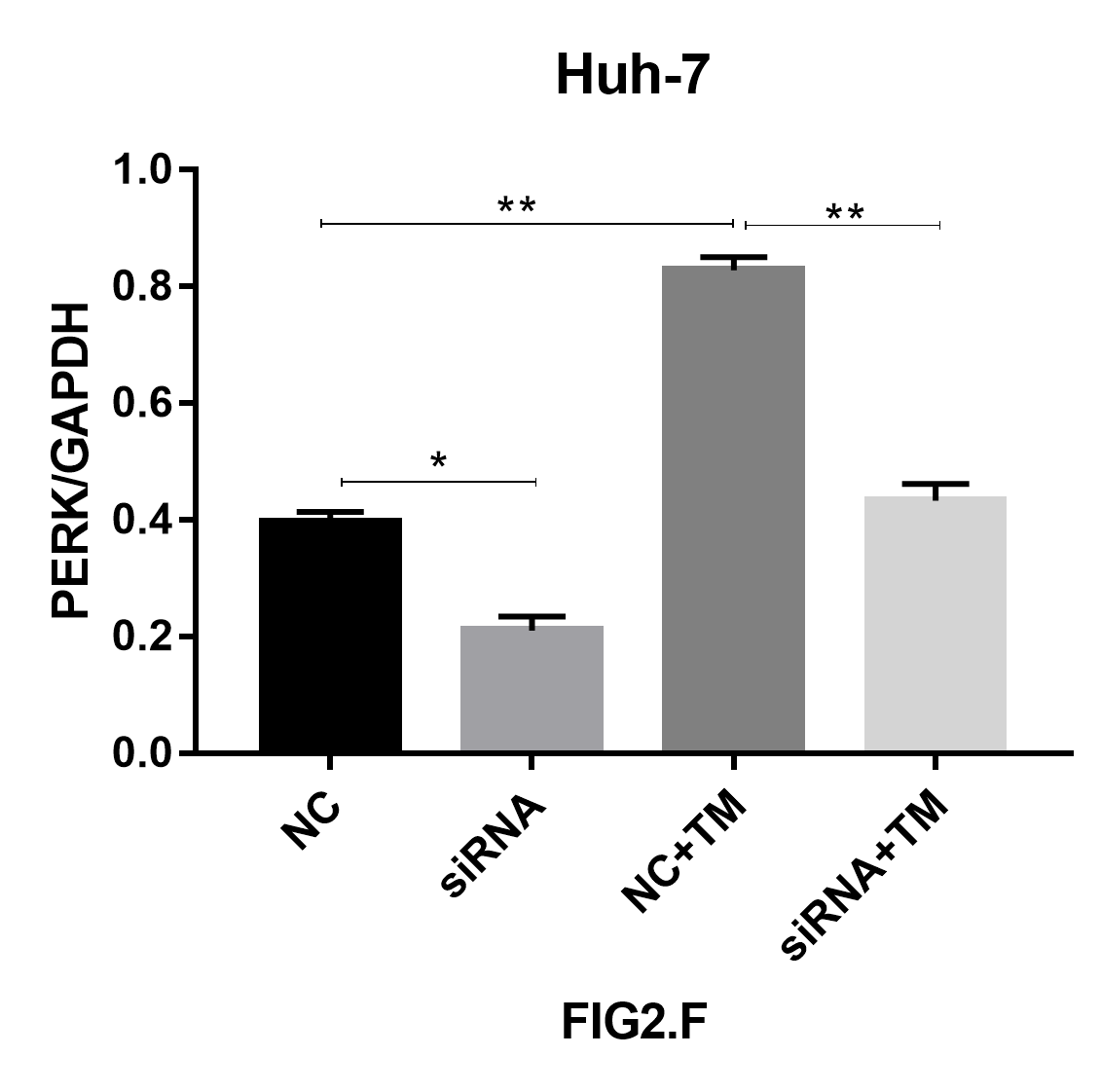


Fig 2F. huh-7-PERK


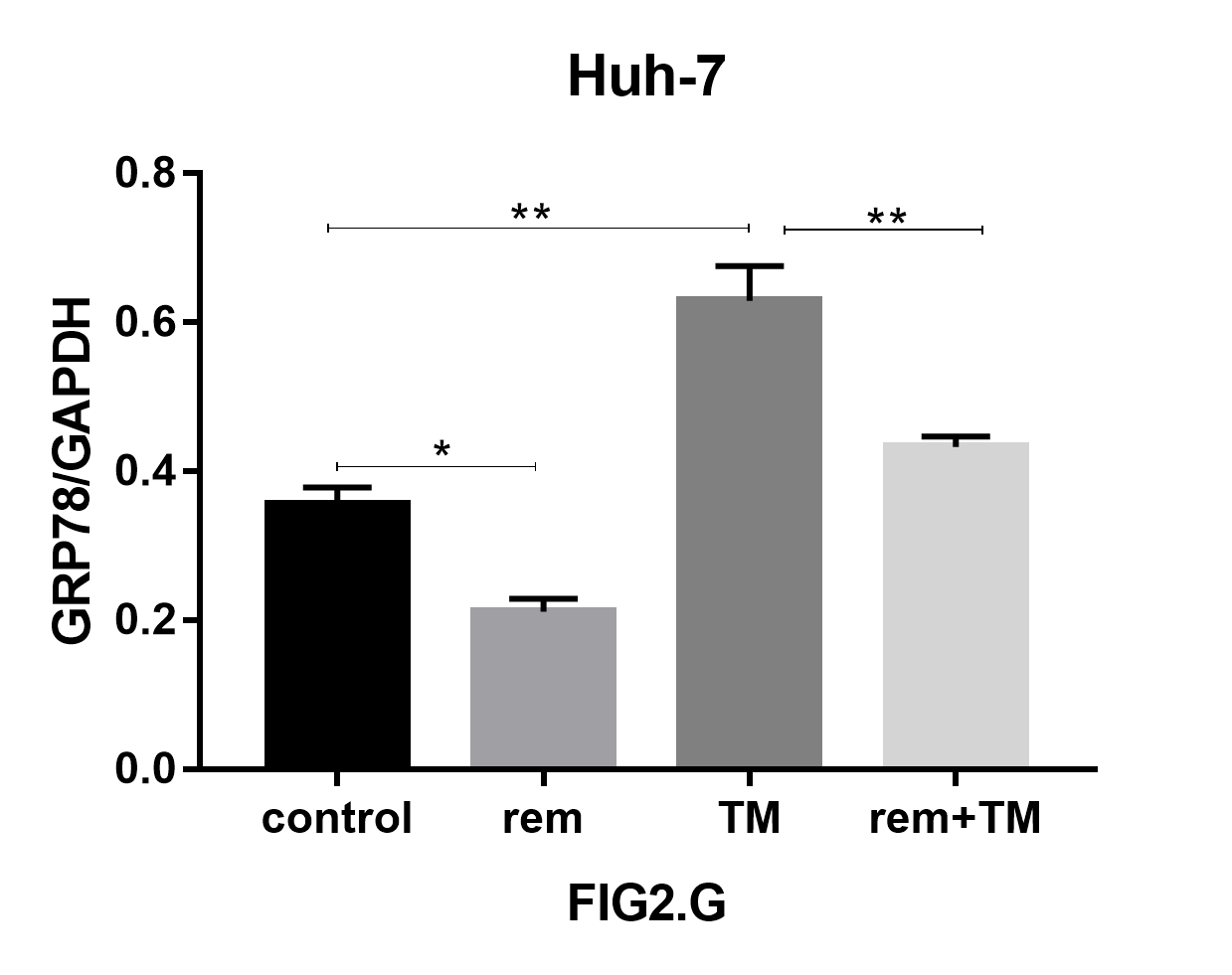


Fig 2G. huh-7-GRP78


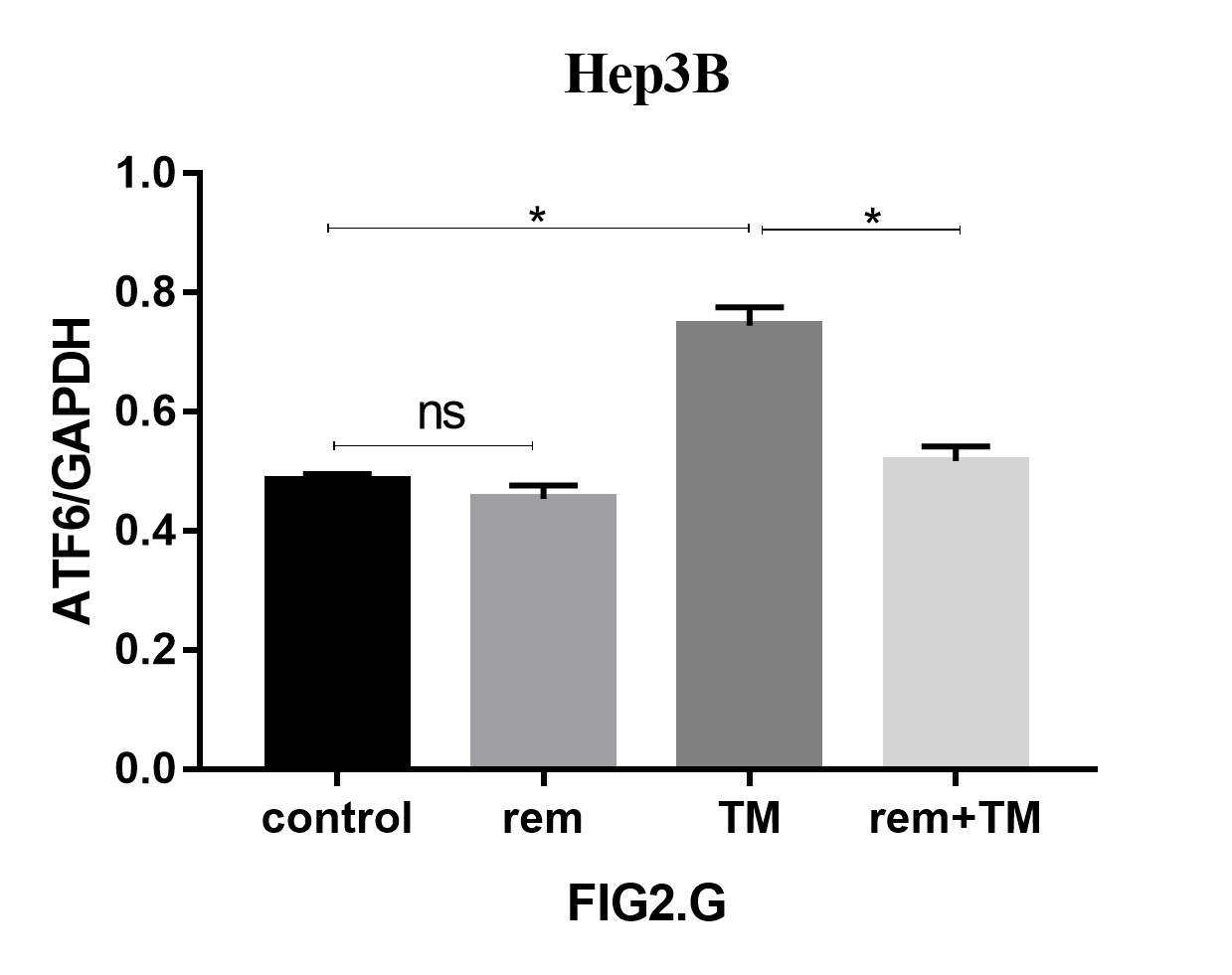


Fig 2G. hep3b-ATF6


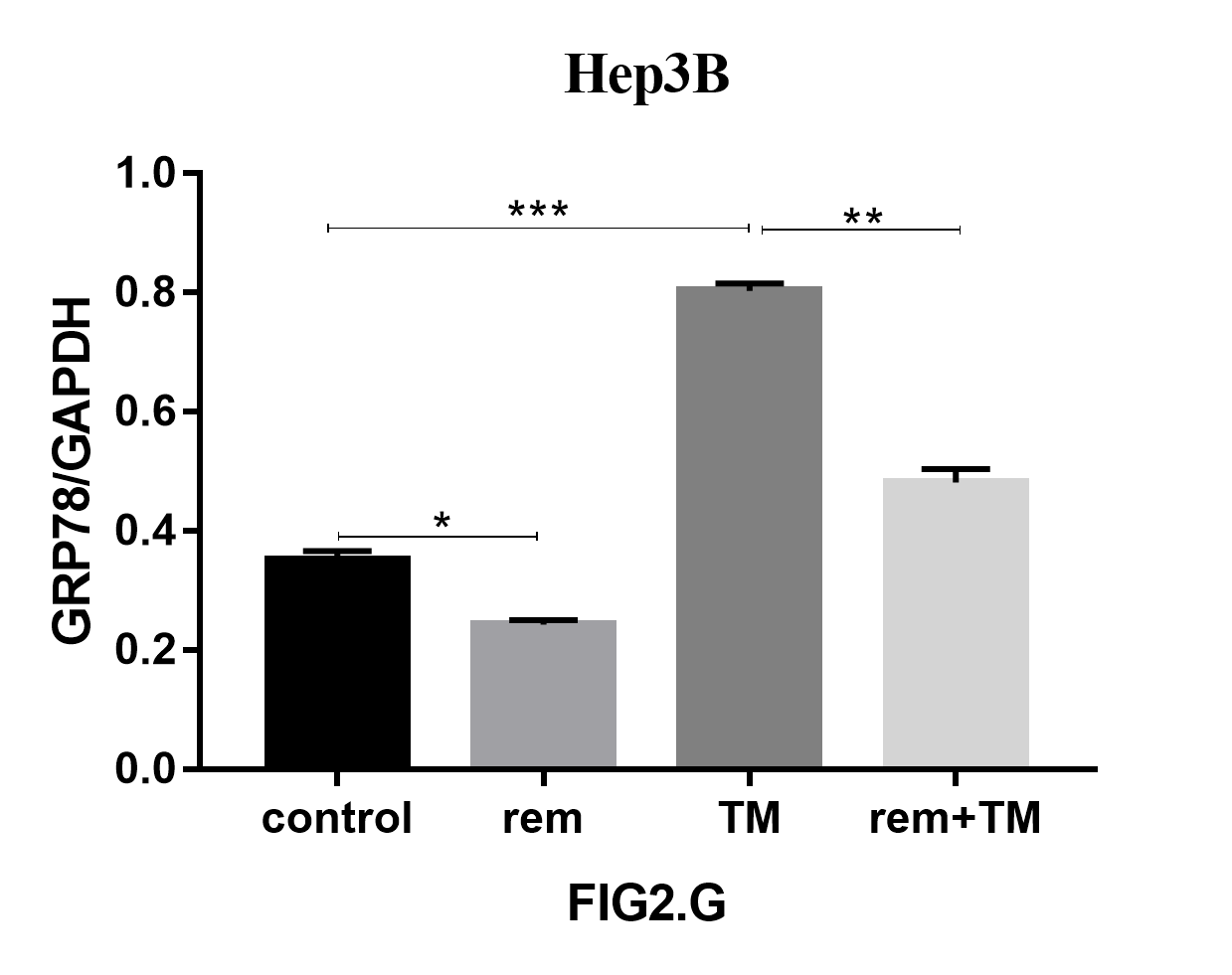


Fig 2G. hep3b-GRP78


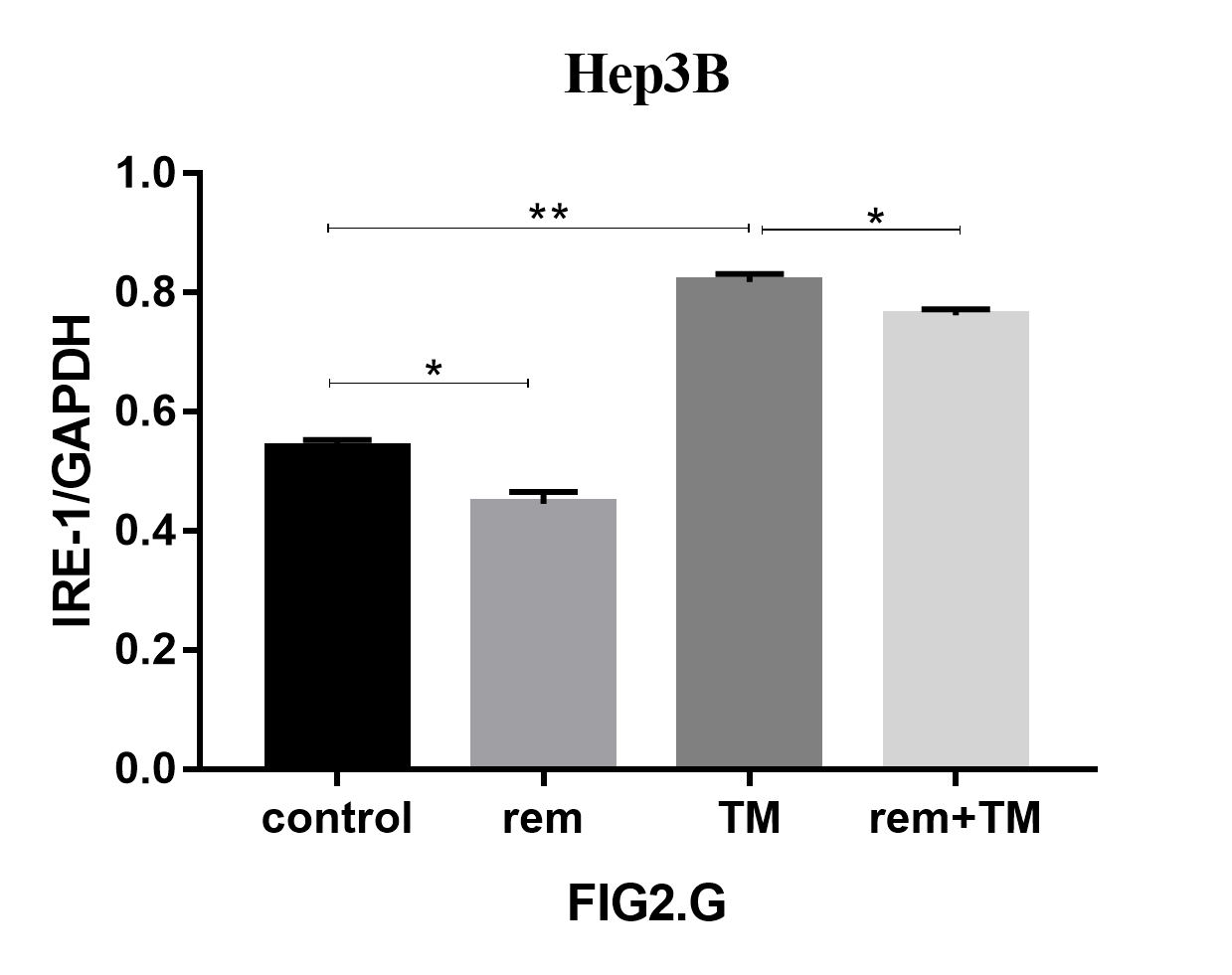


Fig 2G. hep3b-IRE-1


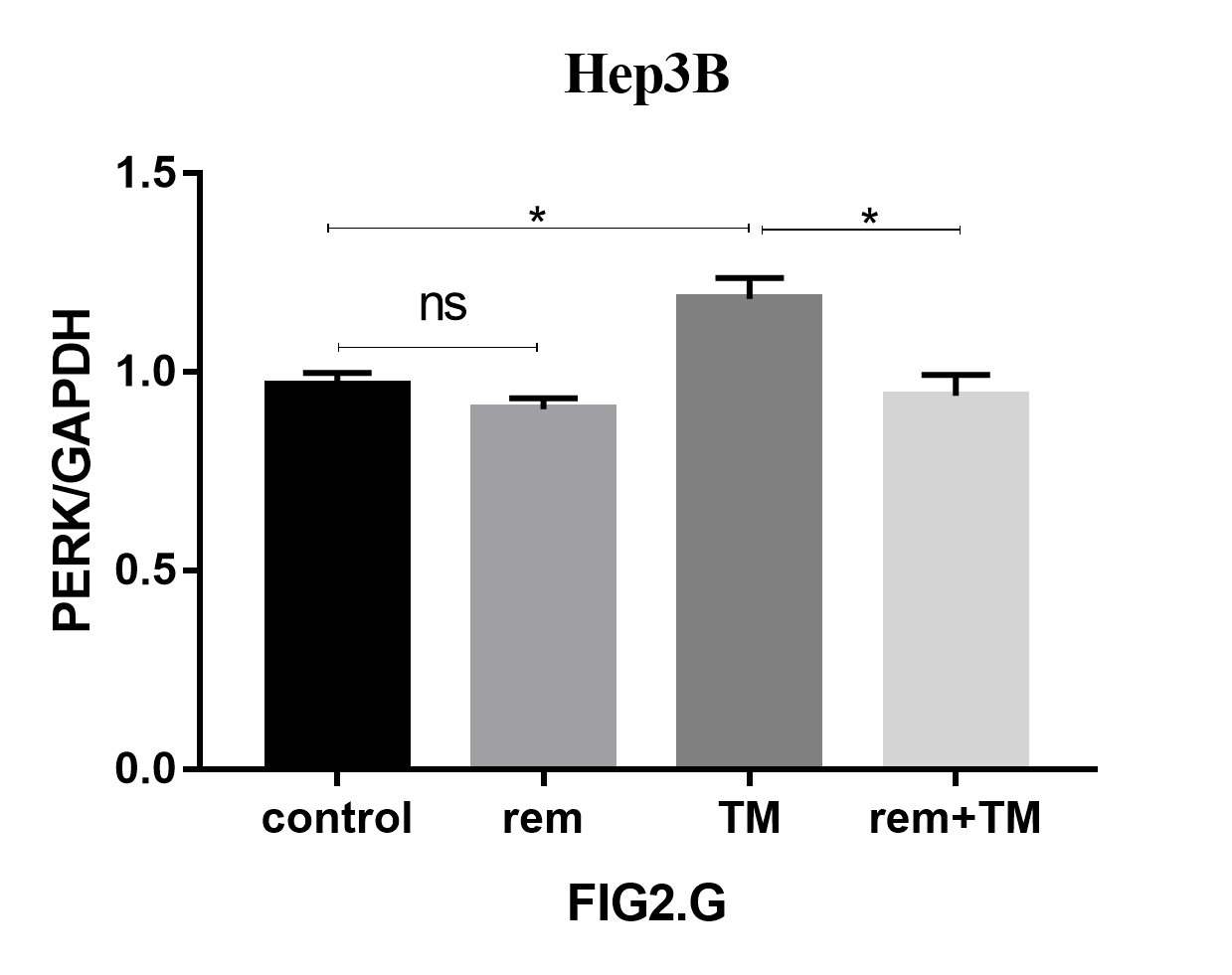


Fig 2G. hep3b-PERK


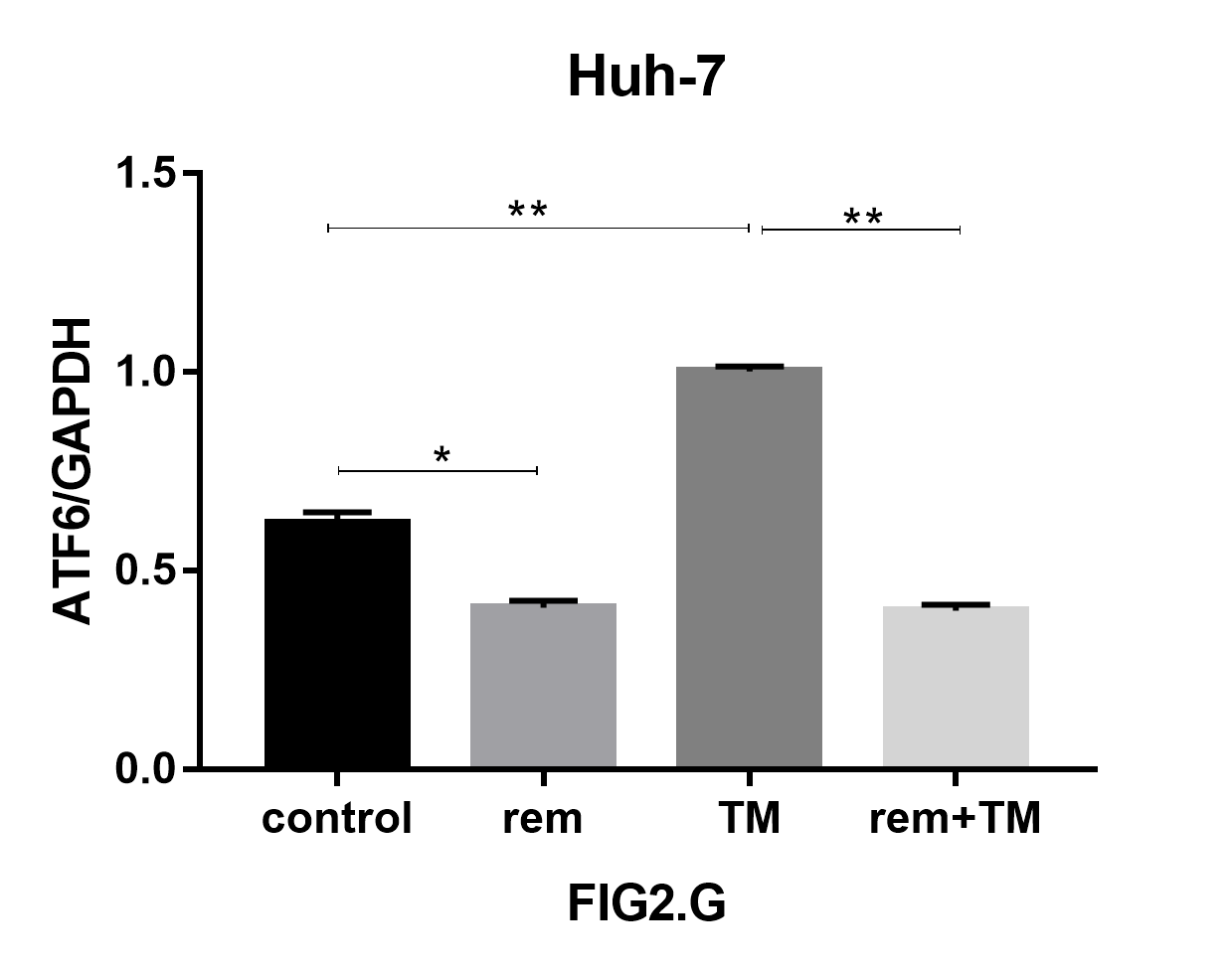


Fig 2G. huh-7-ATF6


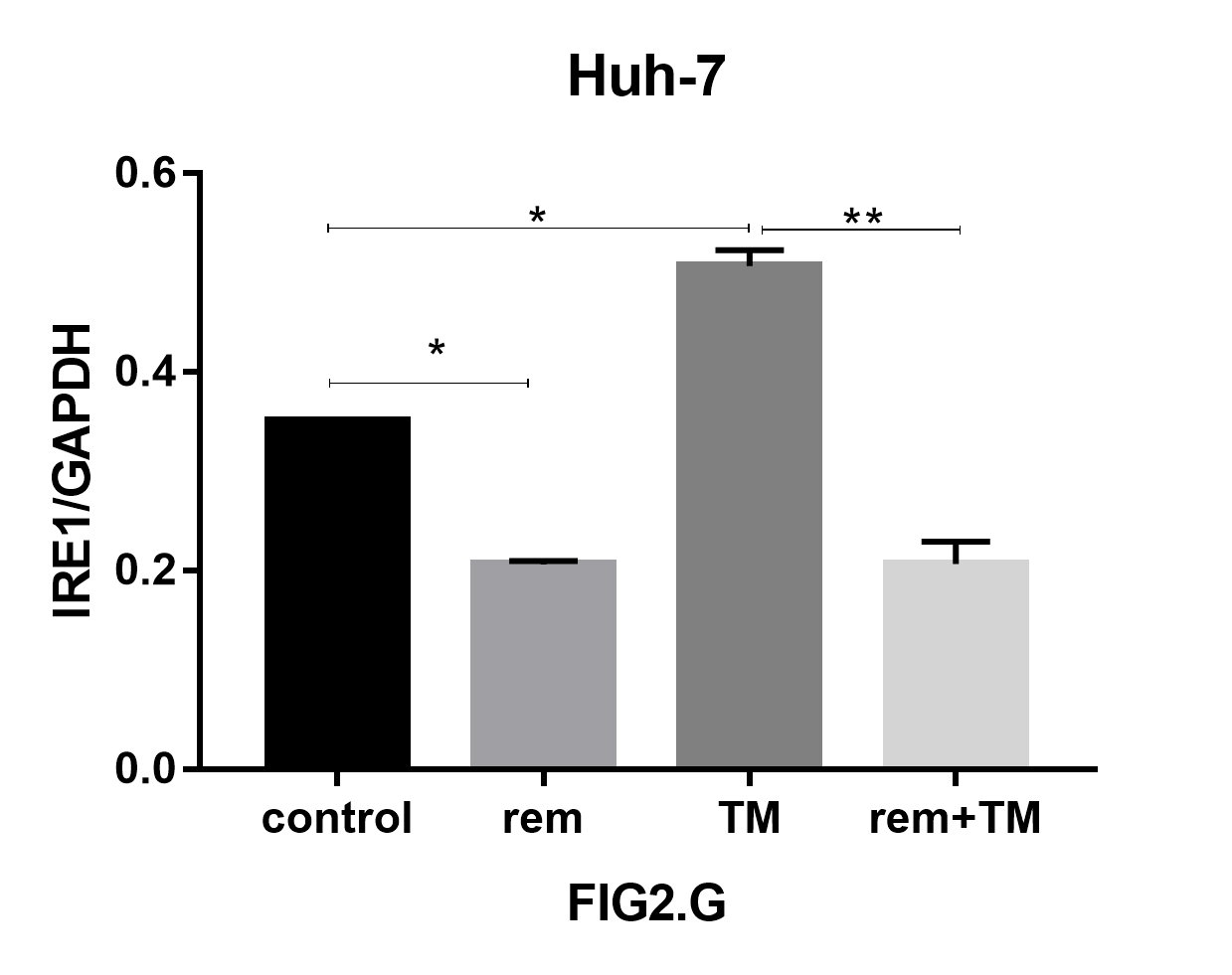


Fig 2G. huh-7-IRE-1


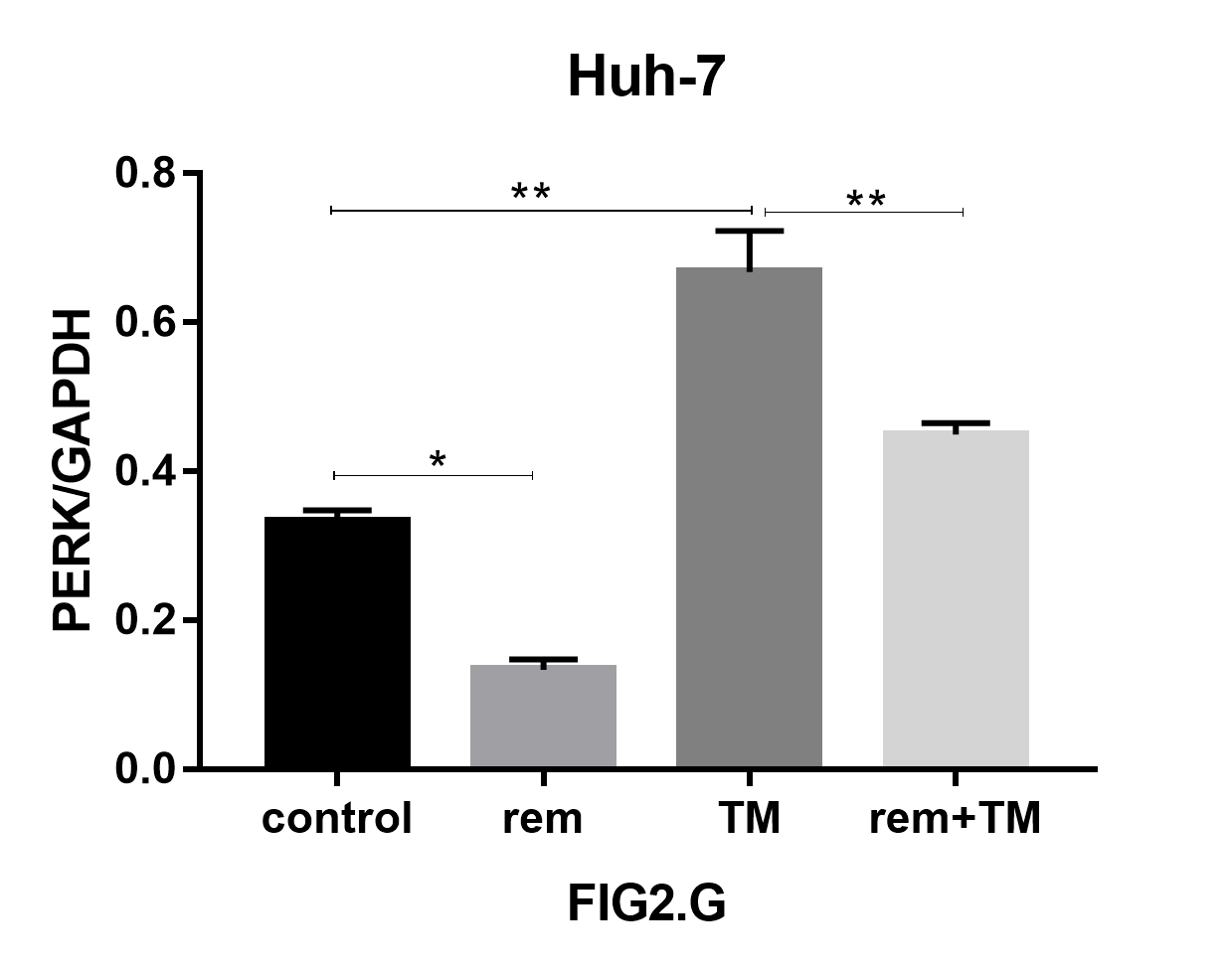


Fig 2G. huh-7-PERK


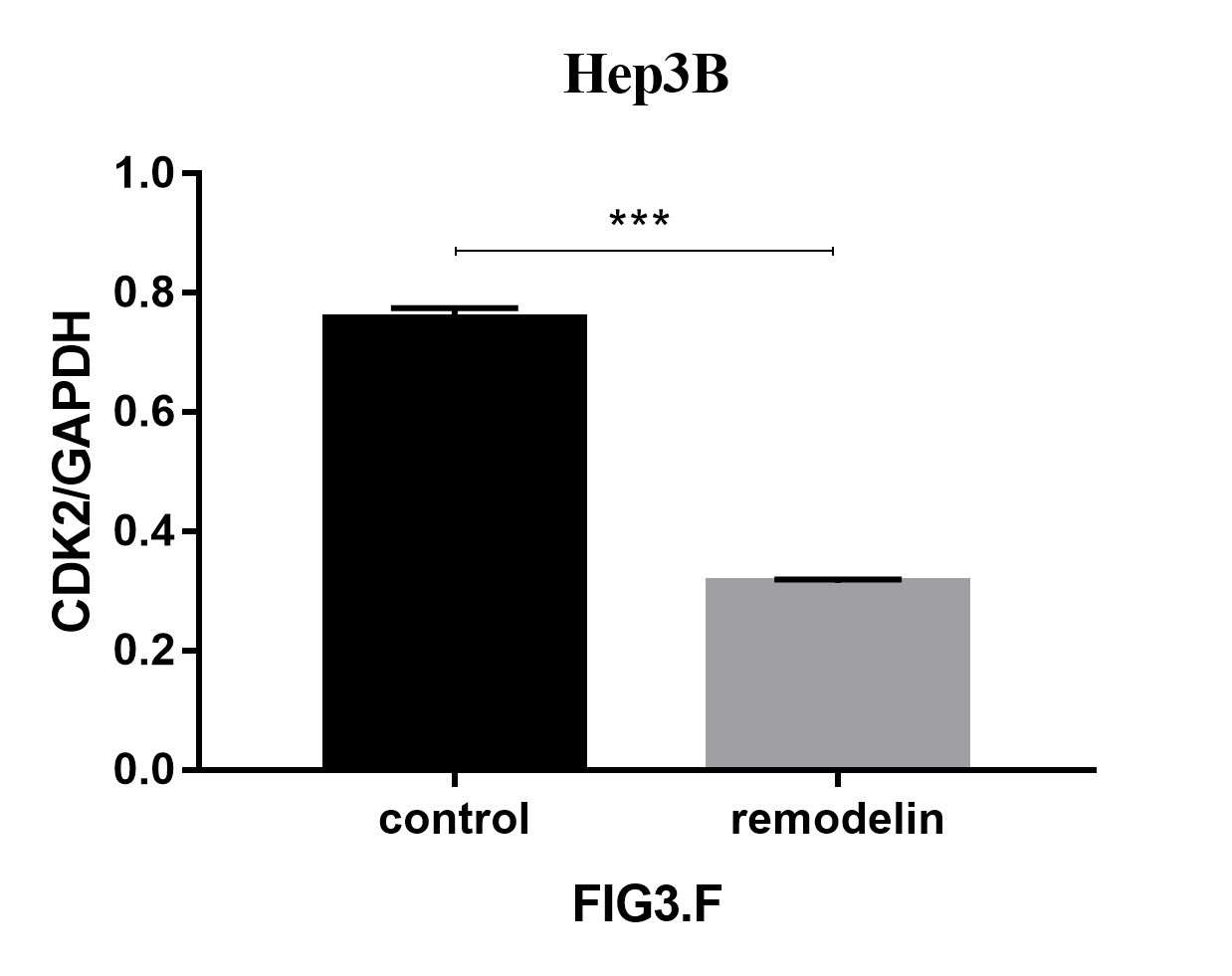


Fig 3F. hep3b-CDK2


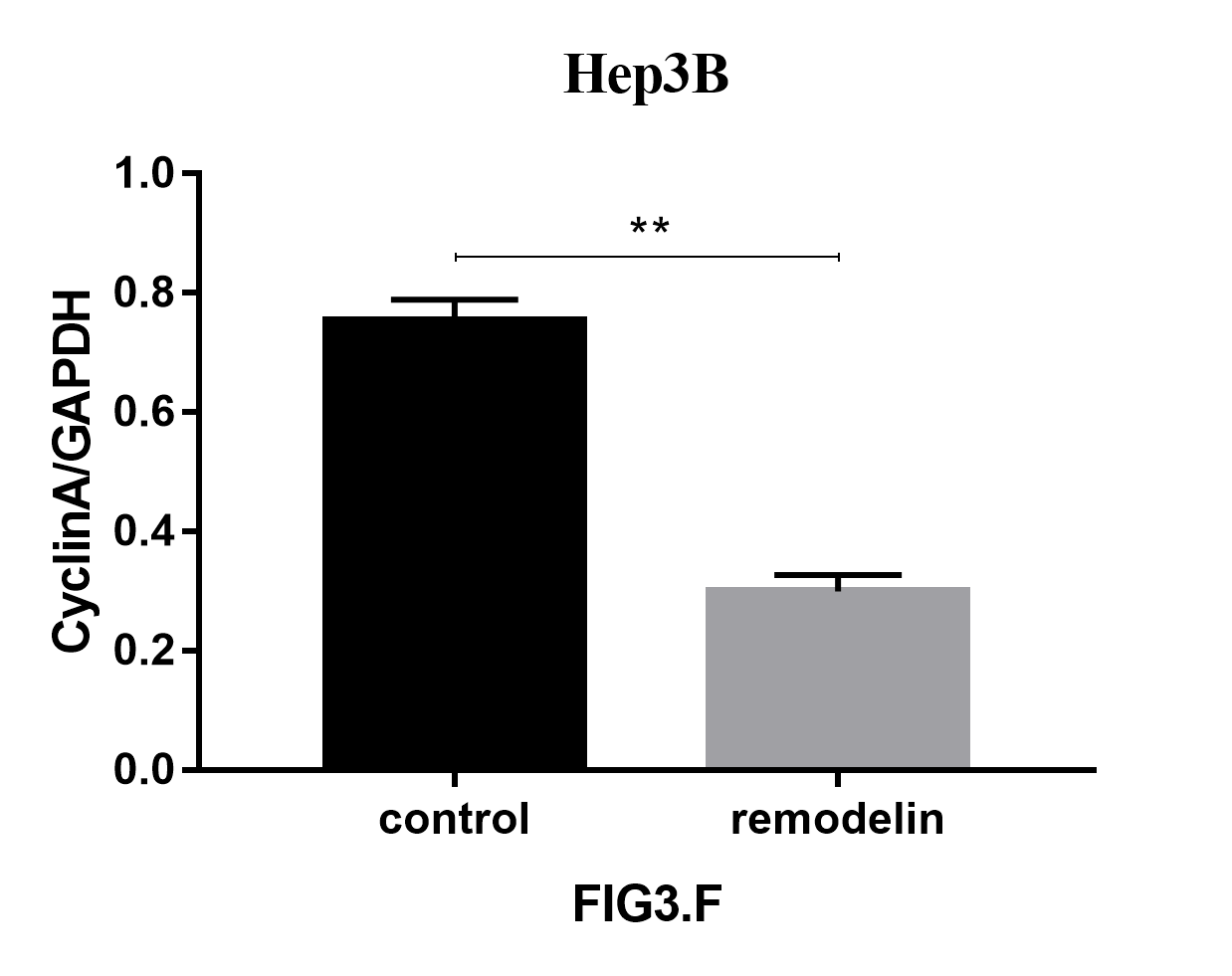


Fig 3F. hep3b-CyclinA


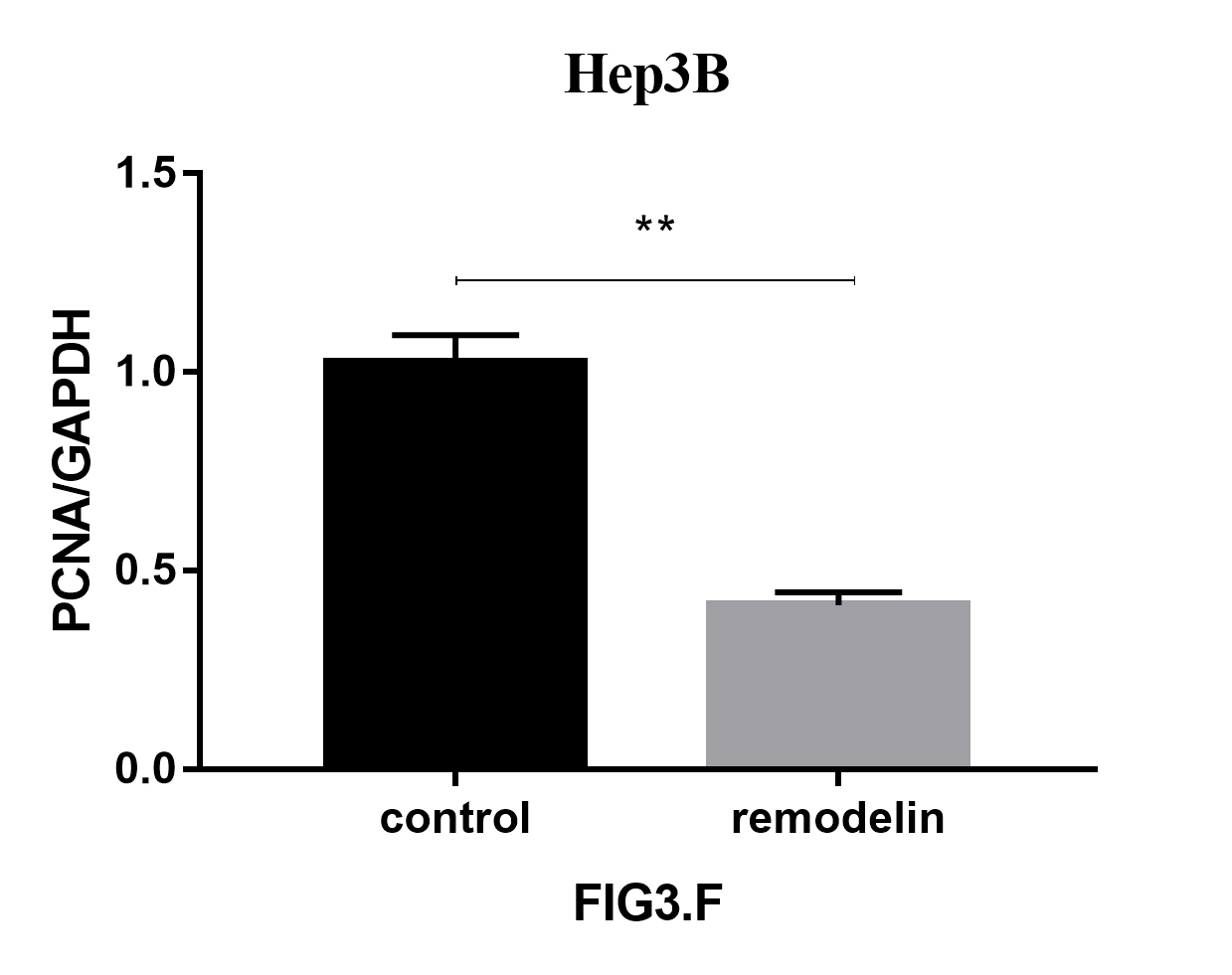


Fig 3F. hep3b-PCNA


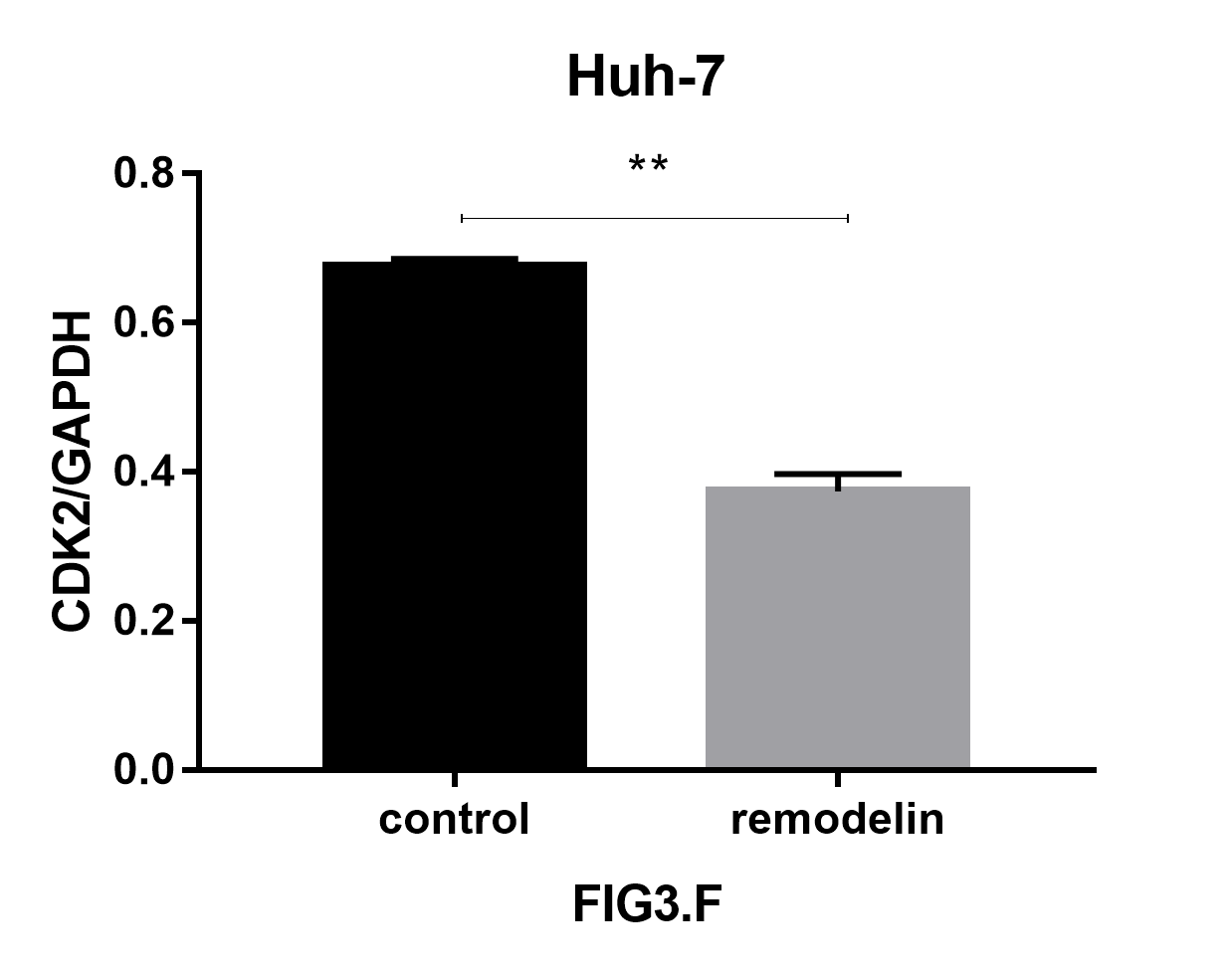


Fig 3F. huh-7-CDK2 (rem)


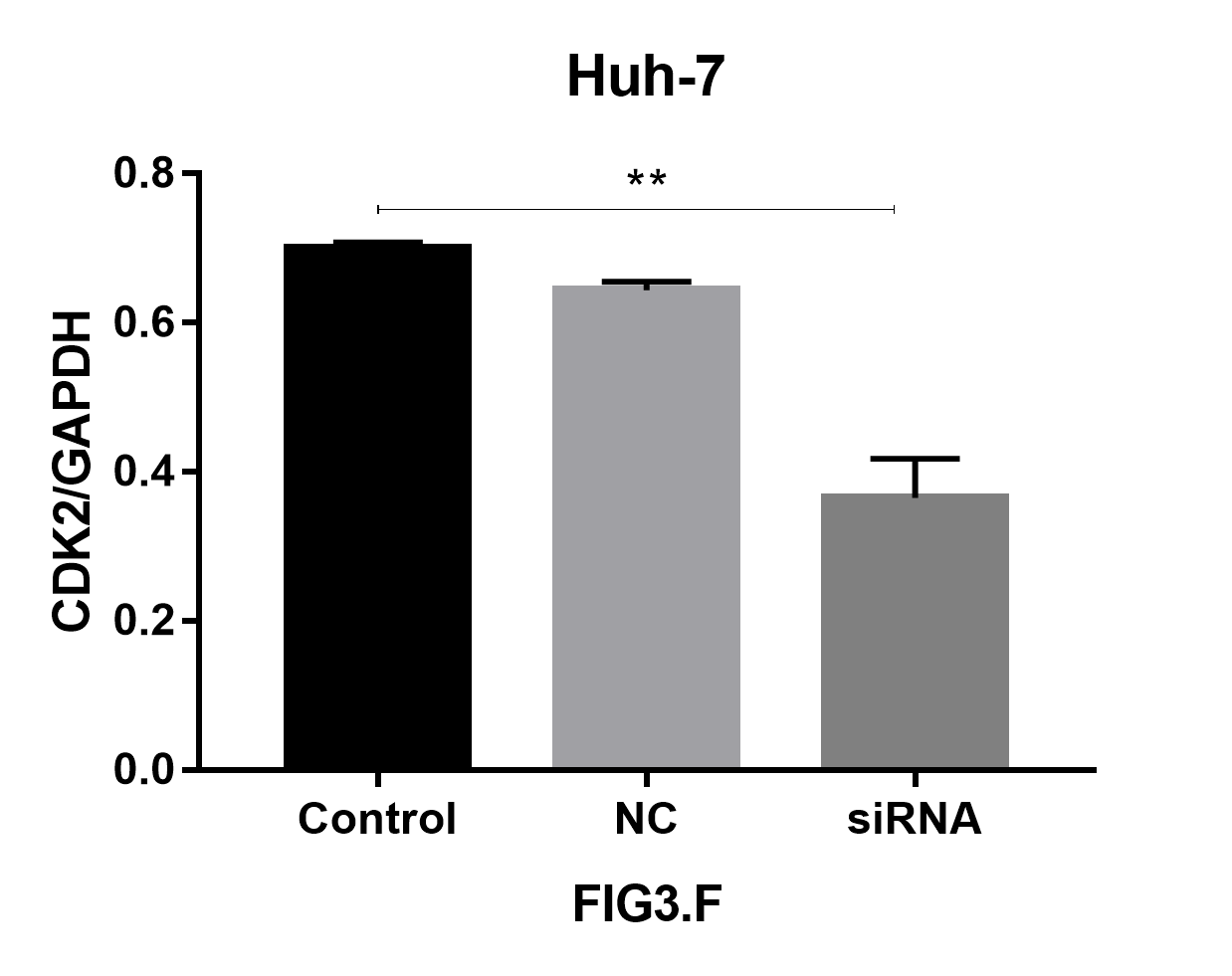


Fig 3F huh-7-CDK2


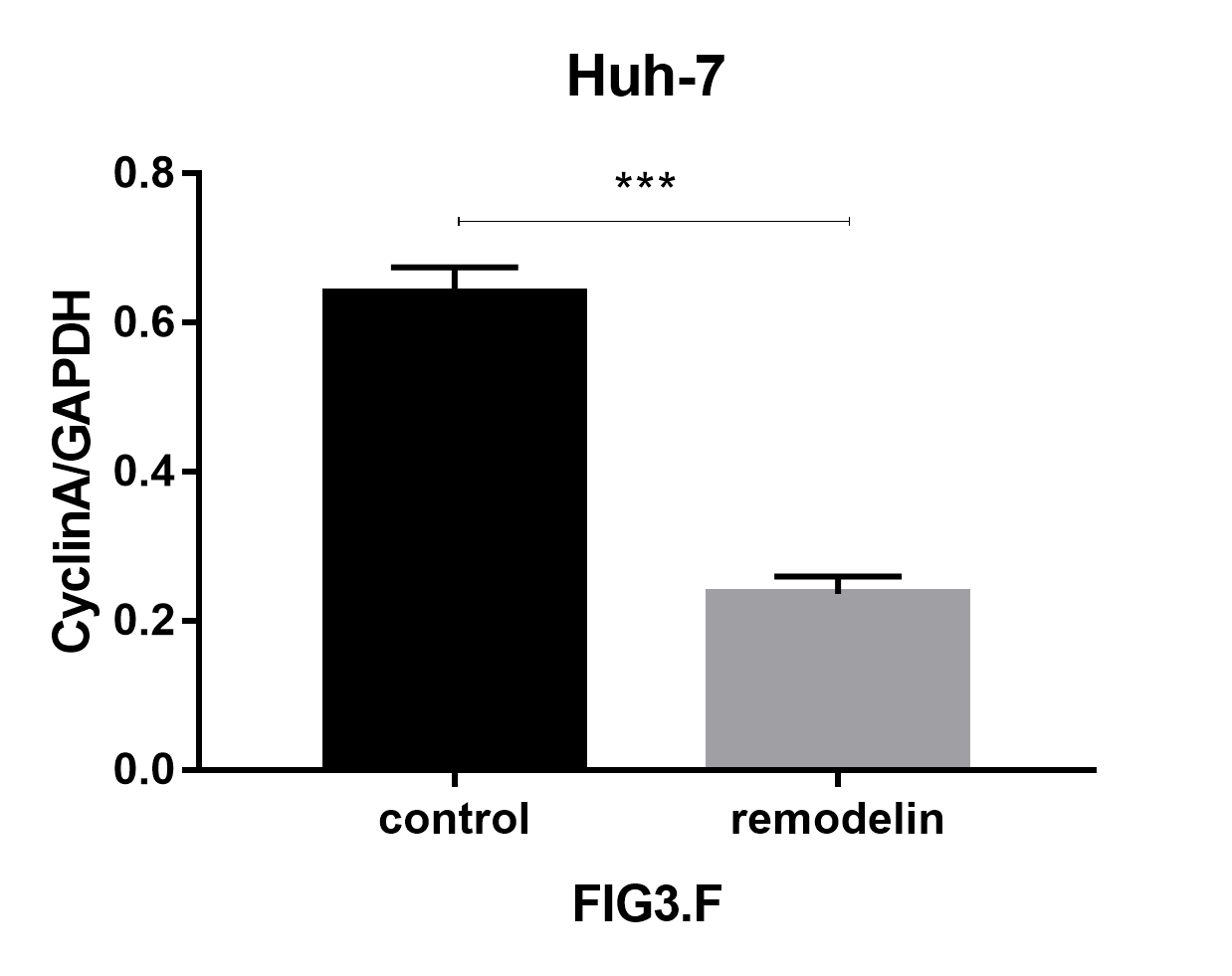


Fig 3F huh-7-CyclinA (rem)


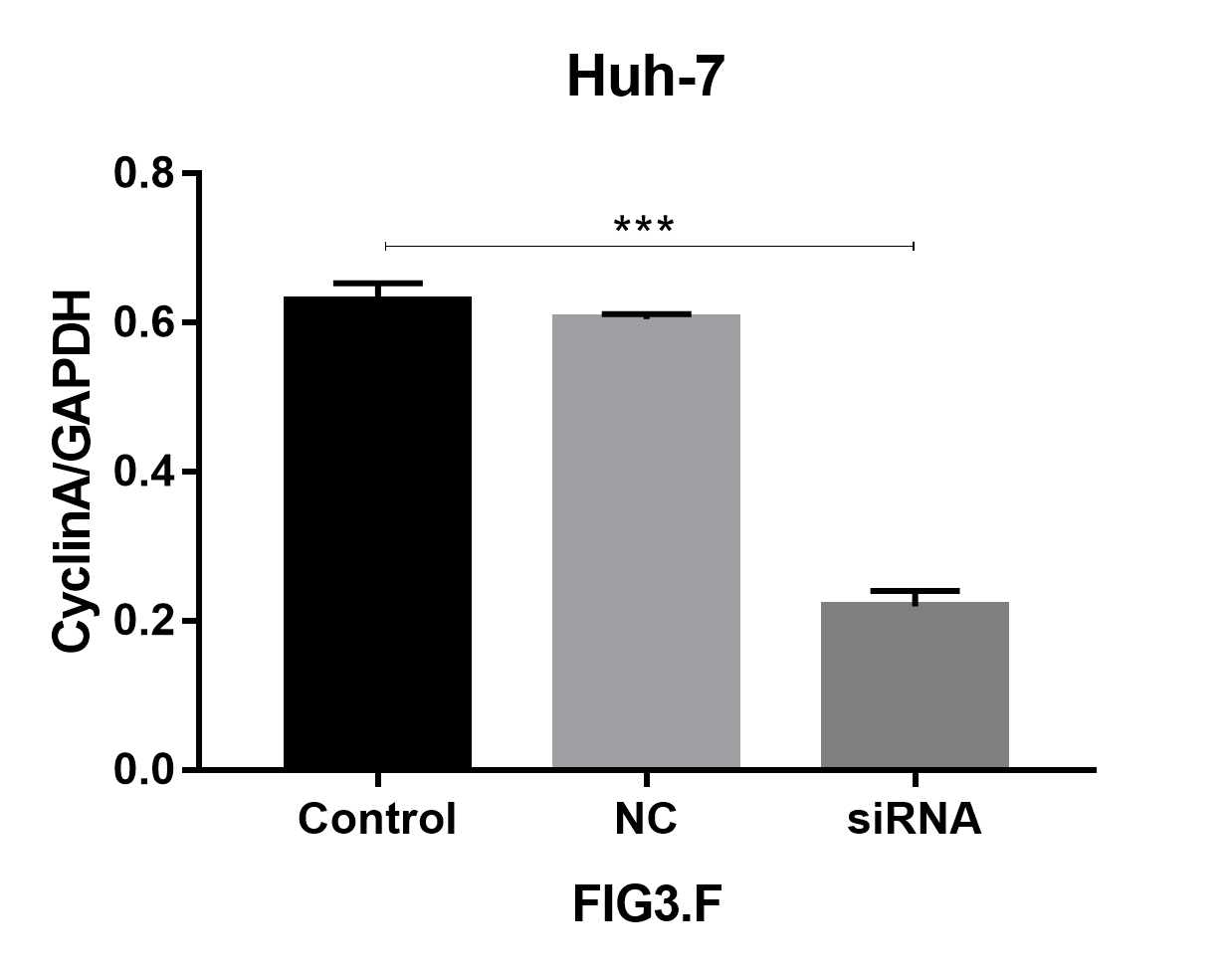


Fig 3F. huh-7-CyclinA


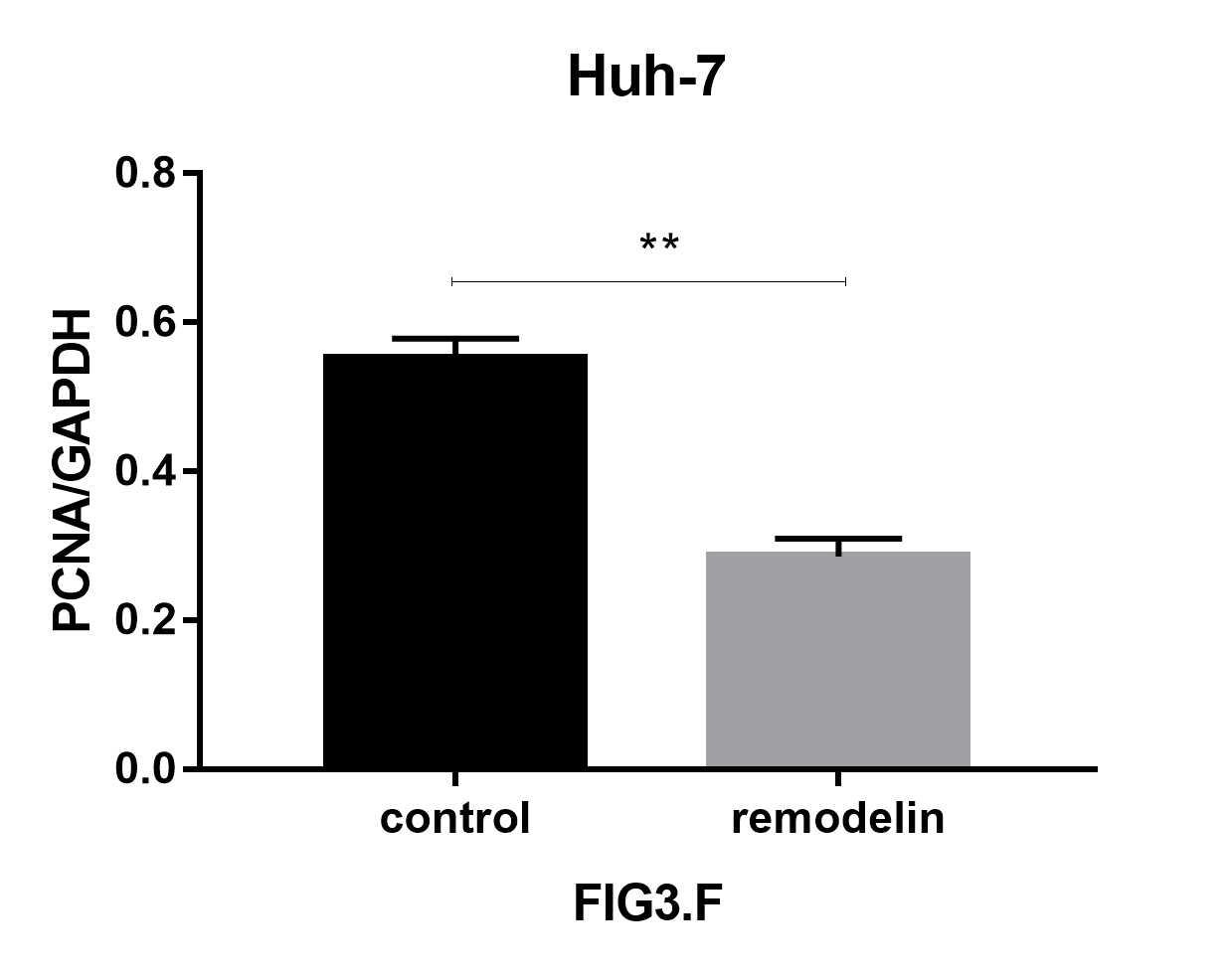


Fig 3F. huh-7-PCNA (rem)


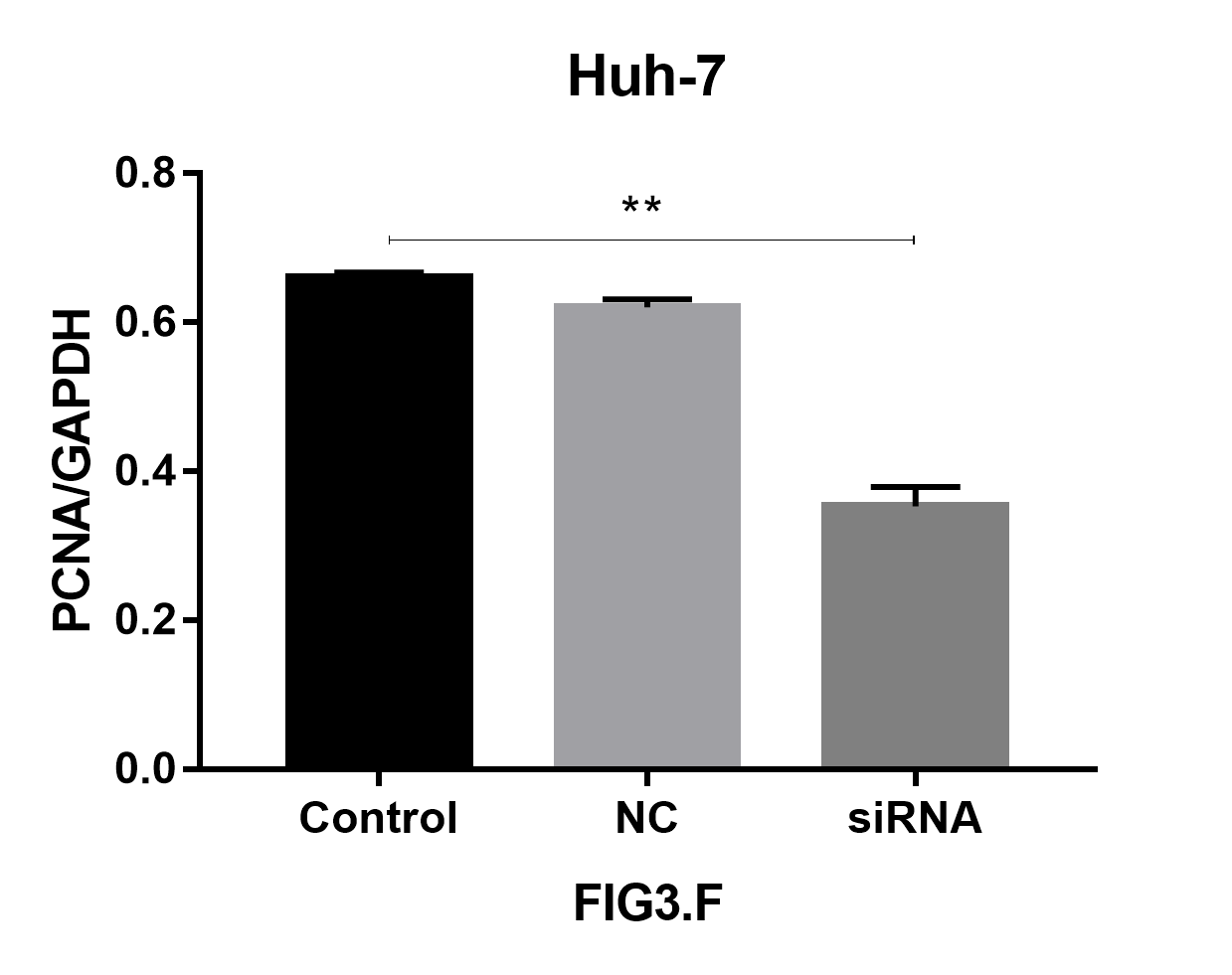


Fig 3F. huh-7-PCNA


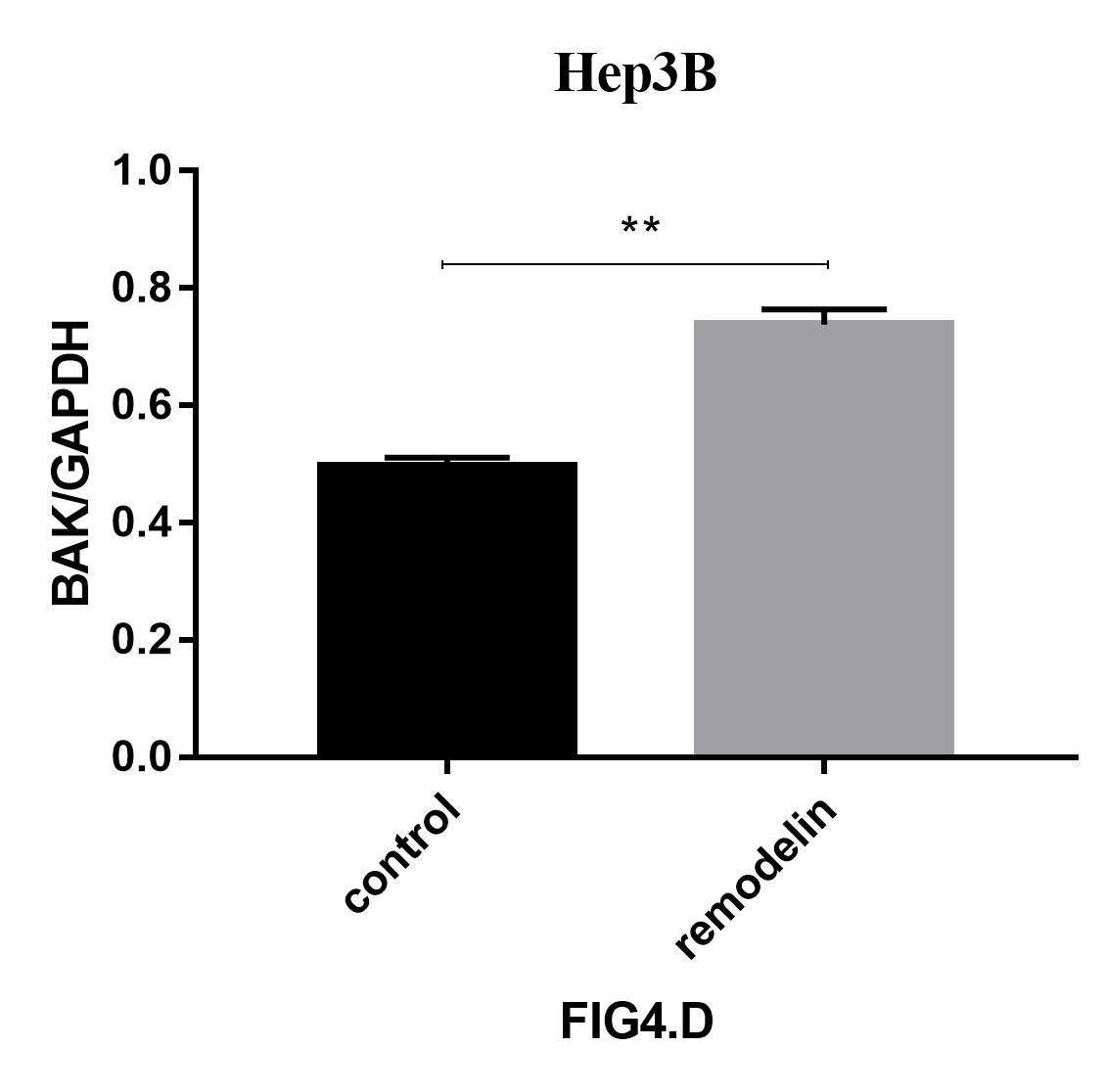


Fig 4D. hep3b-BAK


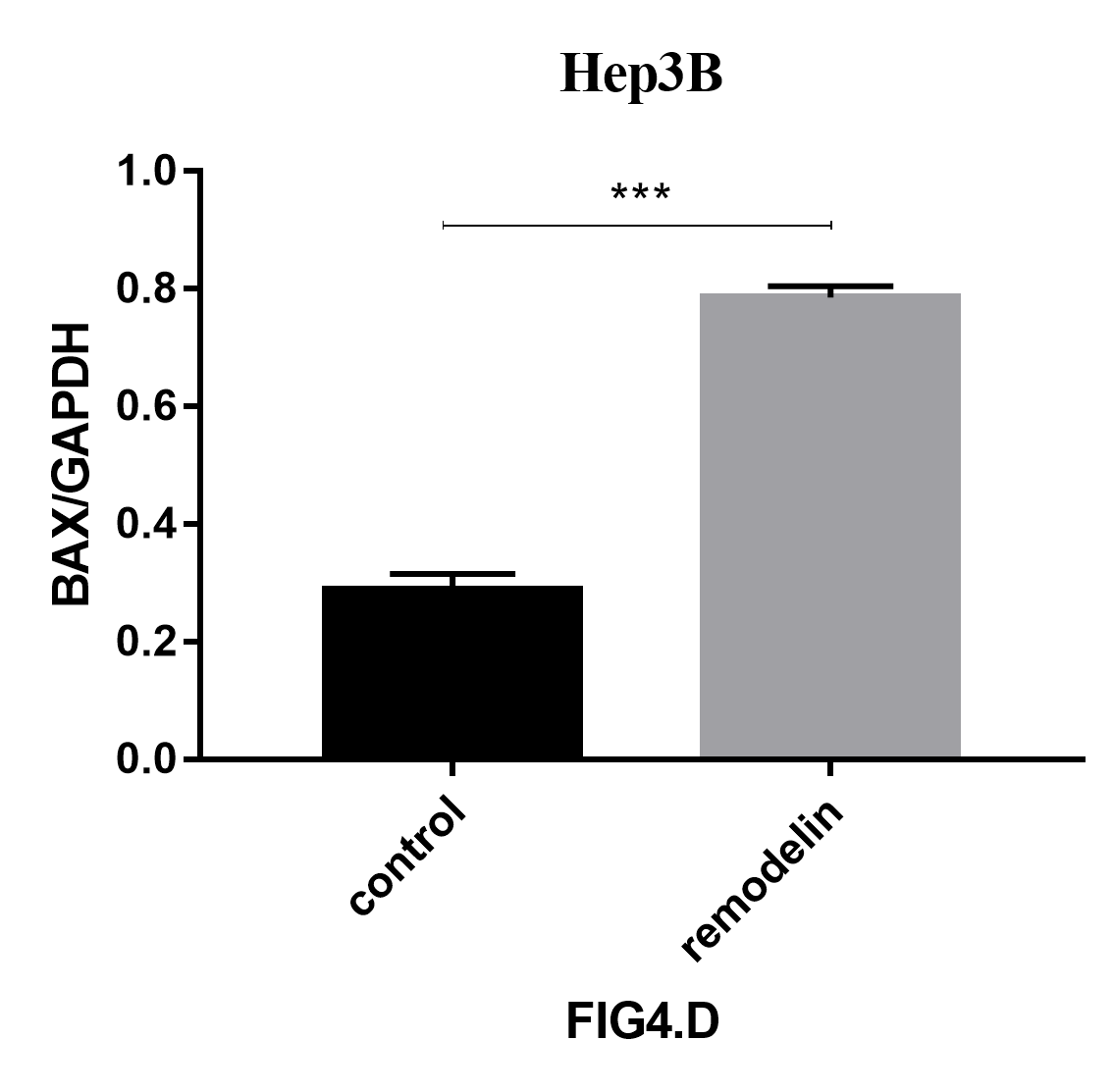


Fig 4D. hep3b-BAX


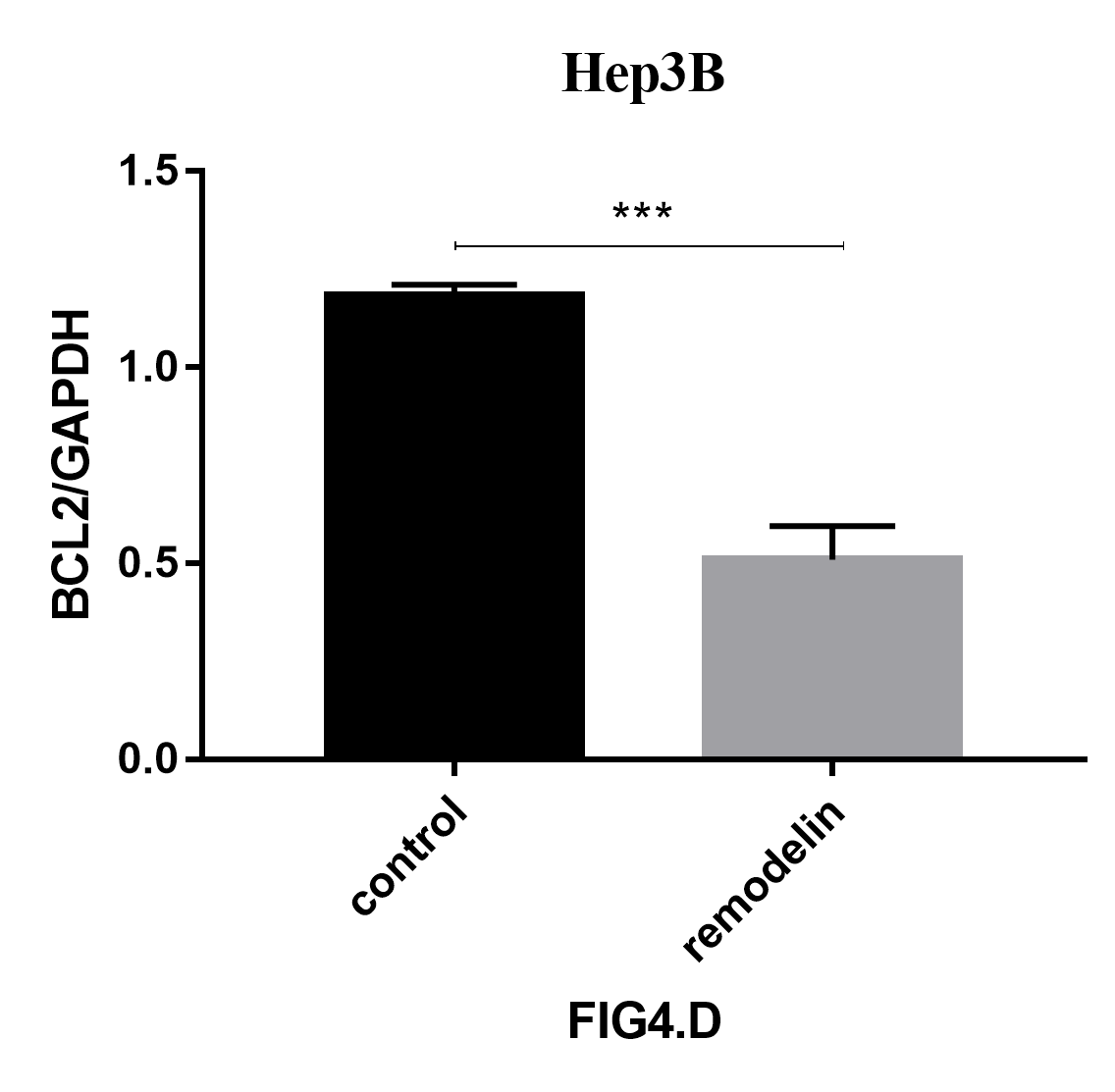


Fig 4D. hep3b-BCL2


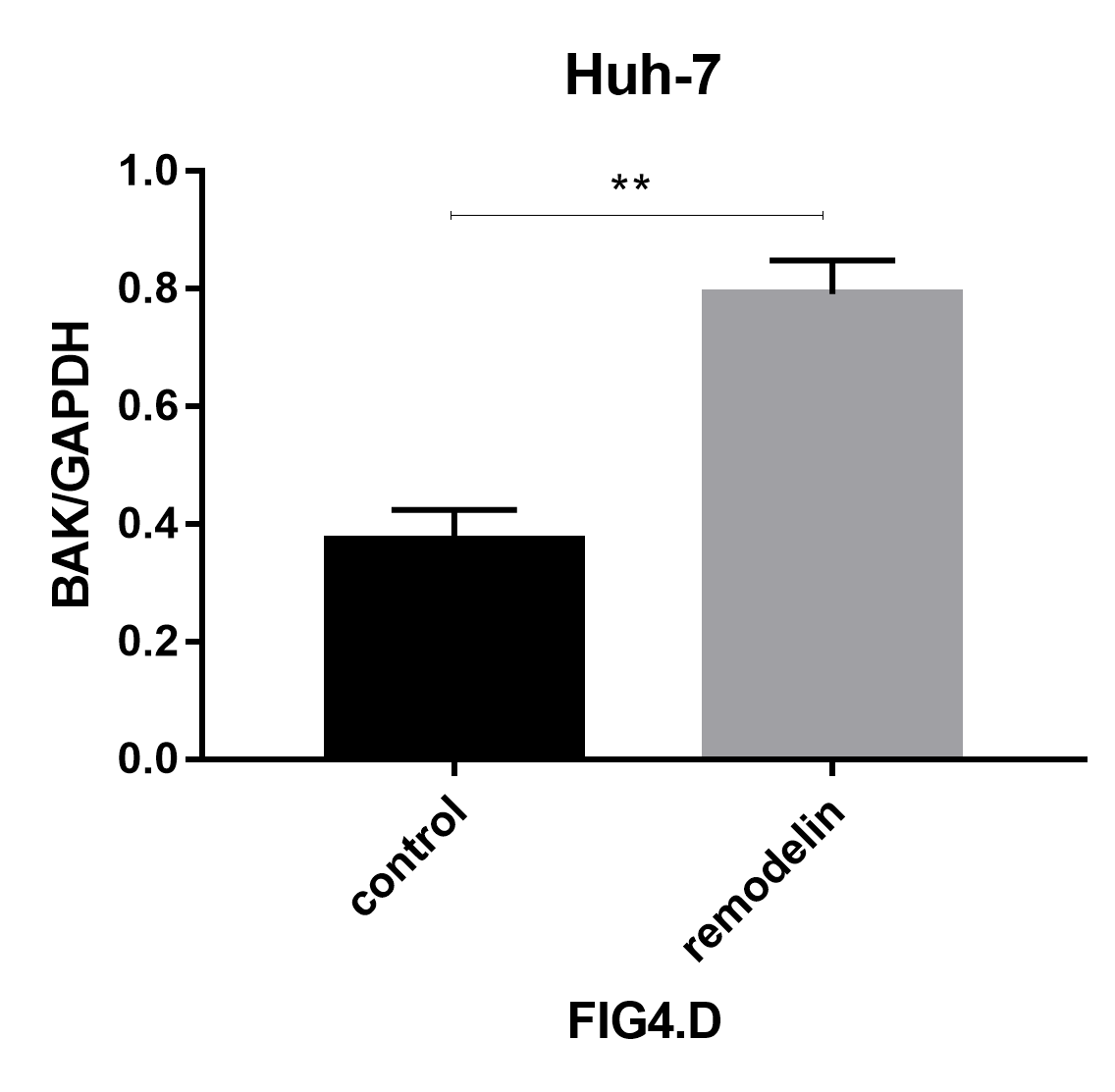


Fig 4D. huh-7-BAK (rem）


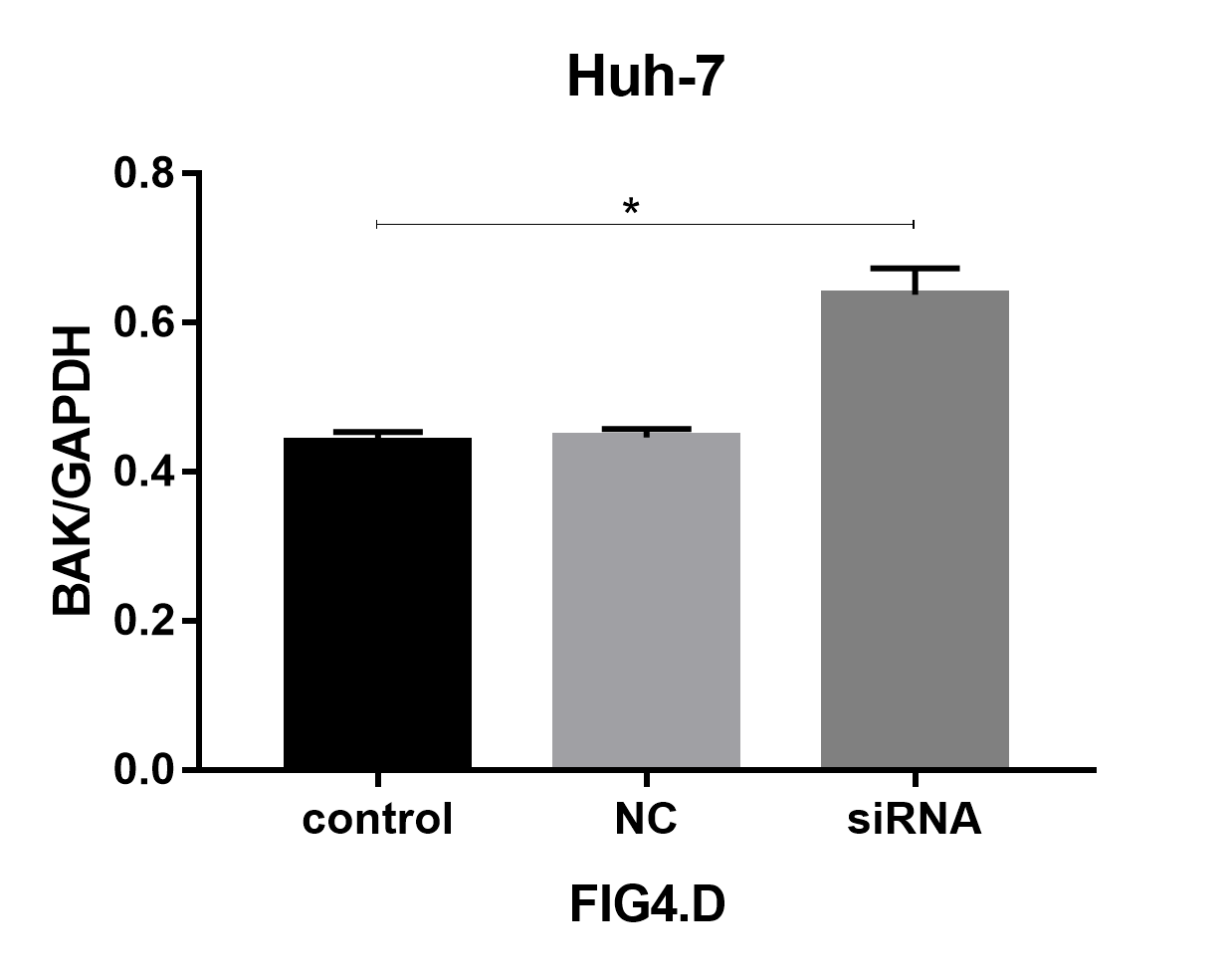


Fig 4D. huh-7-BAK


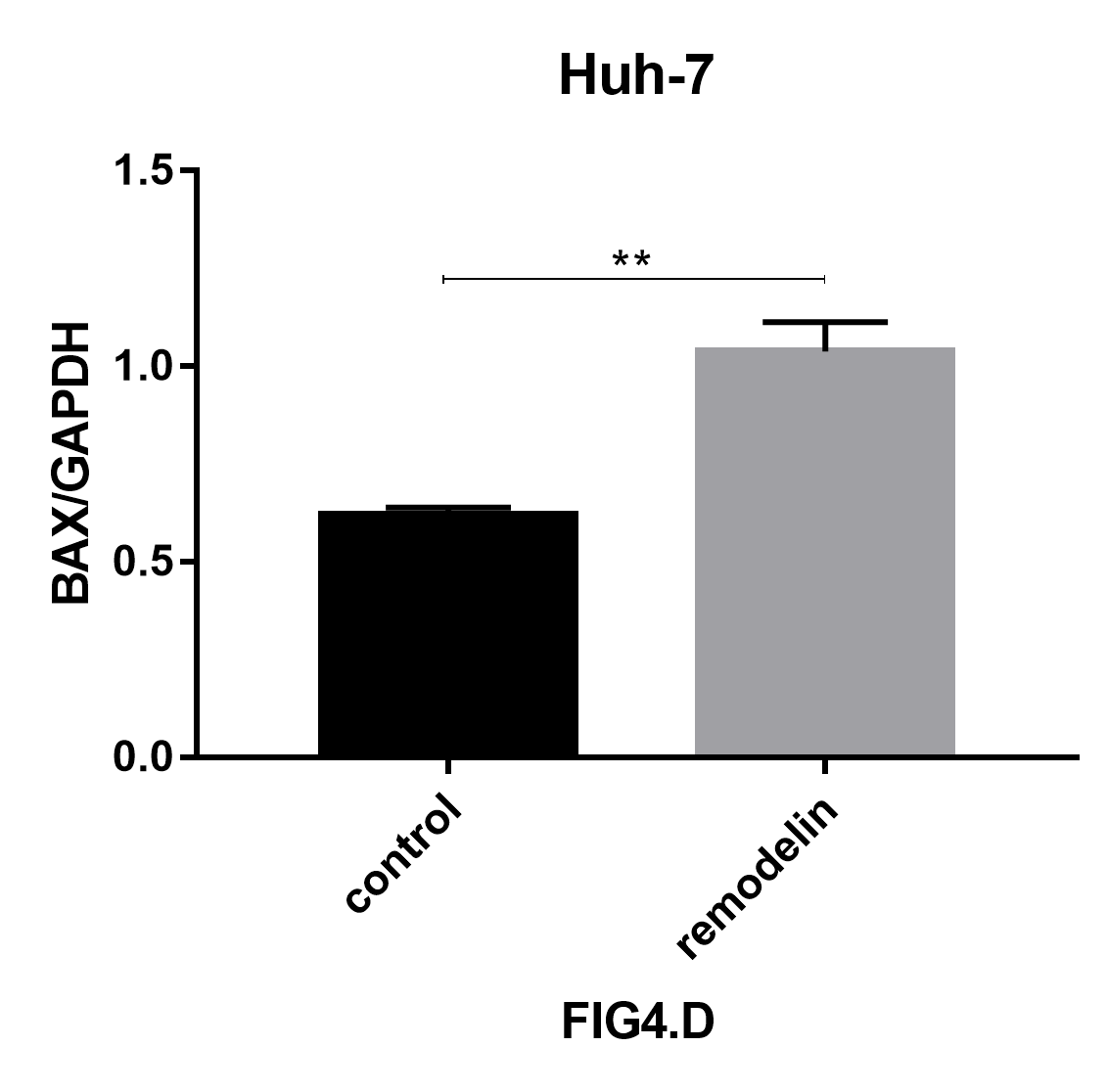


Fig 4D. huh-7-BAX (rem)


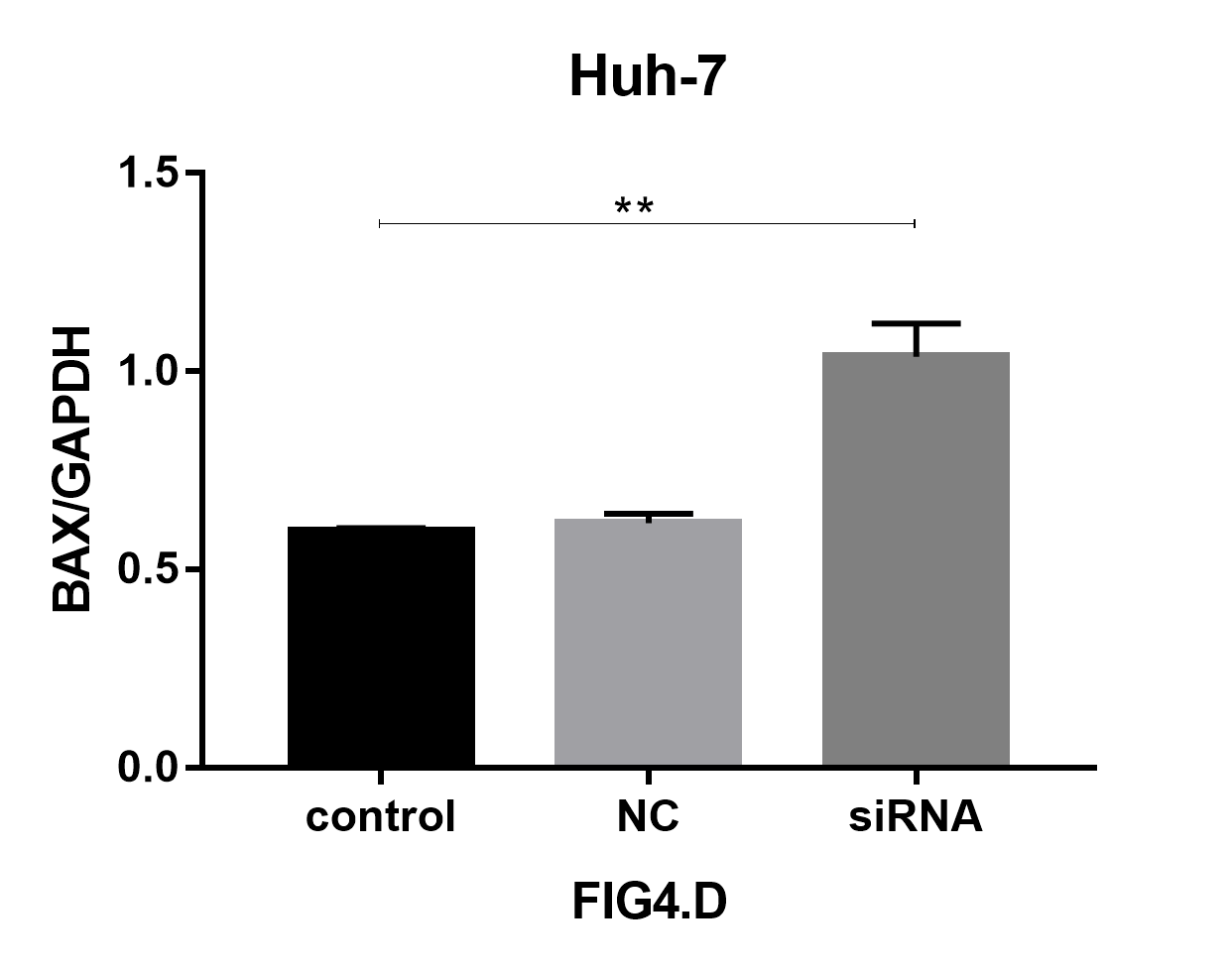


Fig 4D. huh-7-BAX


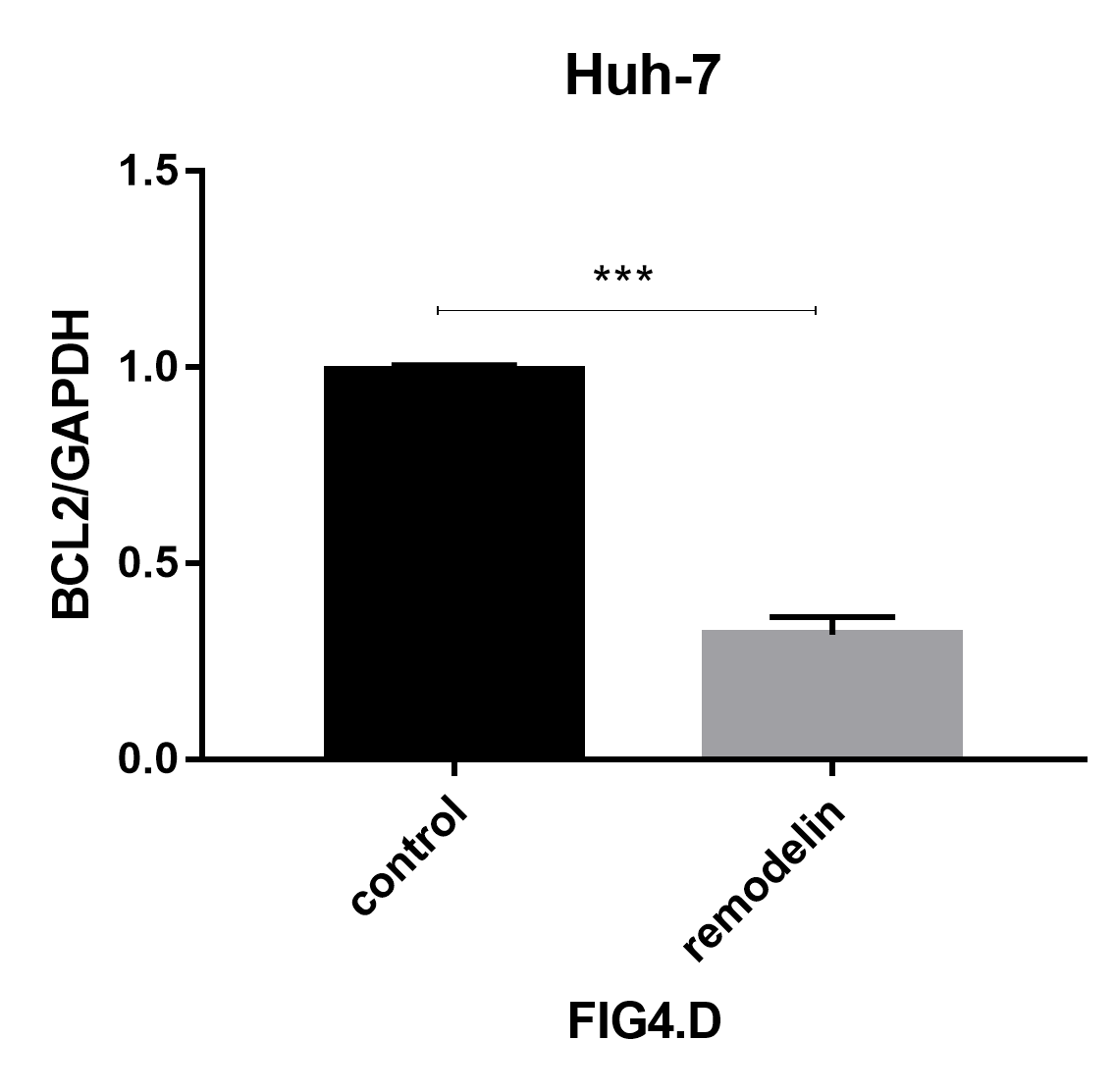


Fig 4D. huh-7-BCL2 (rem)


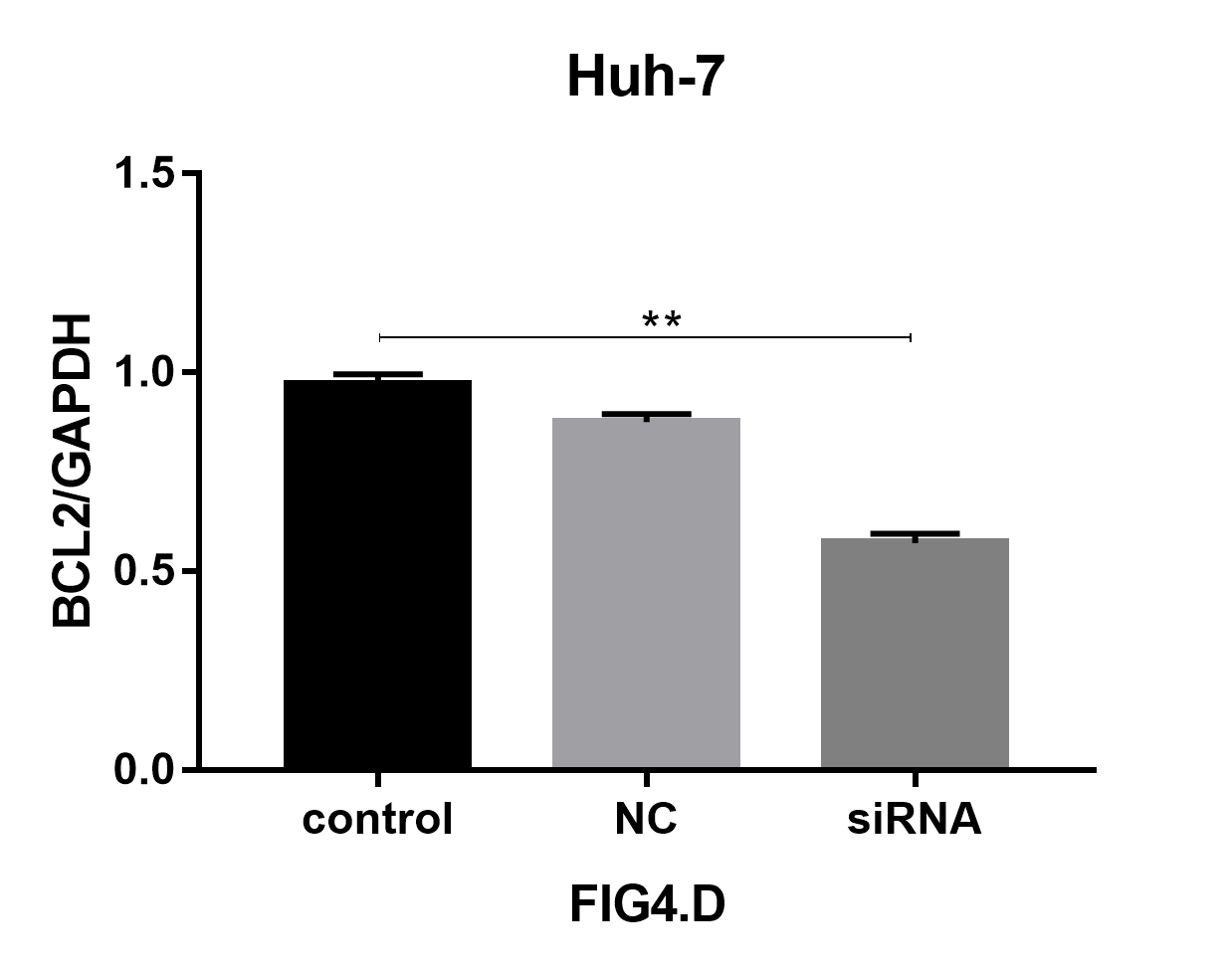


Fig 4D. huh-7-BCL2


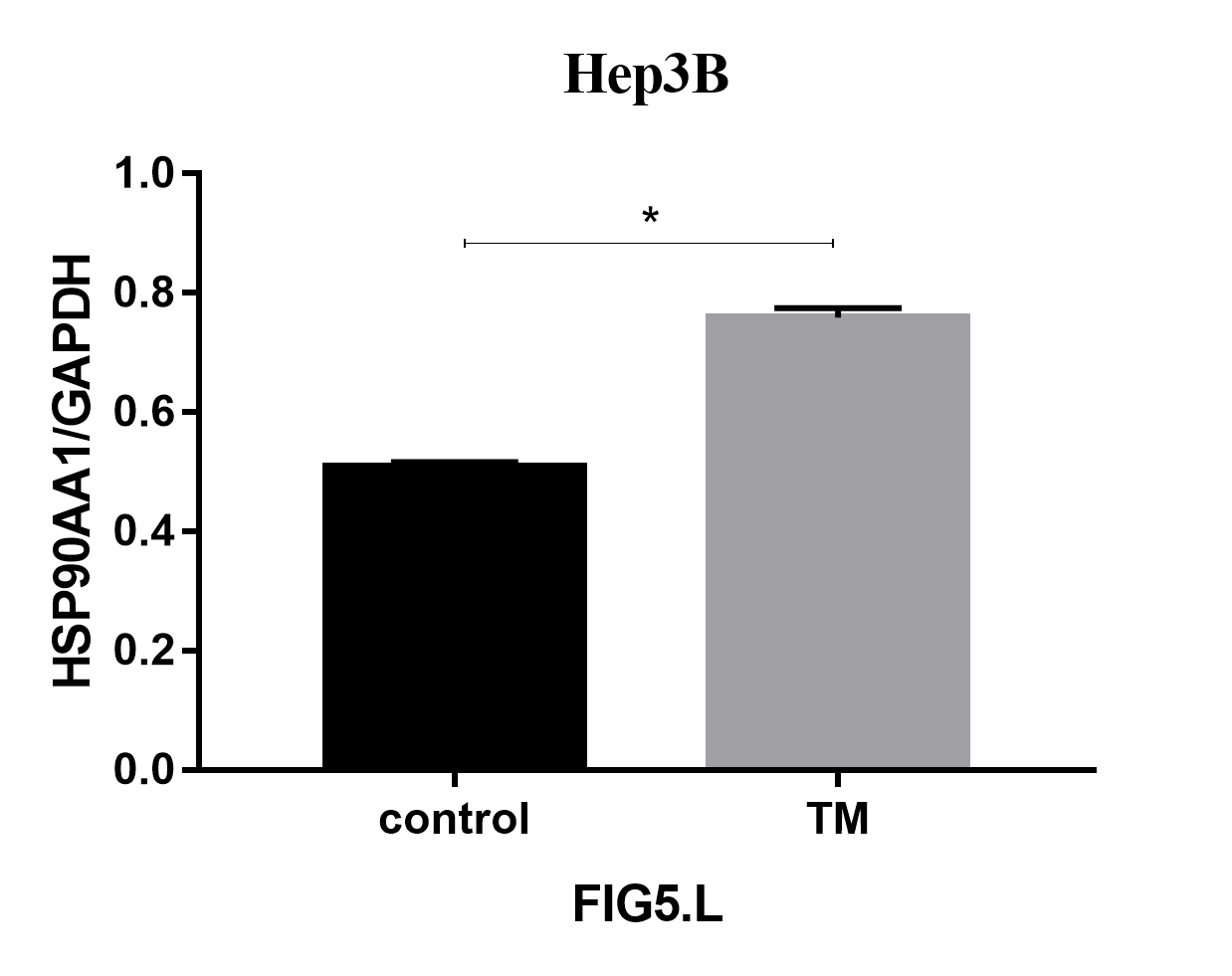


Fig 5L. hep3b-HSP90AA1


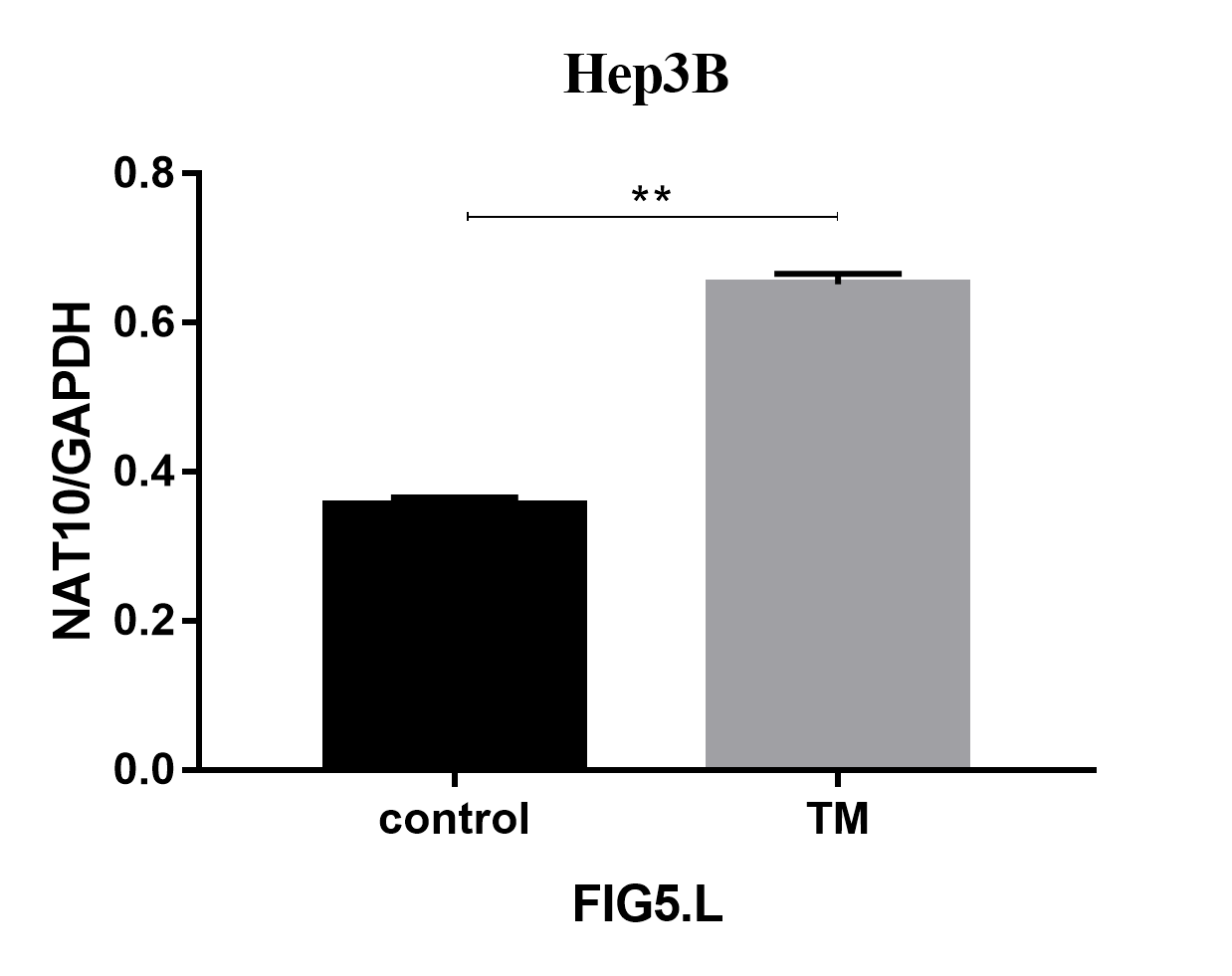


Fig 5L. hep3b-NAT10


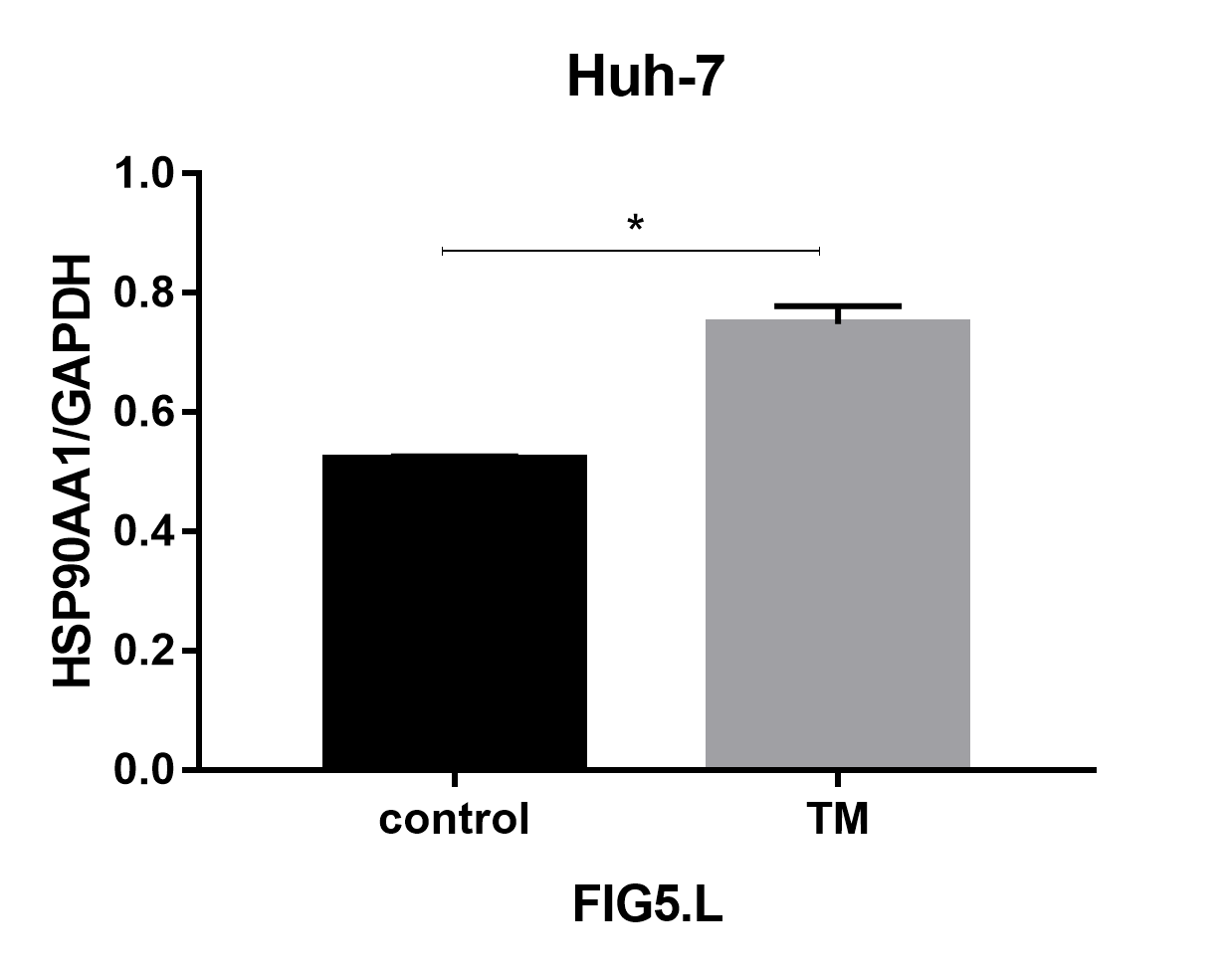


Fig 5L. huh-7-HSP90AA1


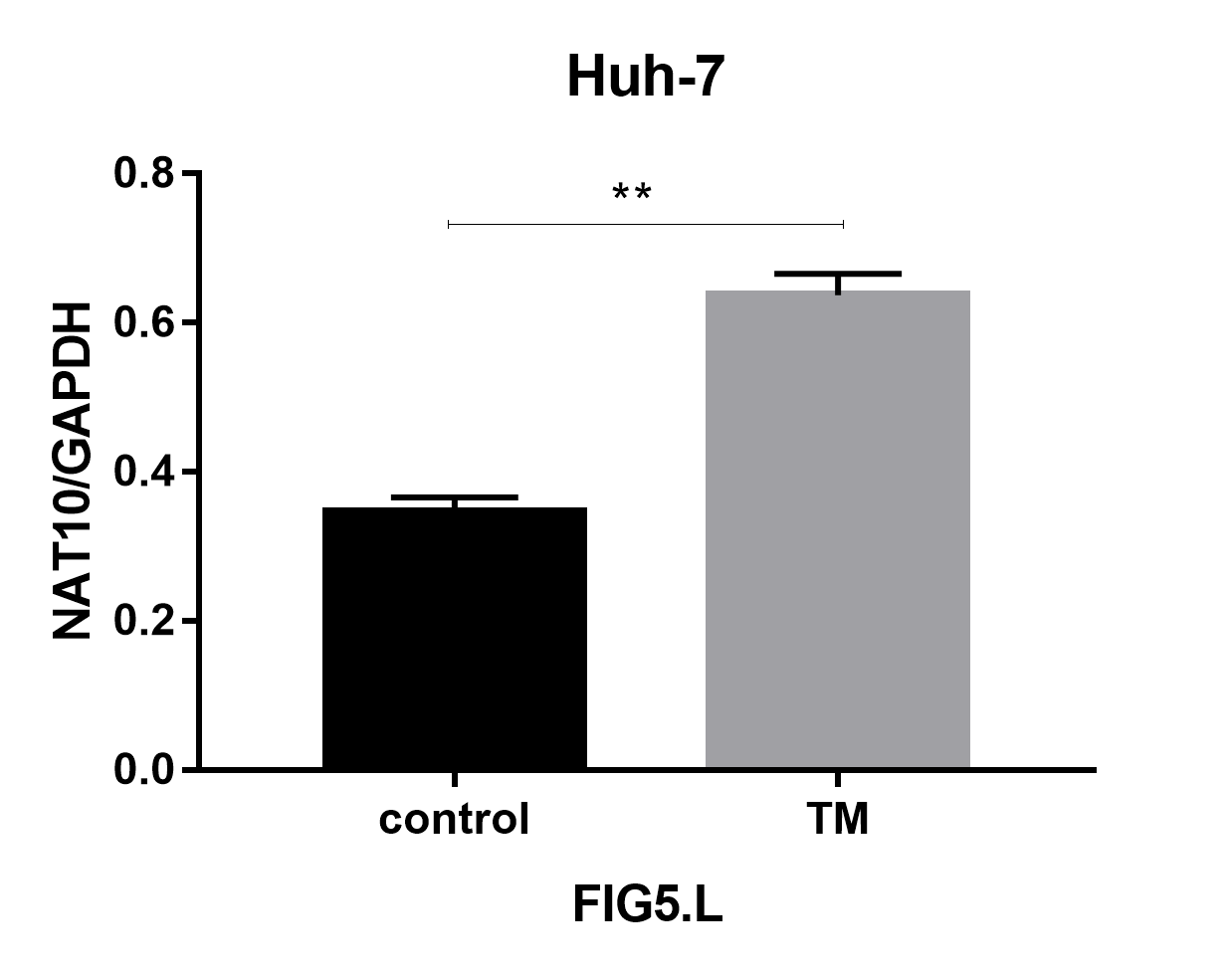


Fig 5L. huh-7-NAT10


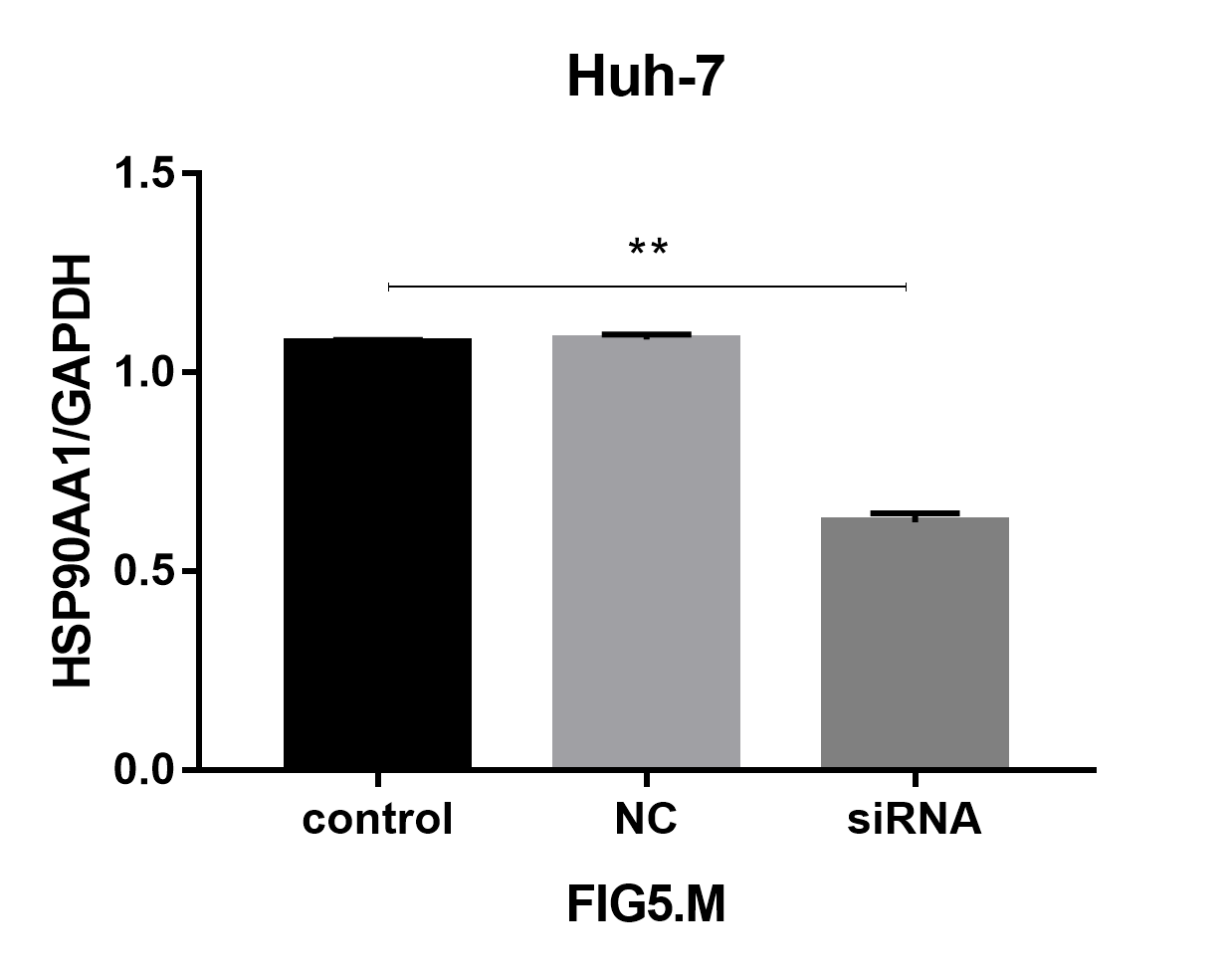


Fig 5M. huh-7-HSP90AA1


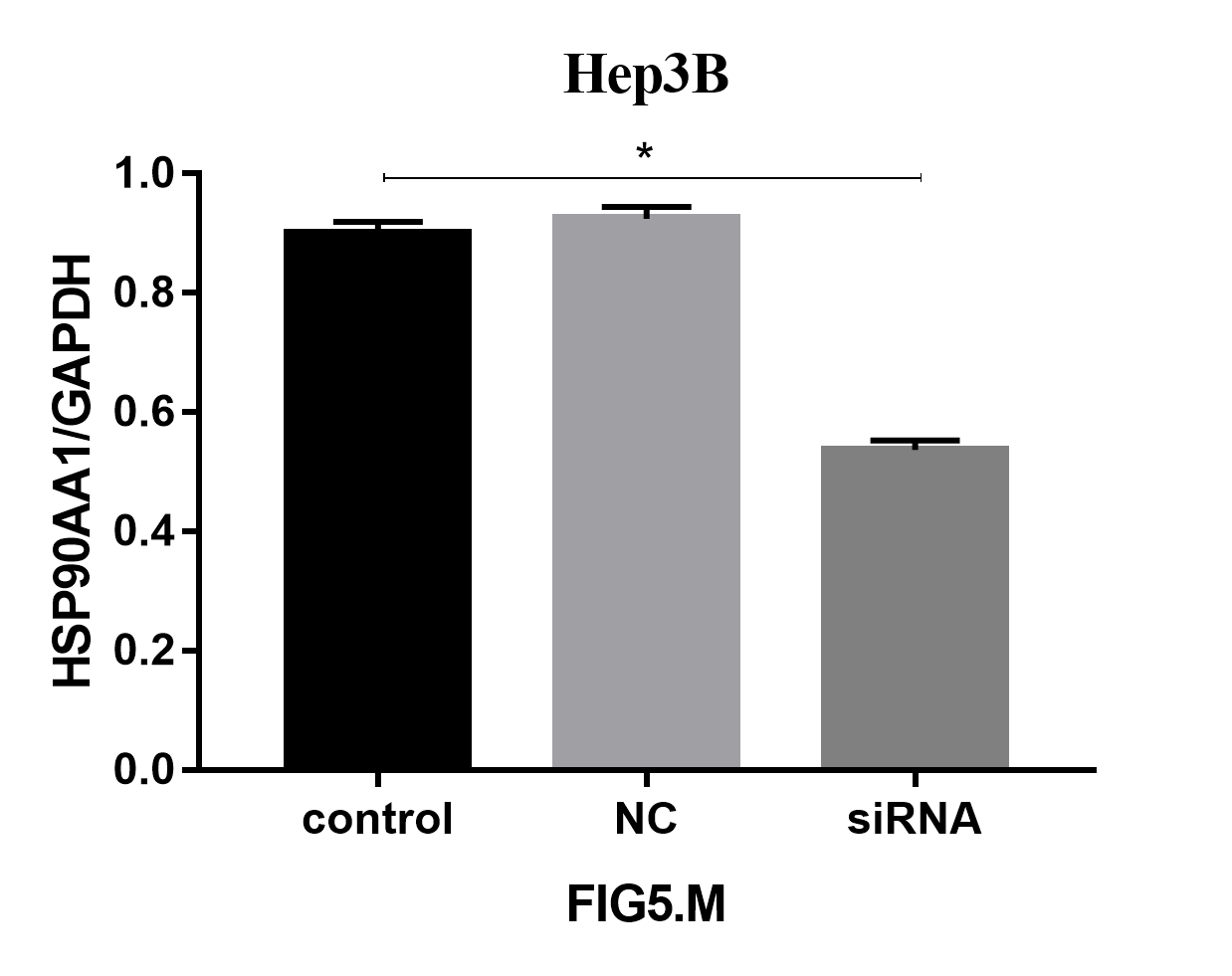


Fig 5M. hep3b-HSP90AA1


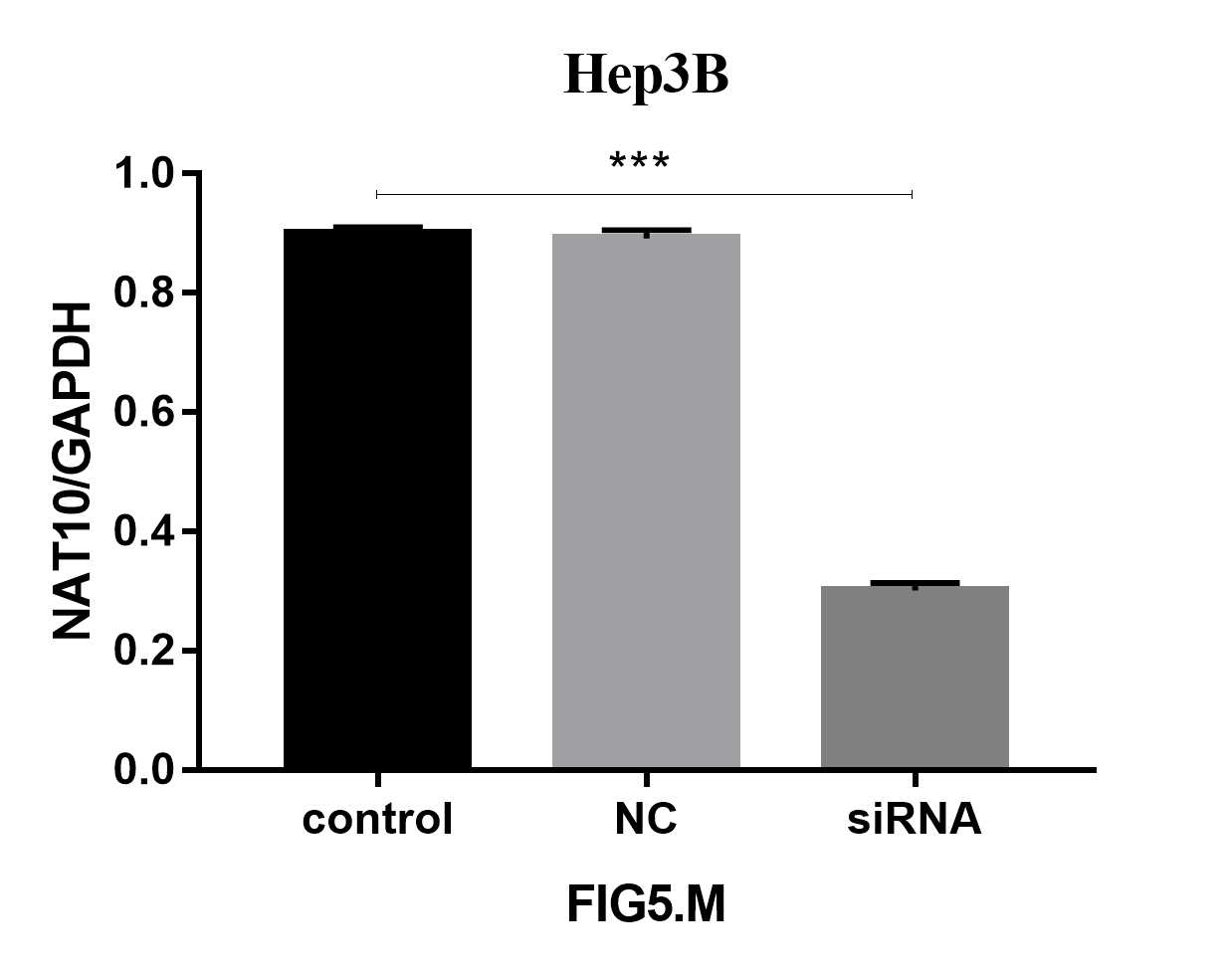


Fig 5M. hep3b-NAT10


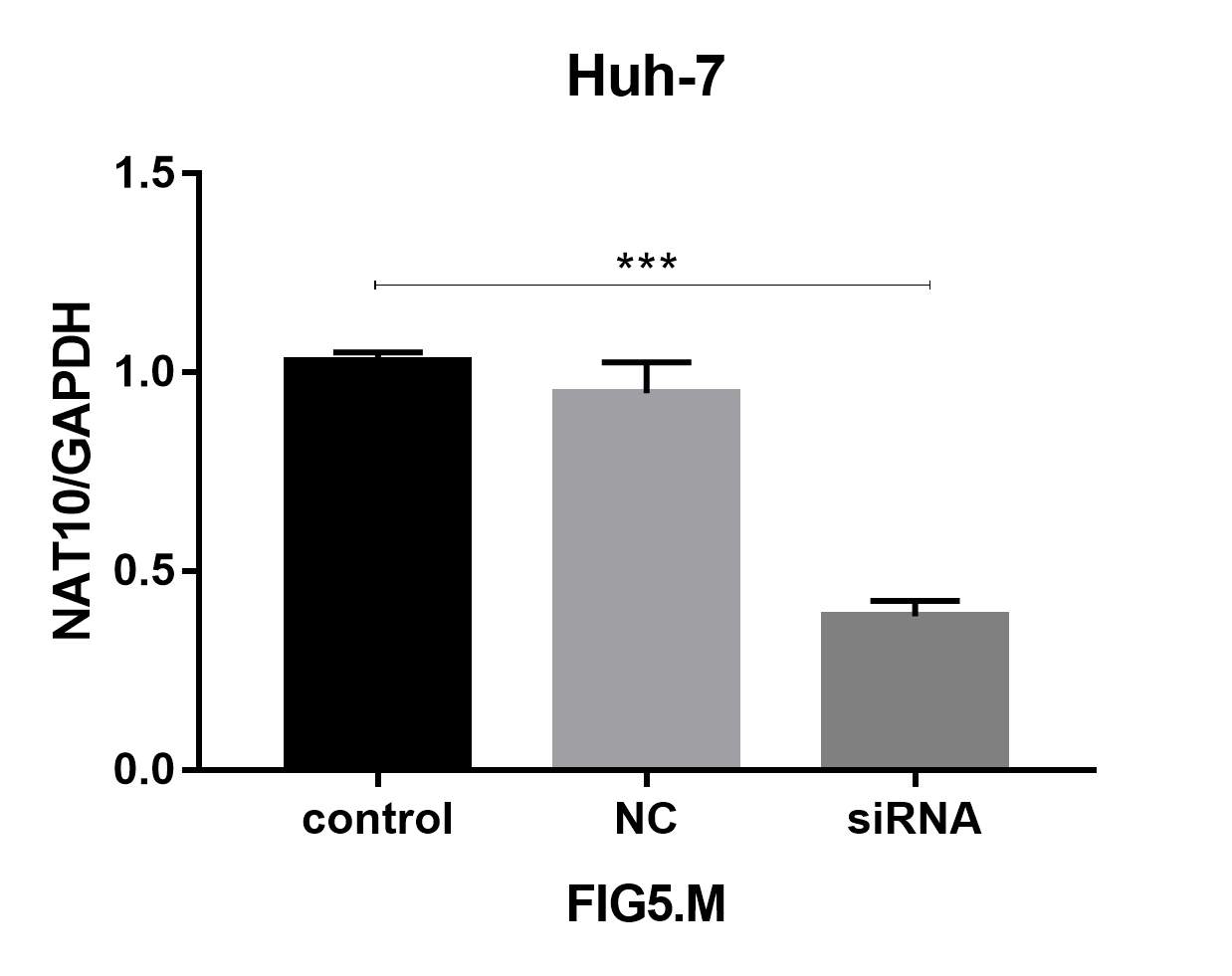


Fig 5M. huh-7-NAT10


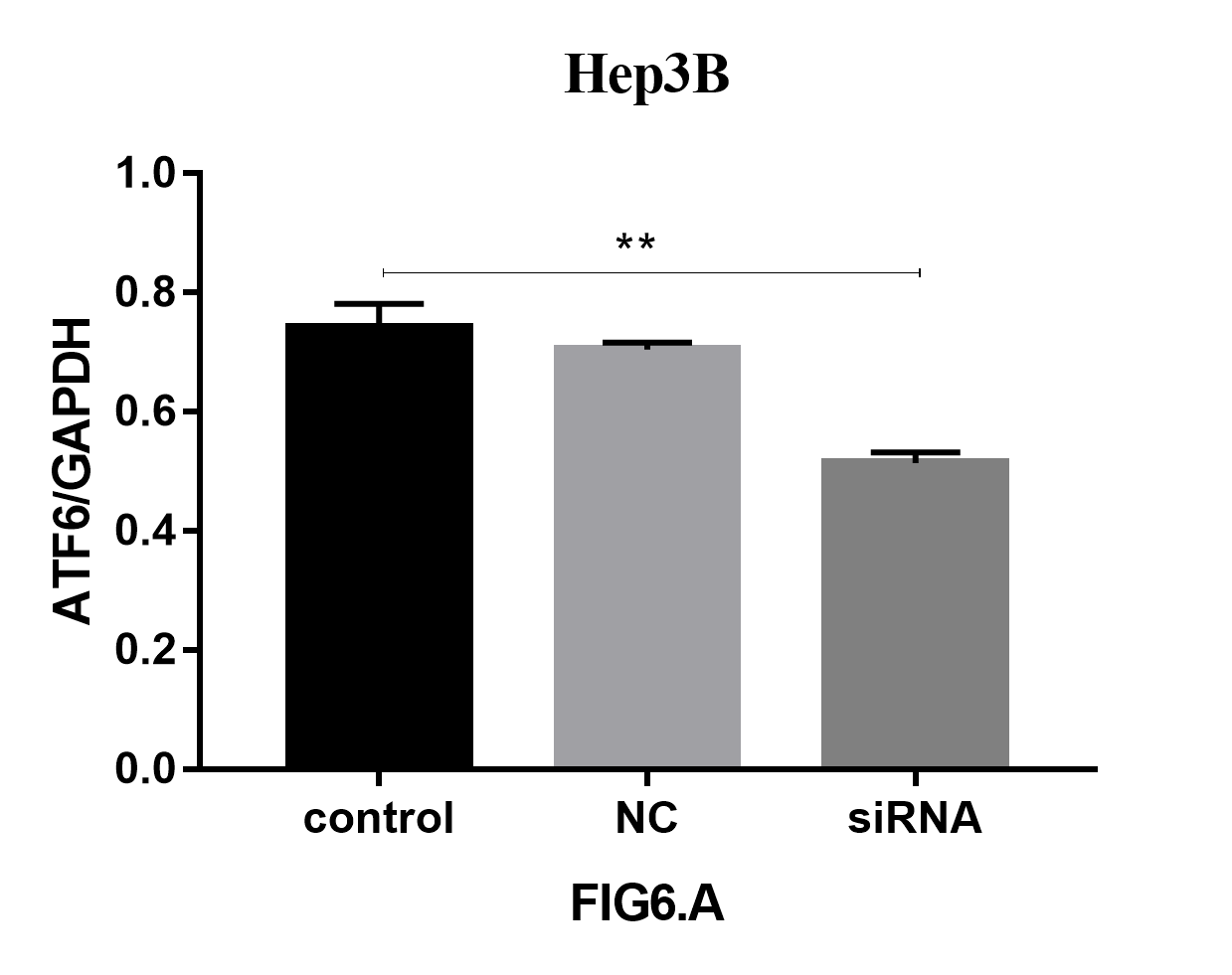


Fig 6A. hep3b-ATF6


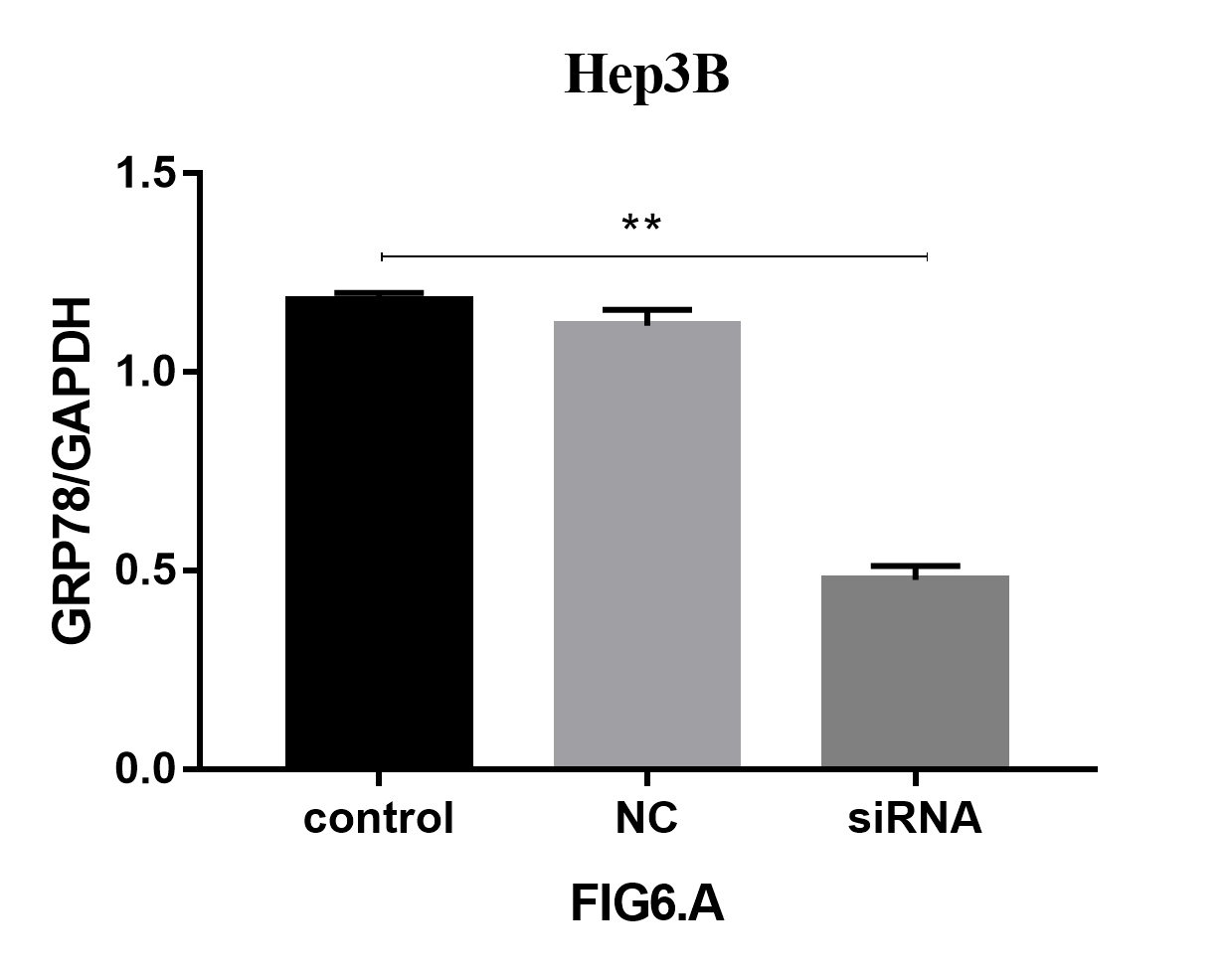


Fig 6A. hep3b-GRP78


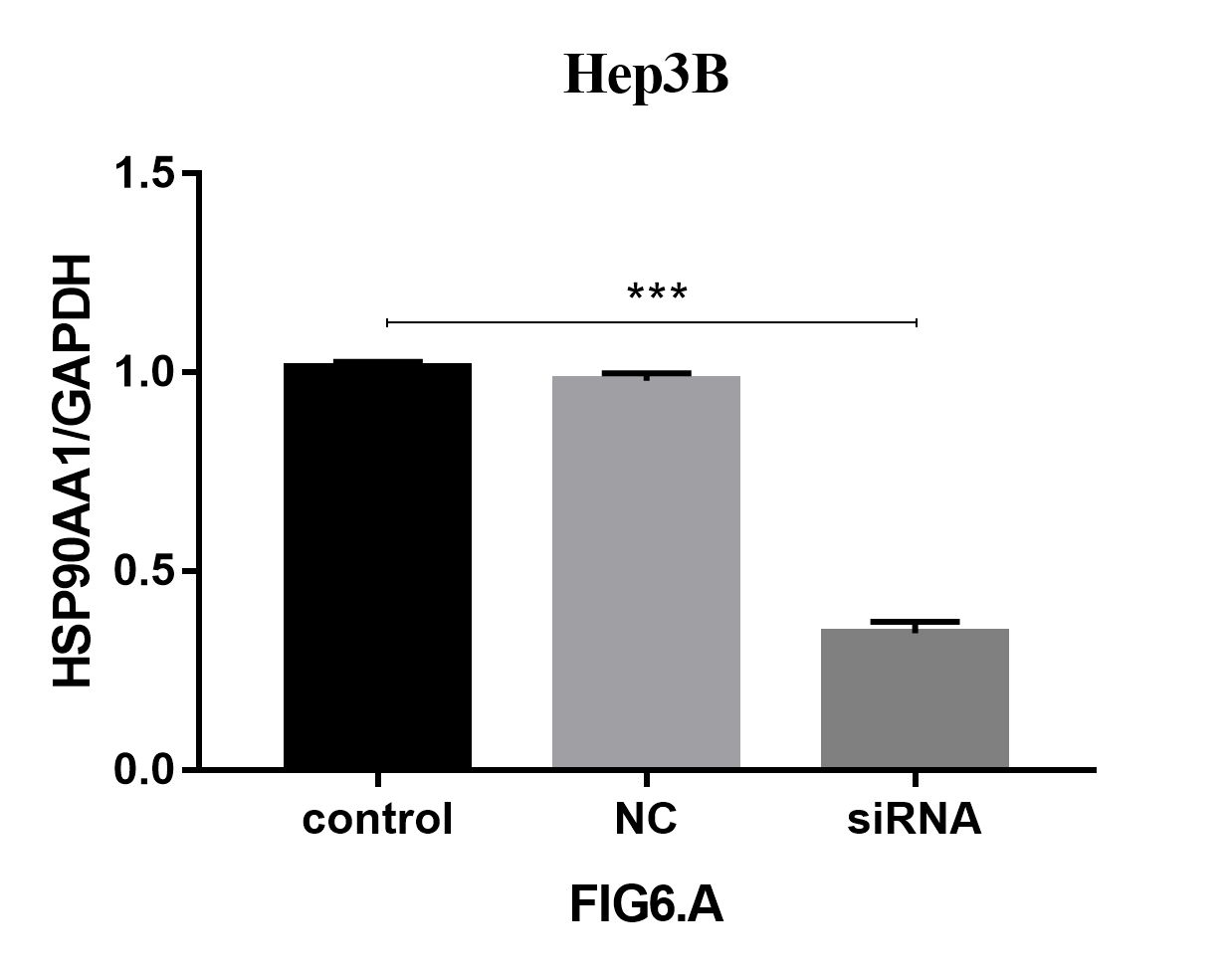


Fig 6A. hep3b-HSP90AA1


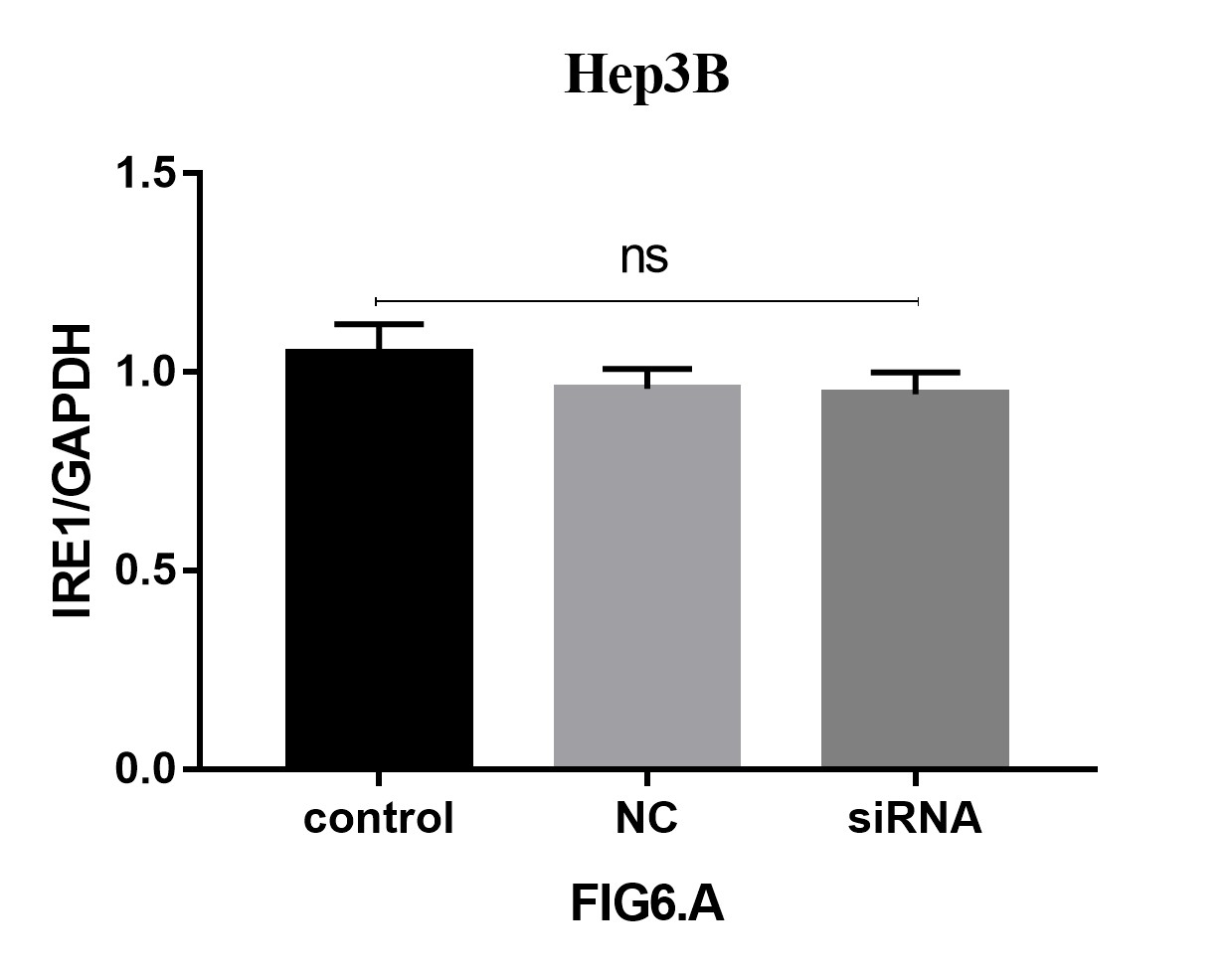


Fig 6A. hep3b-IRE-1


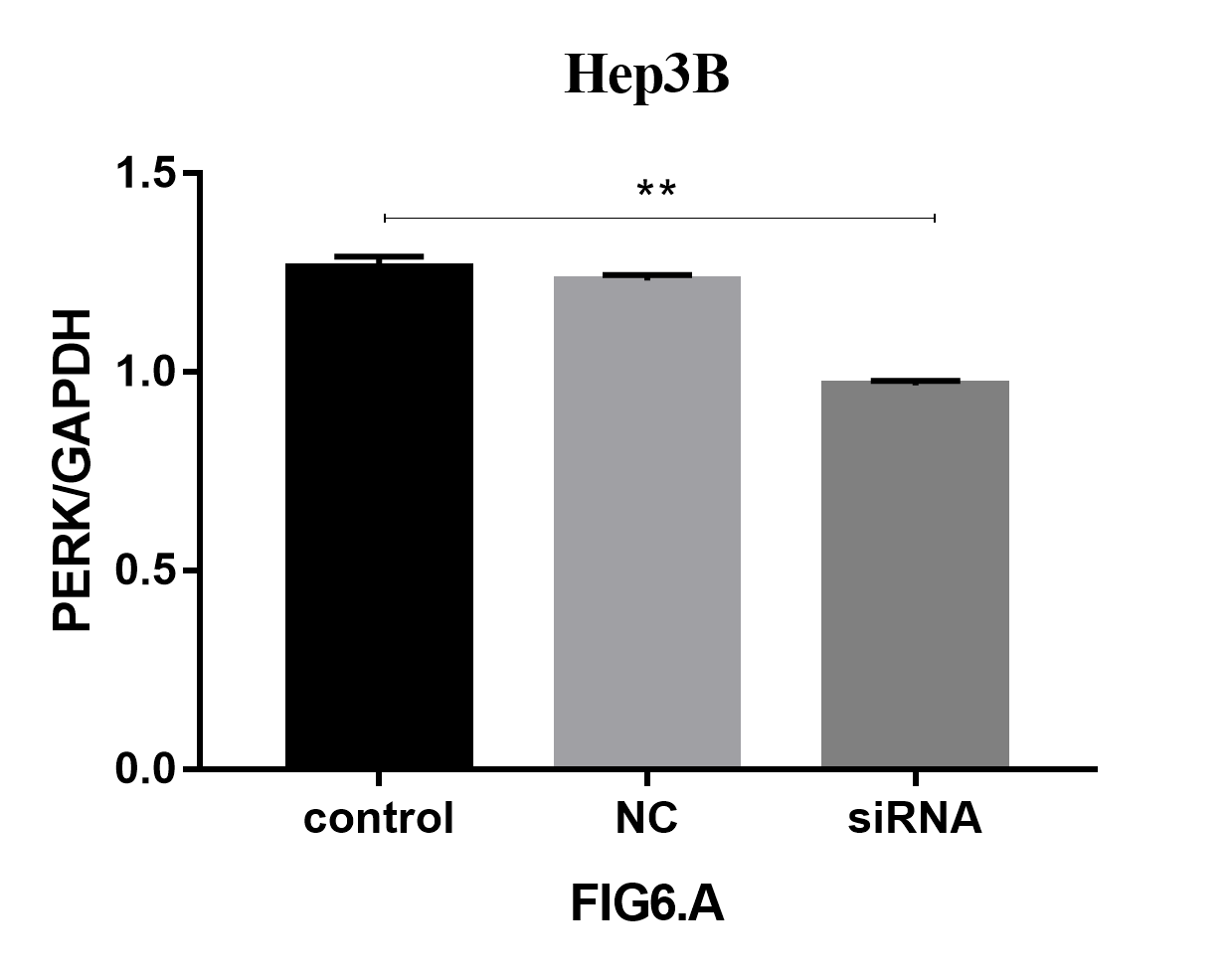


Fig 6A. hep3b-PERK


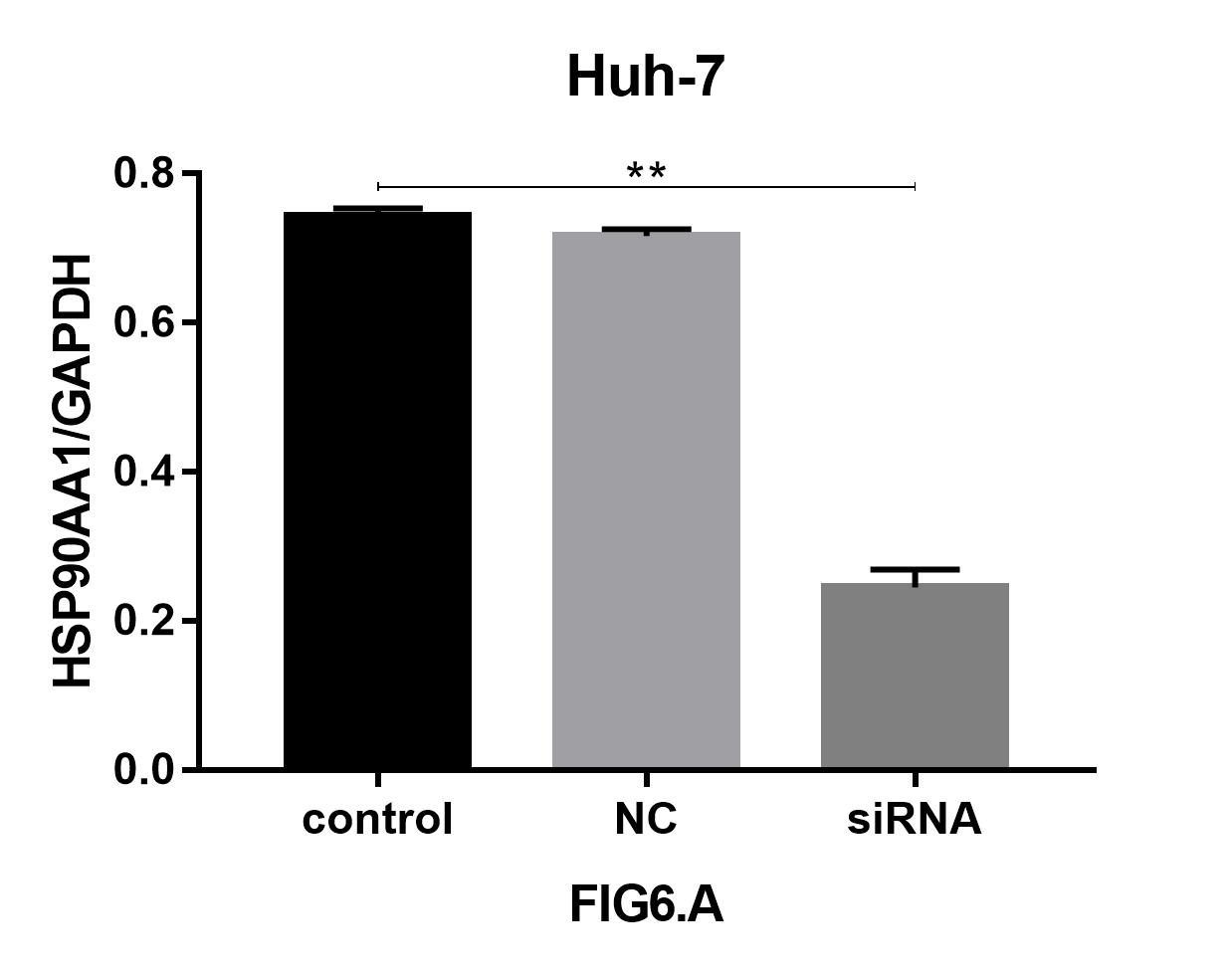


Fig 6A. huh-7-HSP90AA1


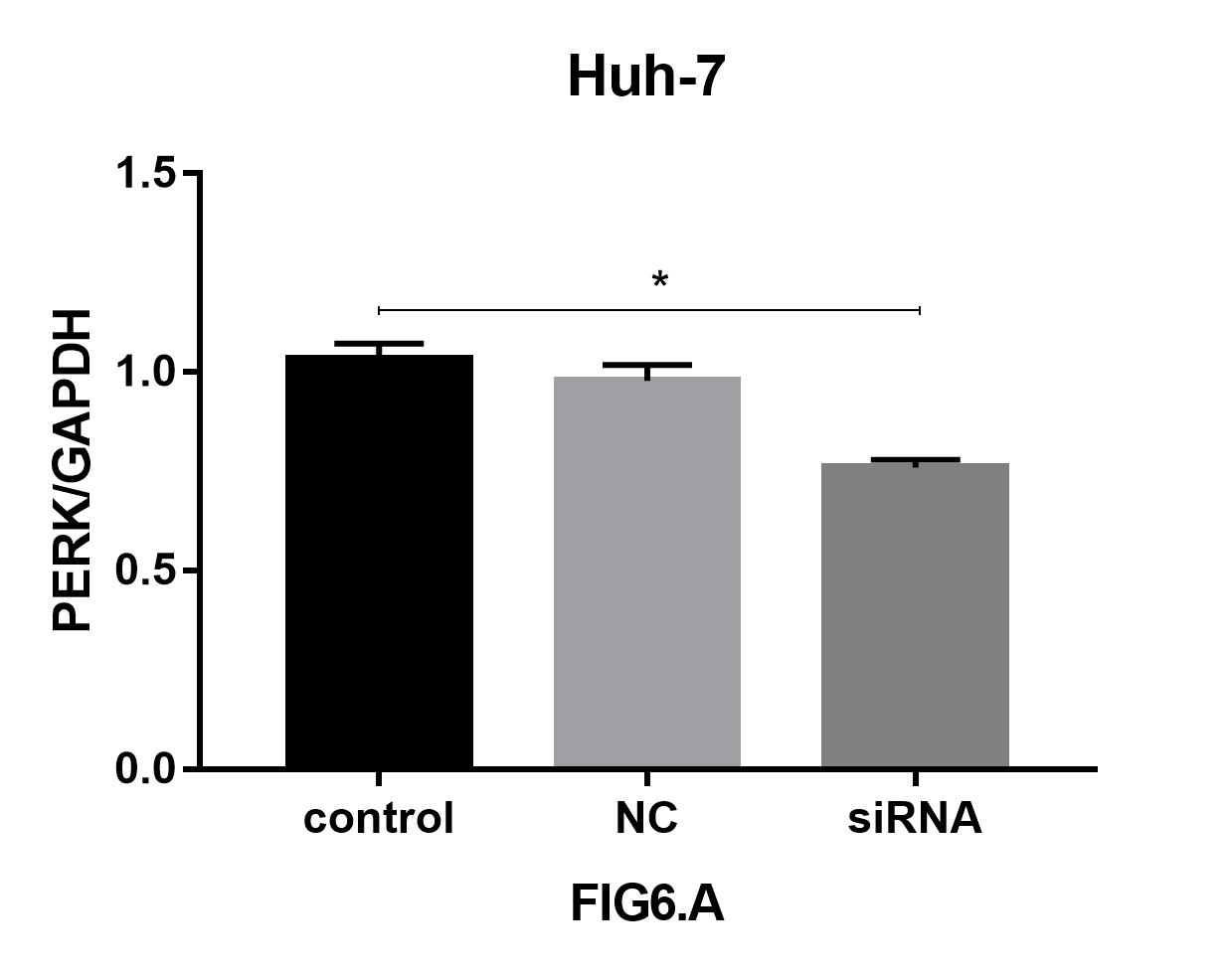


Fig 6A. huh-7-PERK


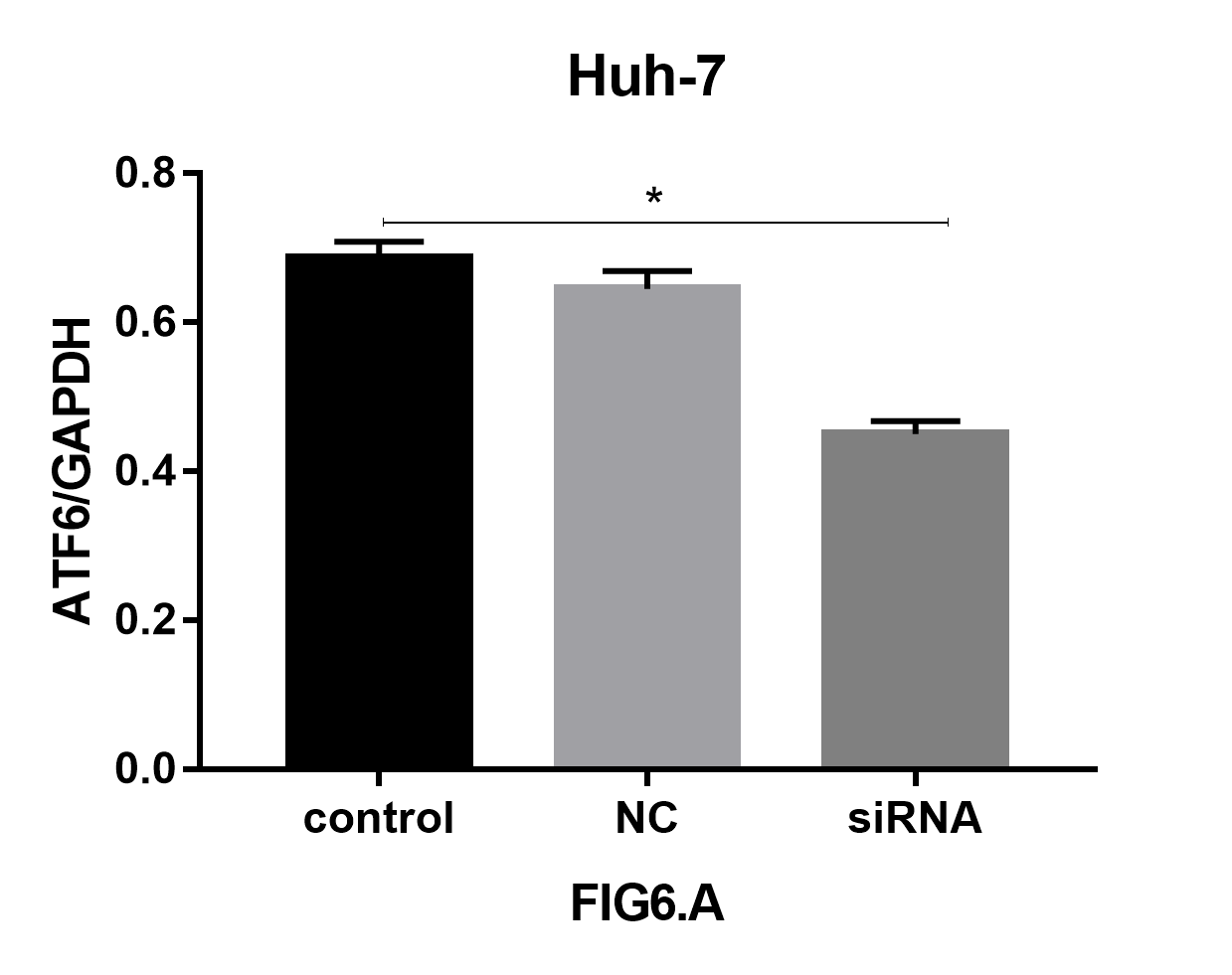


Fig 6A. huh-7-ATF6


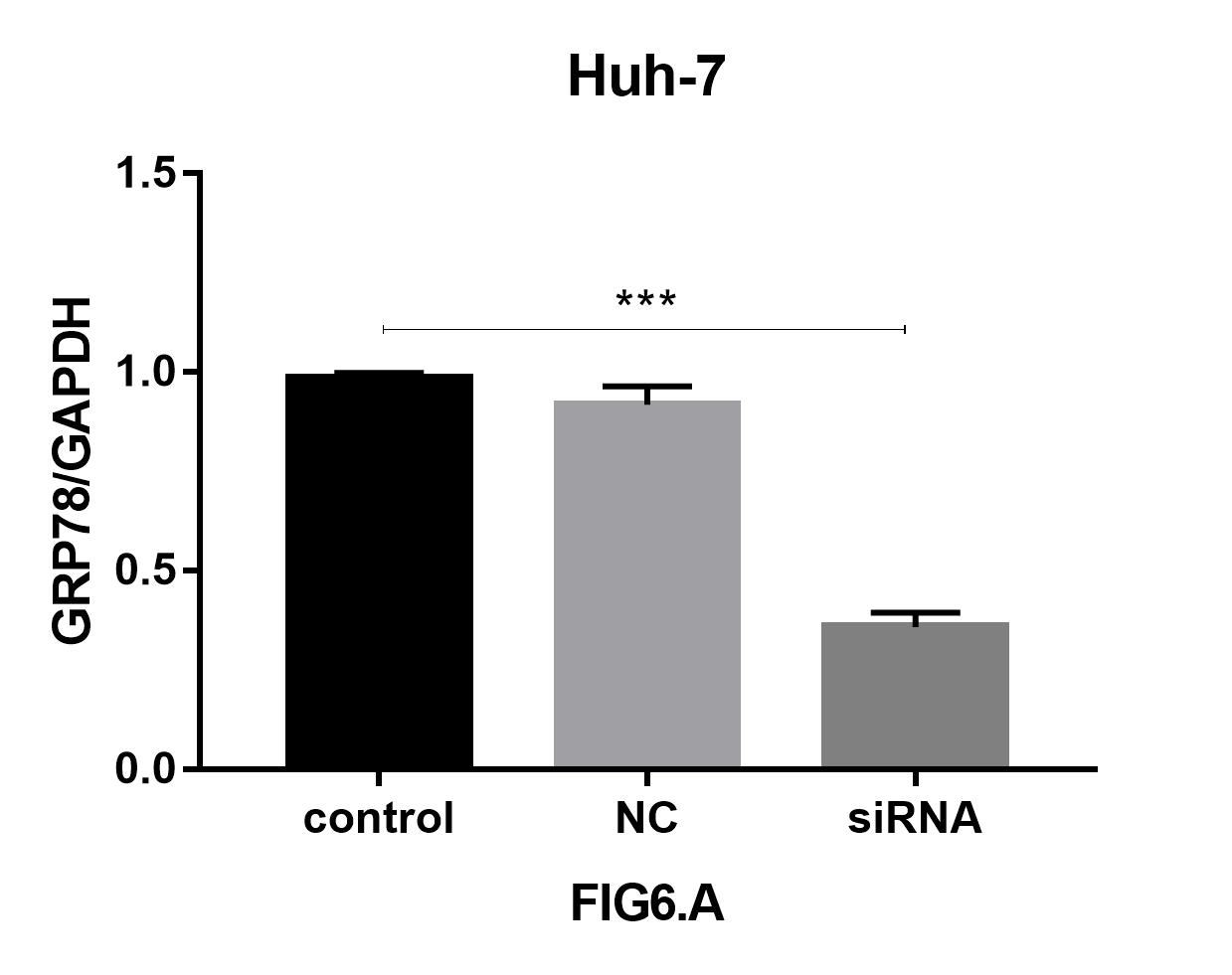


Fig 6A. huh-7-GRP78


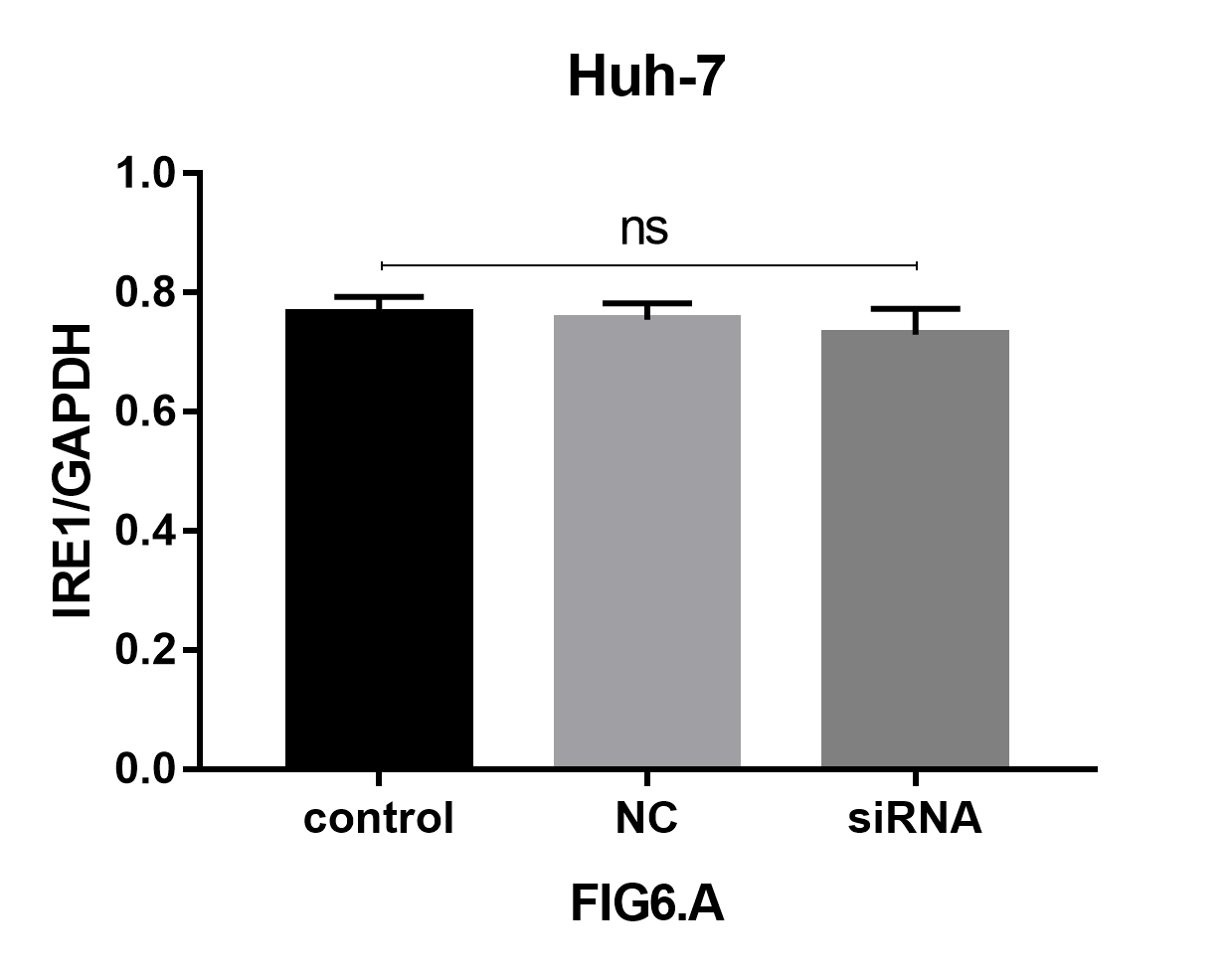


Fig 6A. huh-7-IRE-1


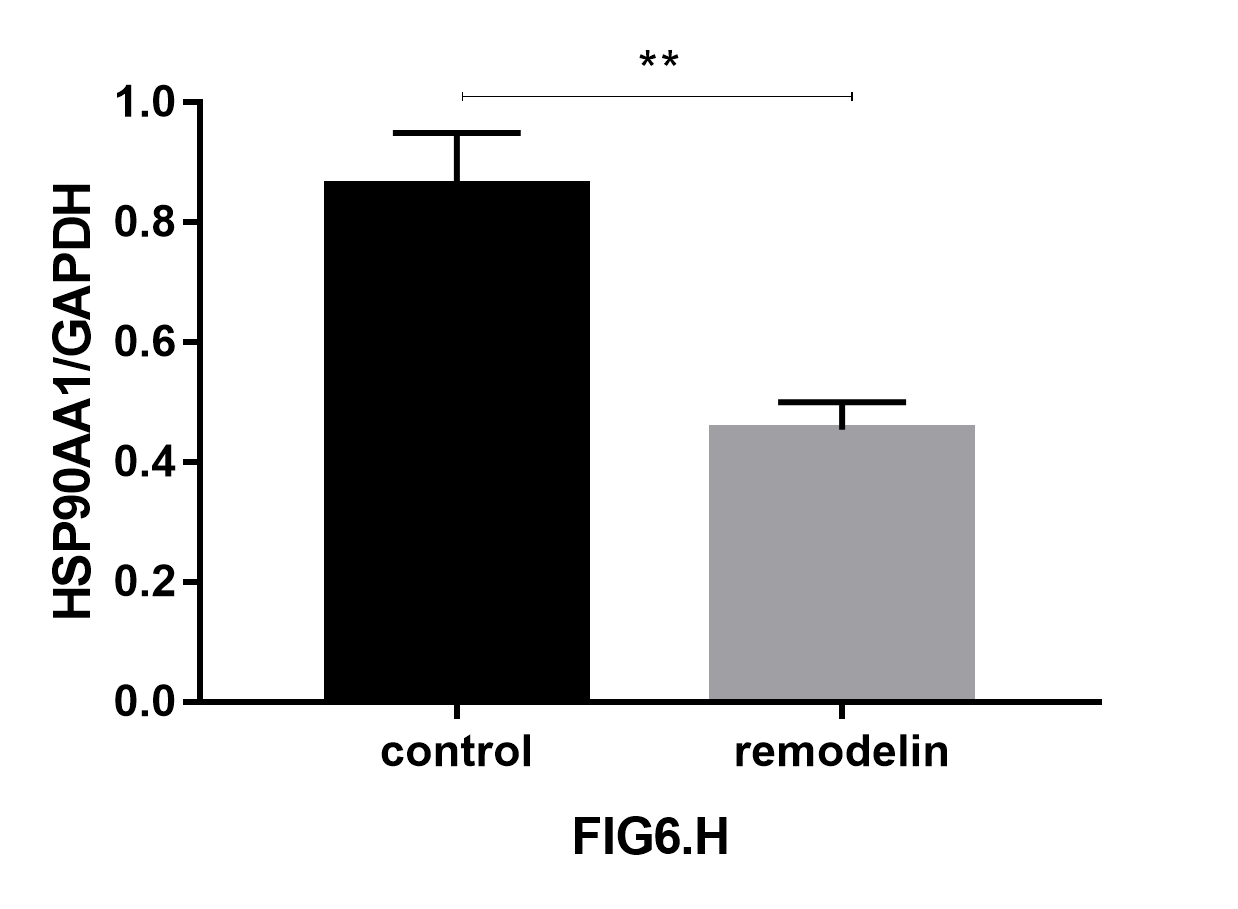


Fig 6H. Mouse tumor tissue-HSP90AA1


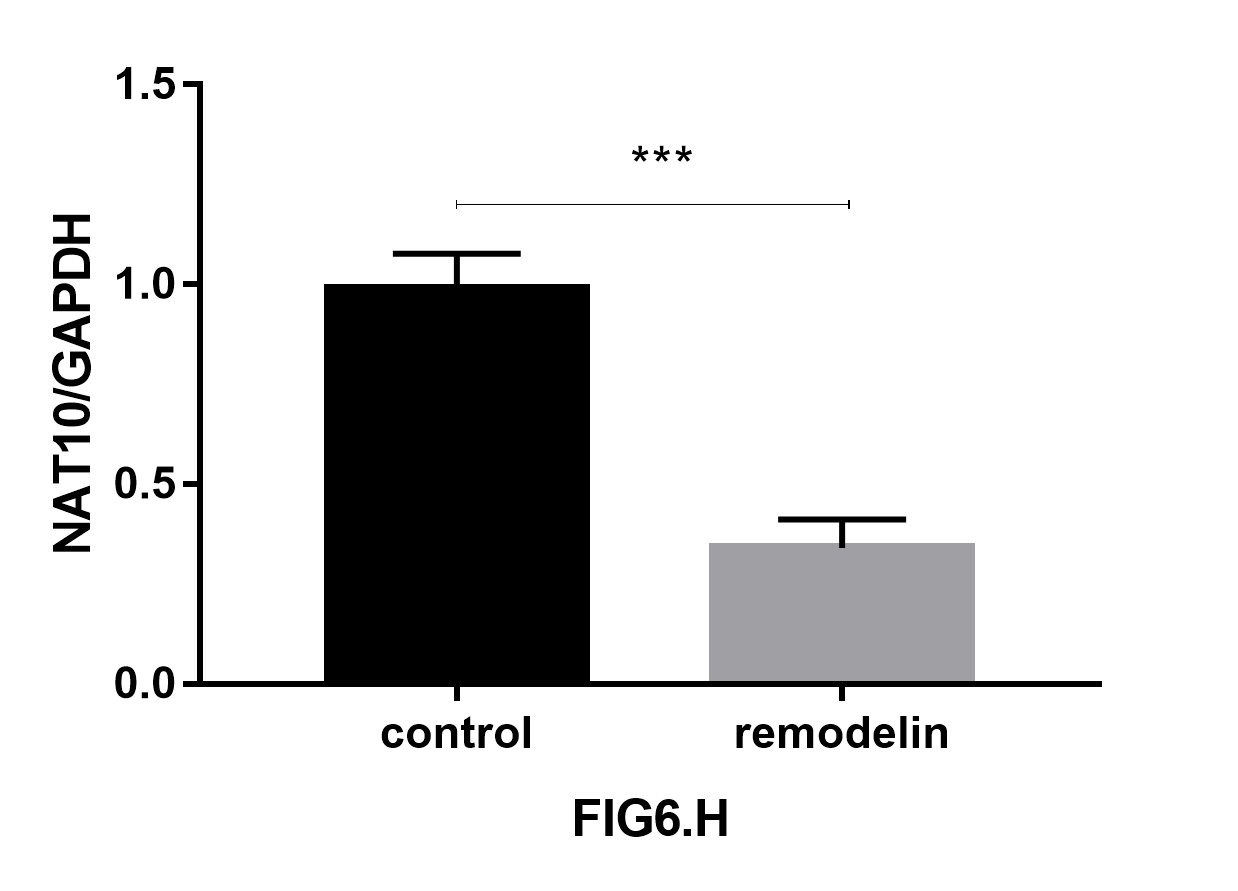


Fig 6H. Mouse tumor tissue-NAT10


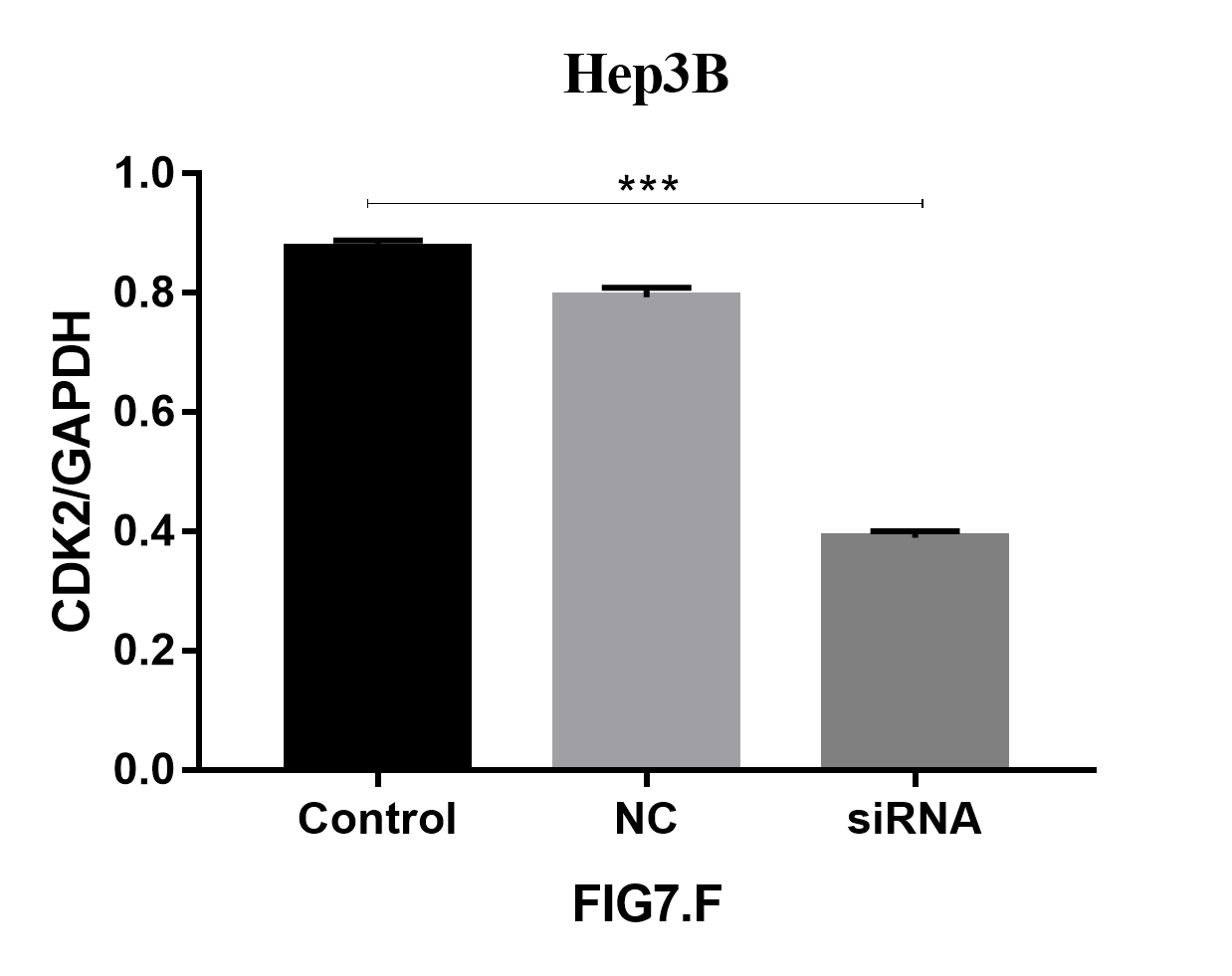


Fig 7F. hep3b-CDK2


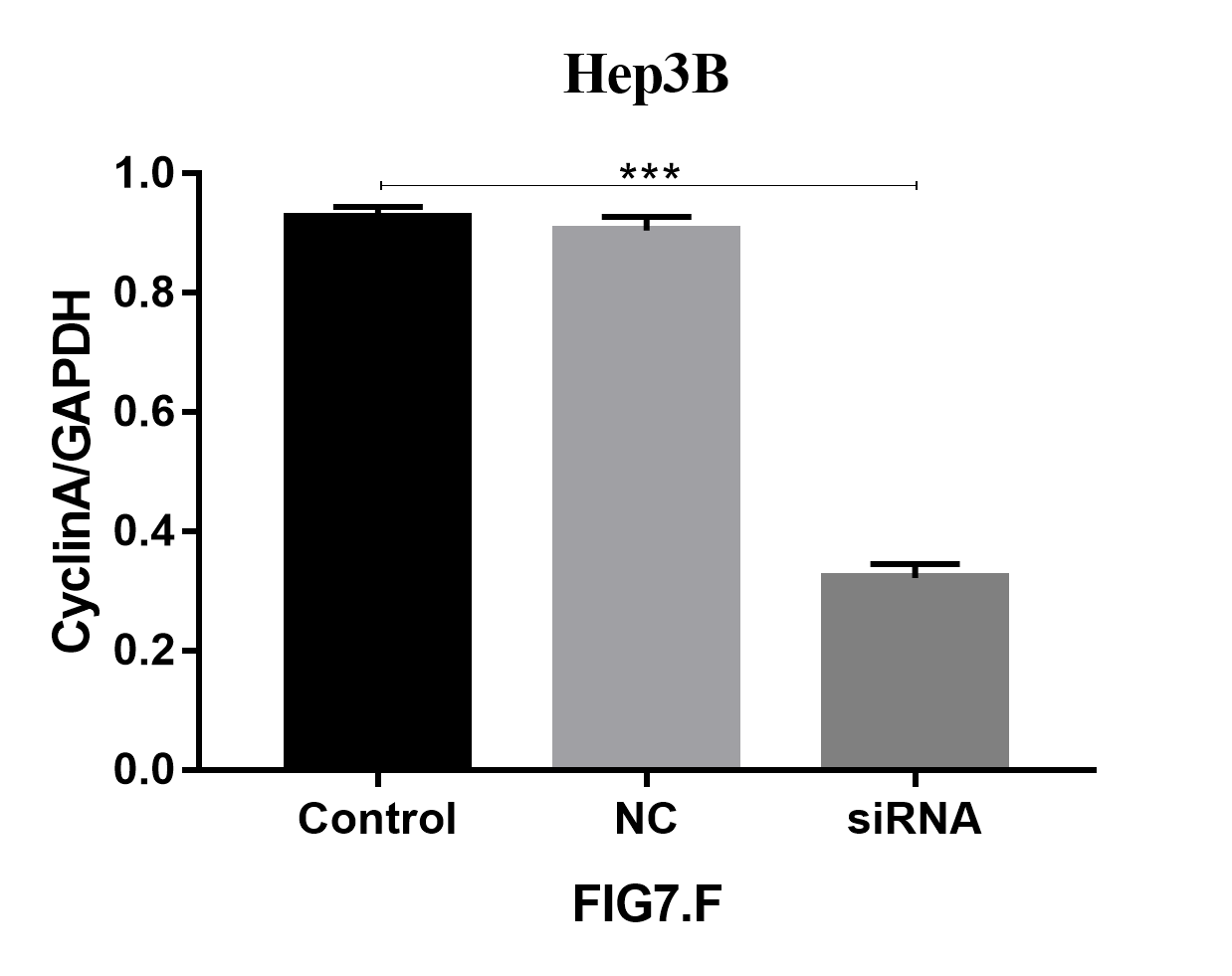


Fig 7F. hep3b-CyclinA


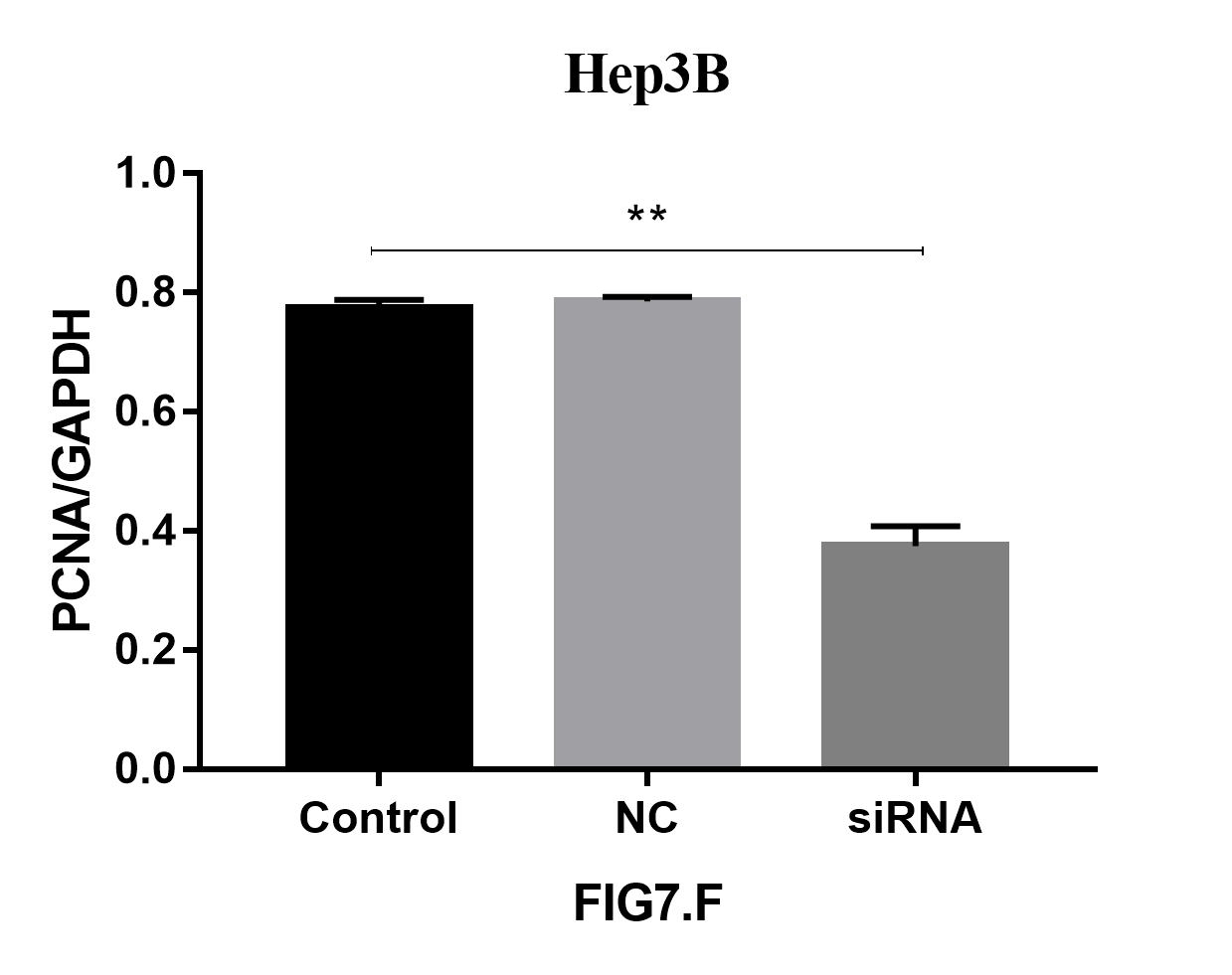


Fig 7F. hep3b-PCNA


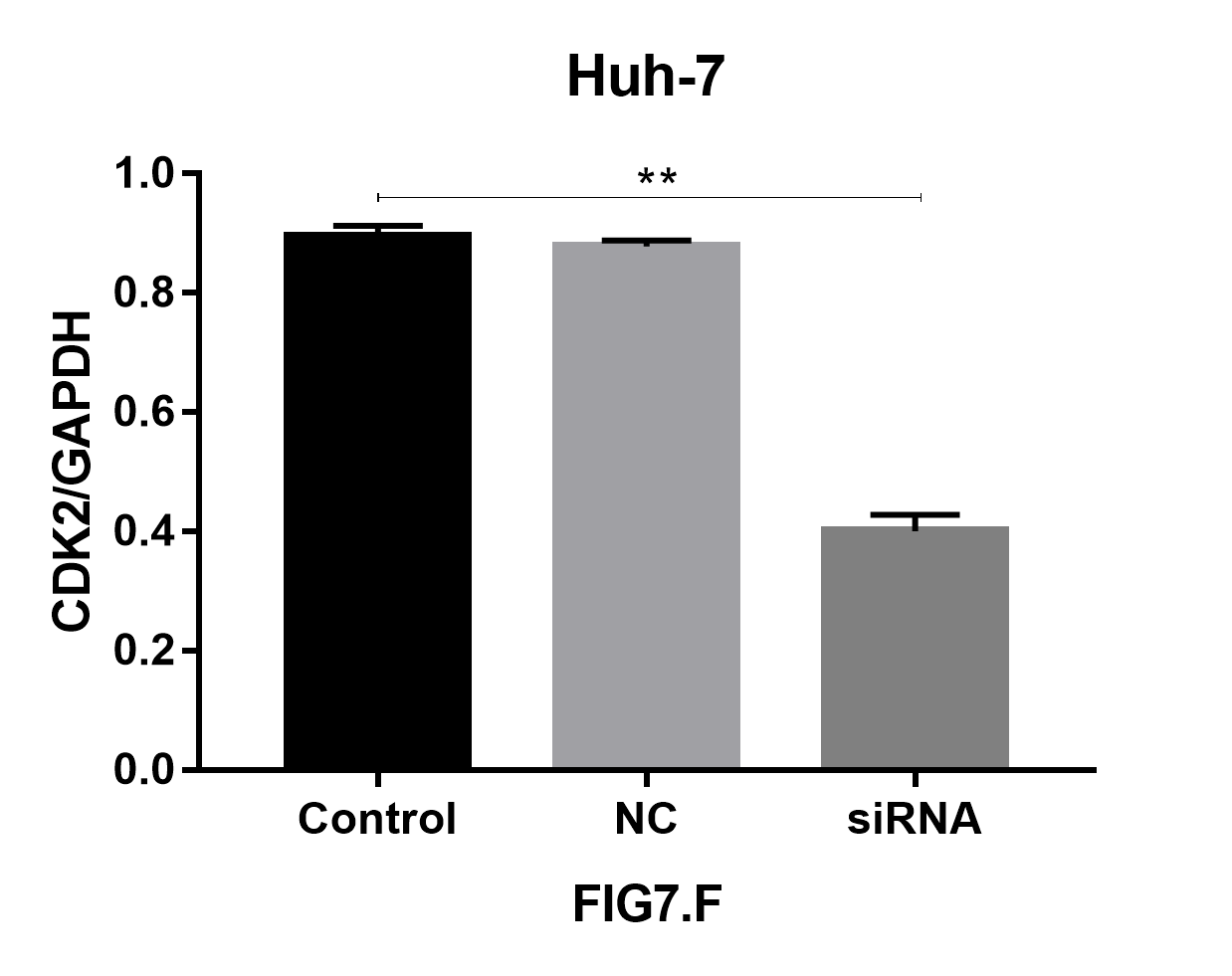


Fig 7F. hep3b-PCNA


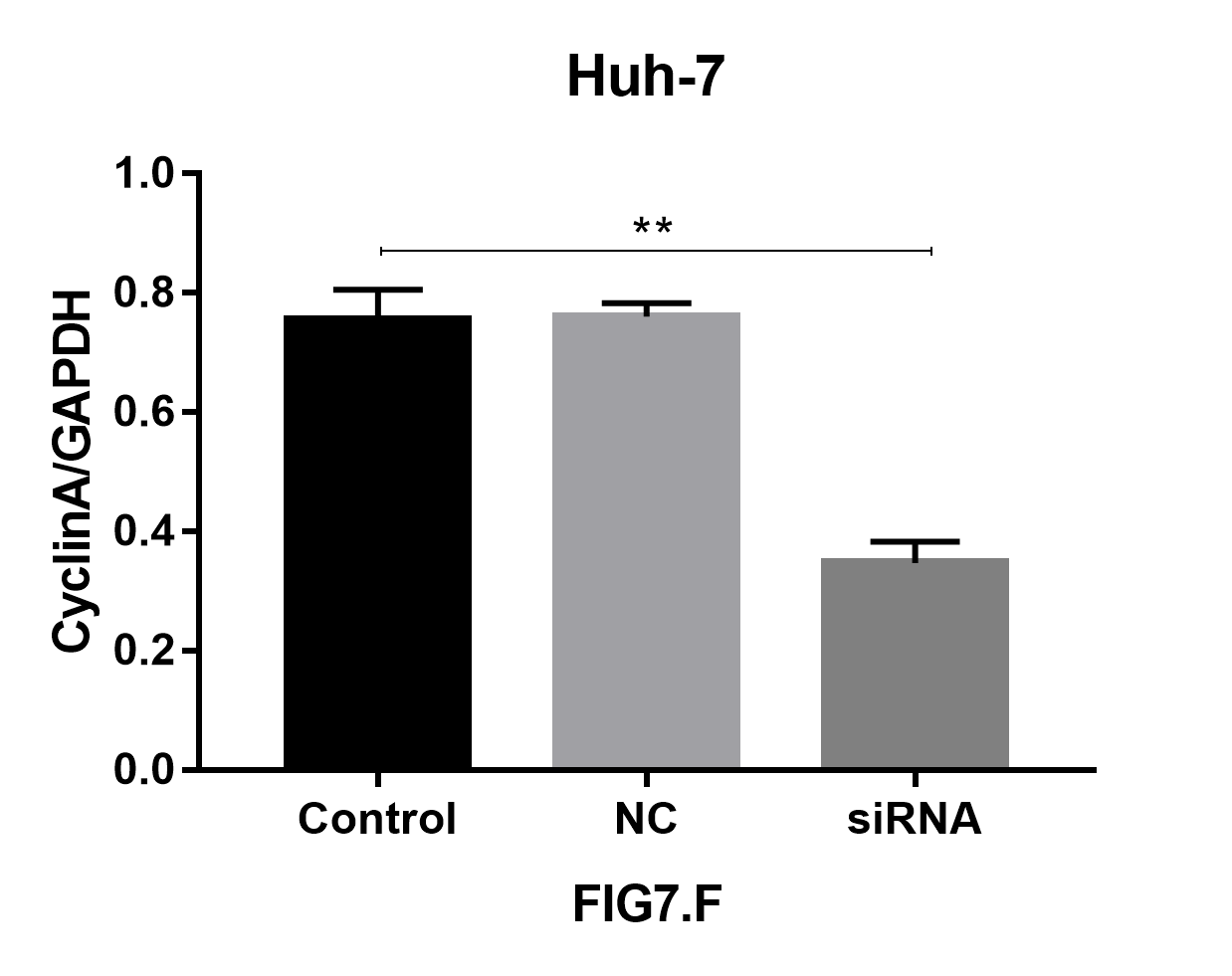


Fig 7F. huh-7-CyclinA


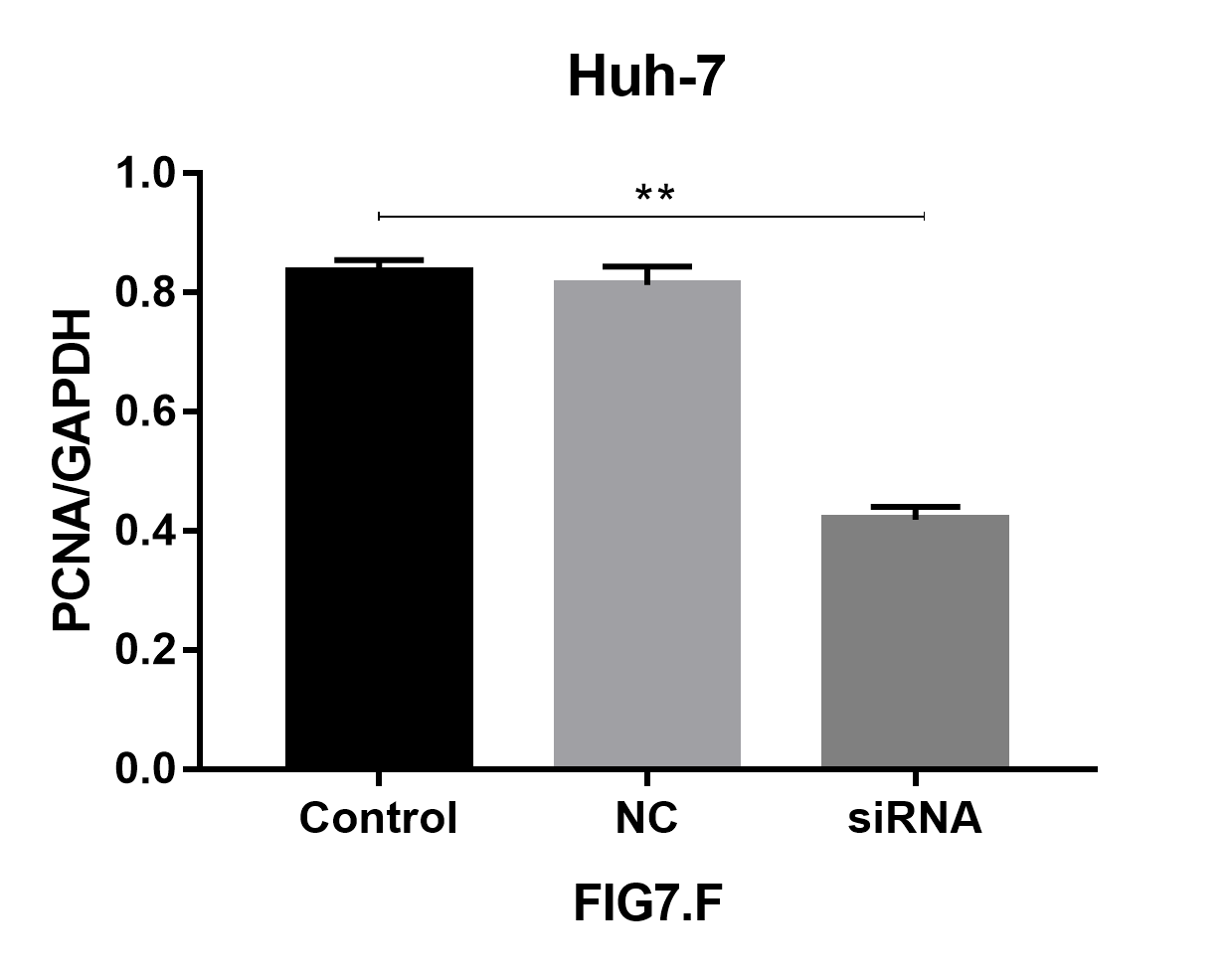


Fig 7F. huh-7-PCNA
