# Supplementary material for: Role of NAT10-mediated ac4C-modified HSP90AA1 RNA acetylation in ER stress-mediated metastasis and lenvatinib resistance in hepatocellular carcinoma
Source: Cell Death Discov. 2023 Feb 10;9:56. doi: 10.1038/s41420-023-01355-8 (PMC9918514; doi:10.1038/s41420-023-01355-8)
Supplement: Supplementary file 6 — Quantified results and statistical analysis of immunohistochemical assay [file 41420_2023_1355_MOESM6_ESM.docx]

**Quantified results and statistical analysis of immunohistochemical assay**

**Table of contents**

**Figure of the results of ATF6 immunohistochemical assay**

**Figure of the results of GRP78 immunohistochemical assay**

**Figure of the results of IRE-1 immunohistochemical assay**

**Figure of the results of NAT10 immunohistochemical assay**

**Figure of the results of PERK immunohistochemical assay**


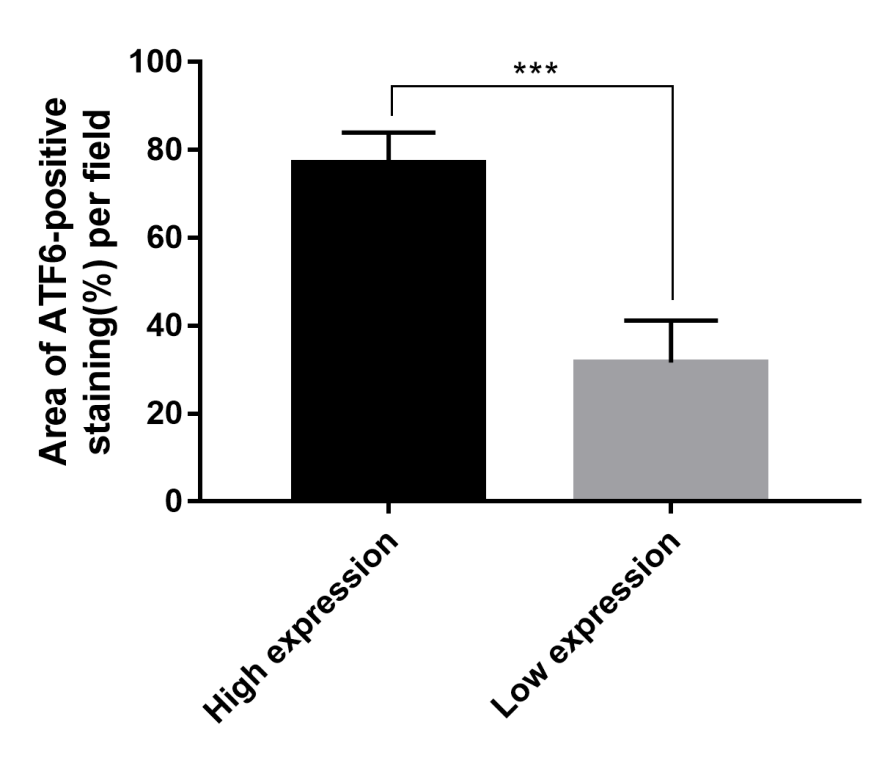


Figure of the results of ATF6 immunohistochemical assay


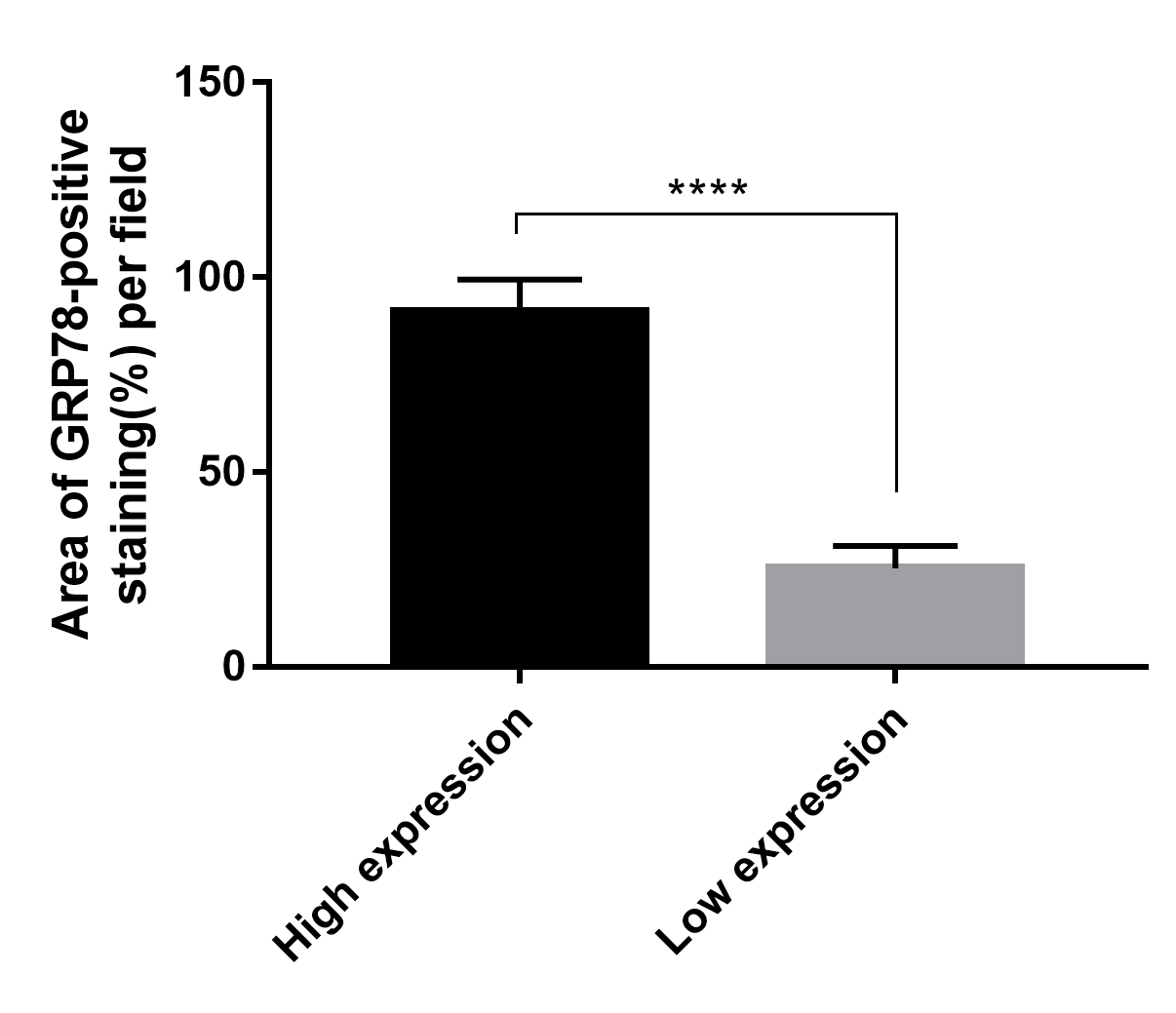


Figure of the results of GRP78 immunohistochemical assay


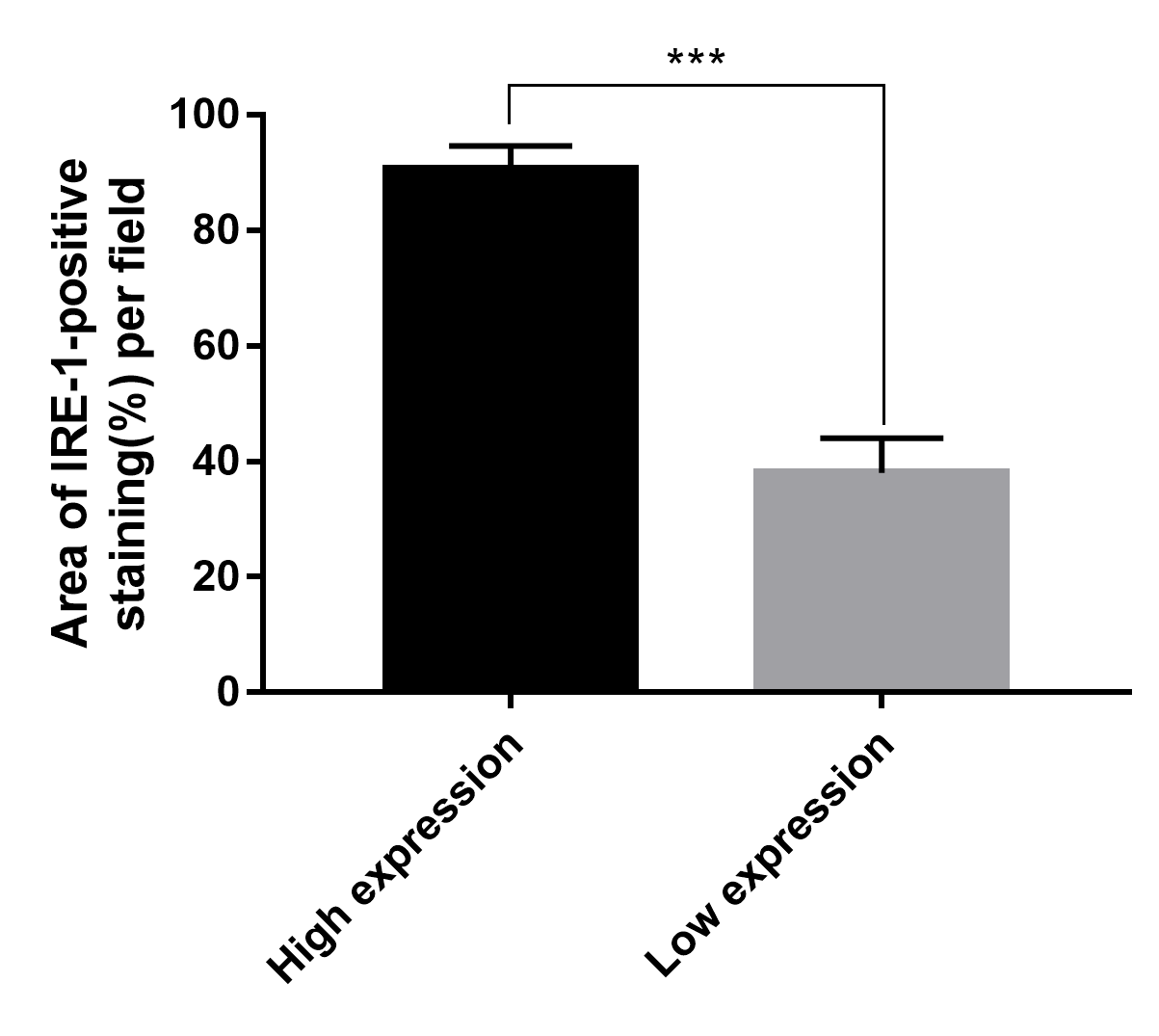


Figure of the results of IRE-1 immunohistochemical assay


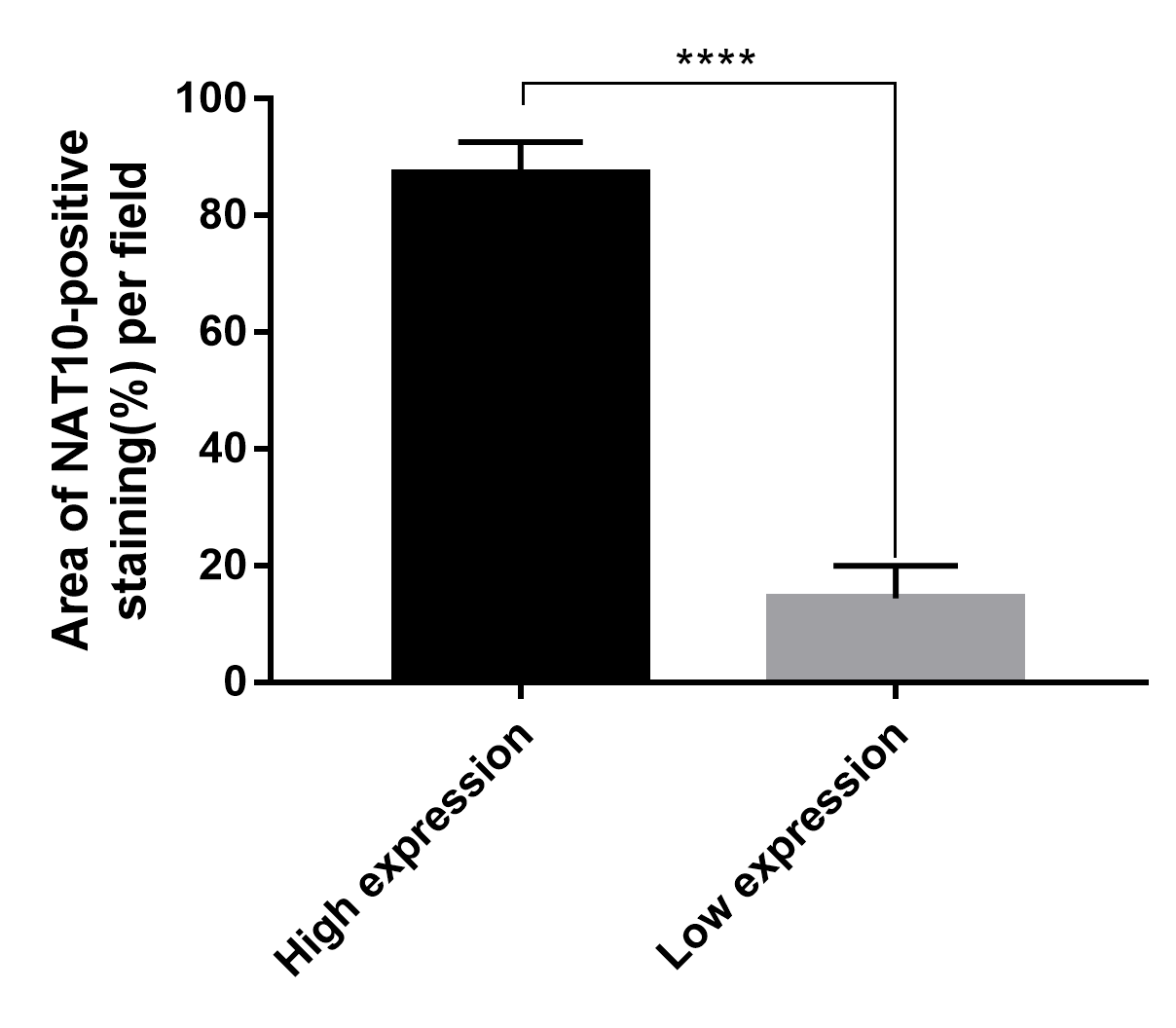


Figure of the results of NAT10 immunohistochemical assay


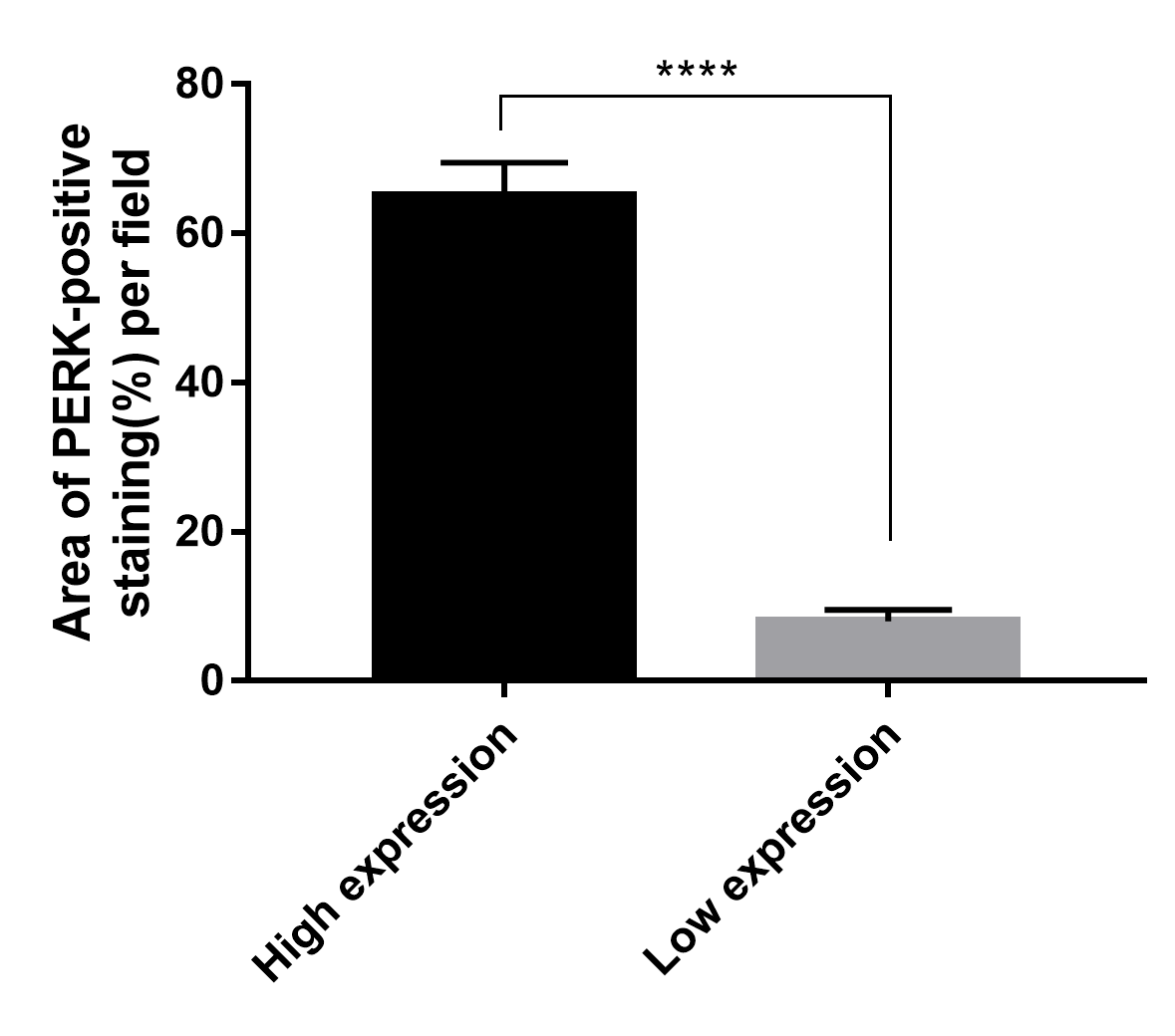


Figure of the results of PERK immunohistochemical assay
