# Supplementary material for: Role of NAT10-mediated ac4C-modified HSP90AA1 RNA acetylation in ER stress-mediated metastasis and lenvatinib resistance in hepatocellular carcinoma
Source: Cell Death Discov. 2023 Feb 10;9:56. doi: 10.1038/s41420-023-01355-8 (PMC9918514; doi:10.1038/s41420-023-01355-8)
Supplement: Supplementary file 7 — Original Data File [file 41420_2023_1355_MOESM7_ESM.docx]

**Original figures of WB**

**Table of contents**

**Figure 1E**

**Figure 2E**

**Figure 2F**

**Figure 2G**

**Figure 3F**

**Figure 4D**

**Figure 5L**

**Figure 5M**

**Figure 6A**

**Figure 6H**

**Figure 7F**

**Figure1E**


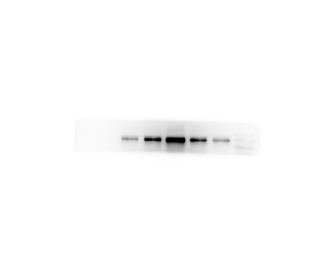


FIG1E-NAT10


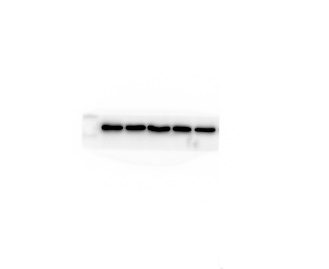


FIG1E-GAPDH

**Figure2E**


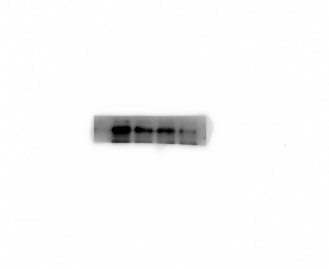


FIG2E-HUH7-NAT10


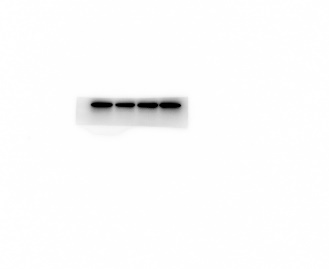


FIG2E-HUH7-GAPDH


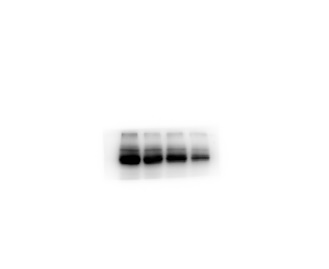


FIG2E-HEP3B-NAT10


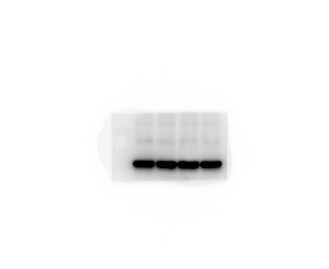


FIG2E-HEP3B-GAPDH

**Figure2F**


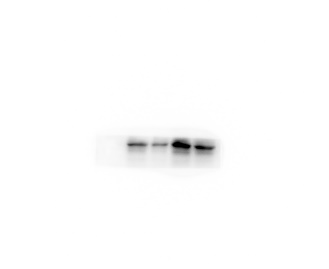


FIG2F-HUH7-GRP78


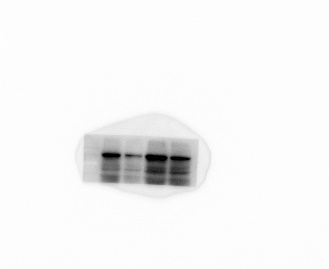


FIG2F-HUH7-ATF6


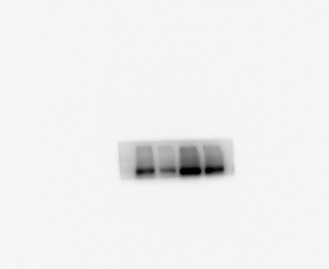


FIG2F-HUH7-IRE-1


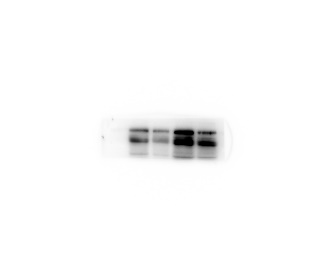


FIG2F-HUH7-PERK


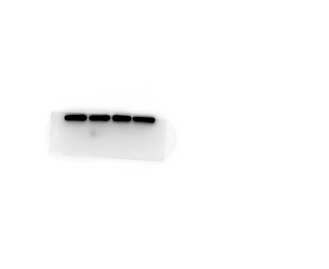


FIG2F-HUH7-GAPDH

**Figure2G**


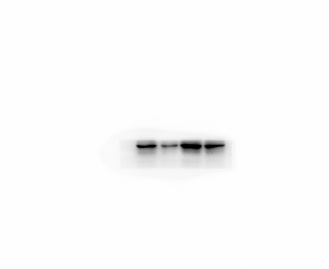


FIG2G-HUH7-GRP78


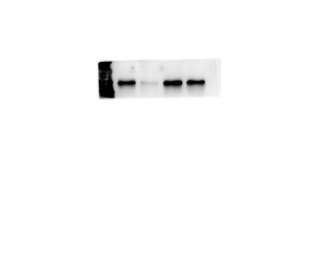


FIG2G-HUH7-ATF6


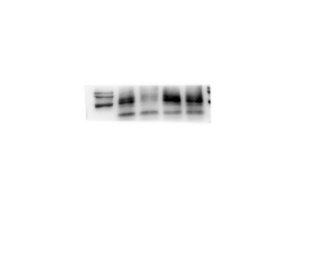


FIG2G-HUH7-IRE-1


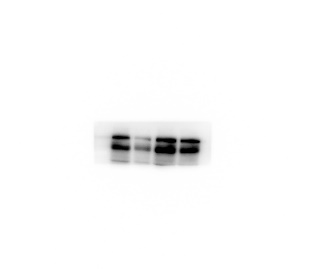


FIG2G-HUH7-PERK


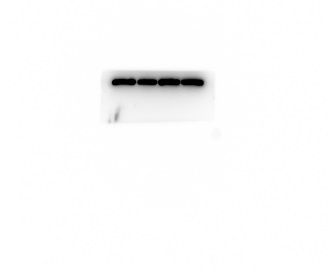


FIG2G-HUH7-GAPDH


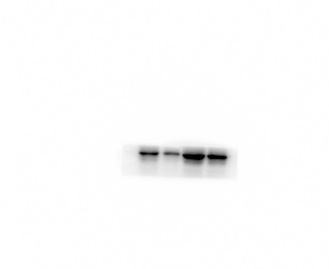


FIG2G-HEP3B-GRP78


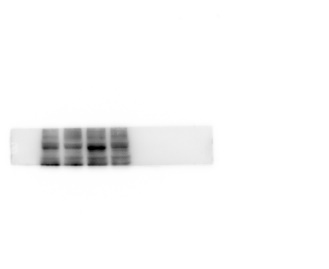


FIG2G-HEP3B-ATF6


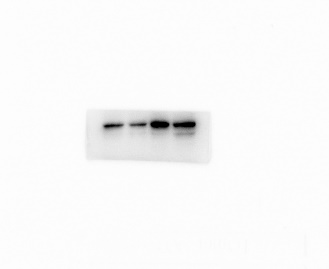


FIG2G-HEP3B-IRE-1


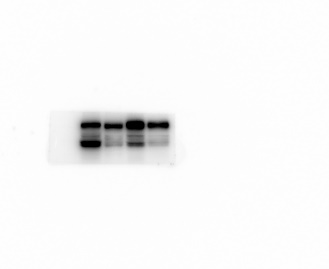


FIG2G-HEP3B-PERK


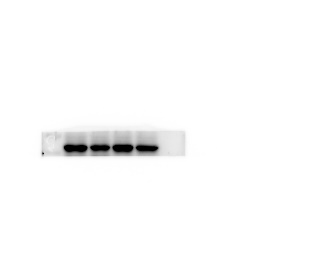


FIG2G-HEP3B-GAPDH

**Figure3F**


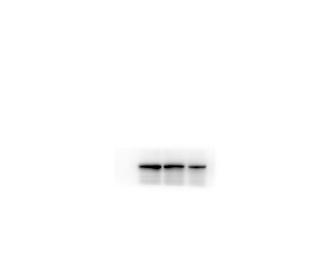


FIG3F-HUH7- CDK2


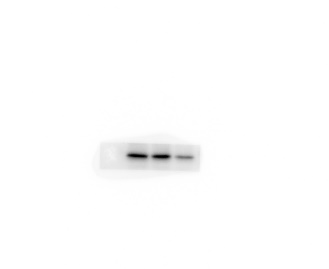


FIG3F-HUH7- CyclinA


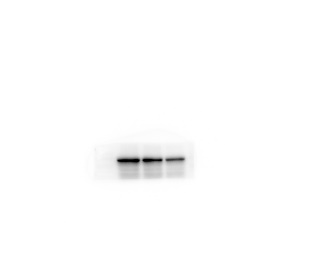


FIG3F-HUH7- PCNA


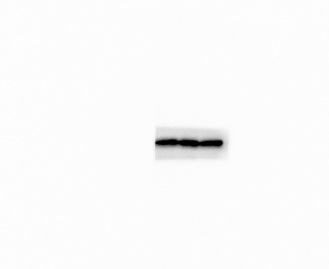


FIG3F-HUH7- GAPDH


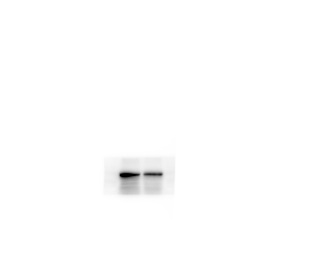


FIG3F-HUH7- CDK2


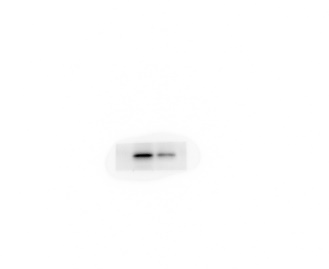


FIG3F-HUH7- CyclinA


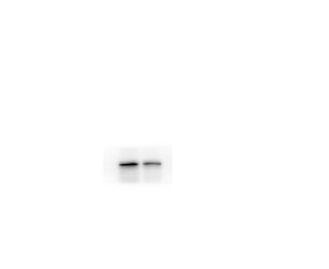


FIG3F-HUH7- PCNA


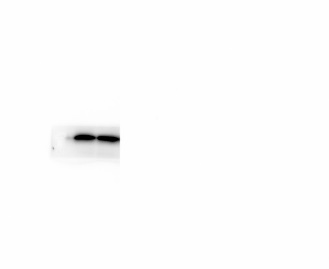


FIG3F-HUH7- GAPDH


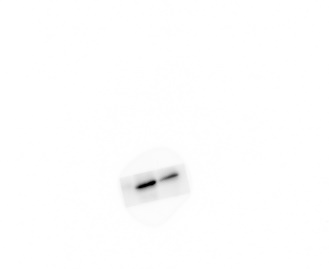


FIG3F-HEP3B- CDK2


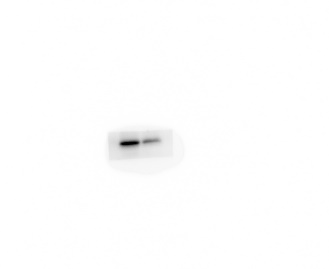


FIG3F-HEP3B- CyclinA


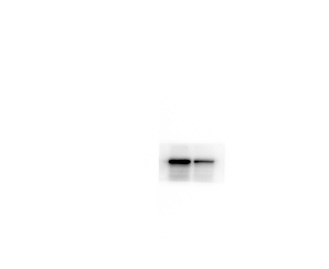


FIG3F-HEP3B- PCNA


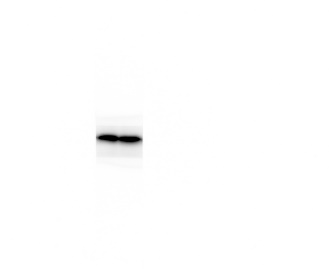


FIG3F-HEP3B- GAPDH

**Figure4D**


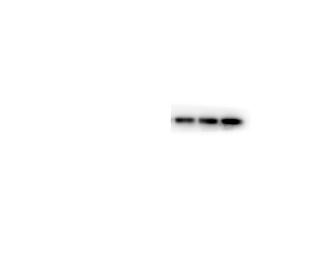


FIG4D-HUH7- Bax


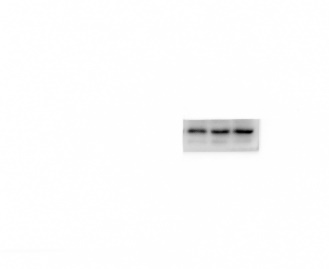


FIG4D-HUH7-Bak


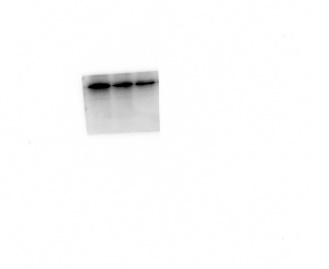


FIG4D-HUH7- Bcl-2


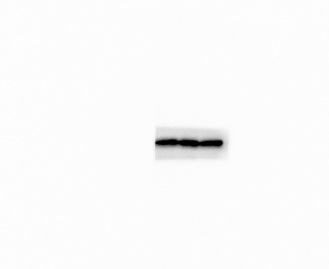


FIG4D-HUH7- GAPDH

**Figure4D**


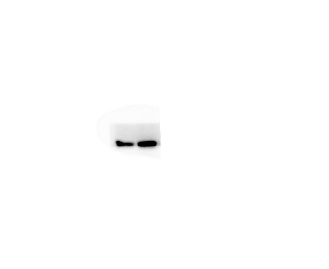


FIG4D-HUH7-Bax


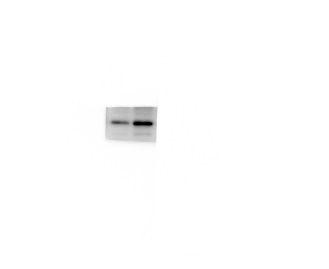


FIG4D-HUH7-Bak


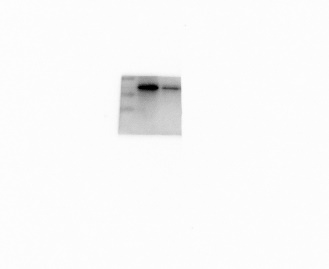


FIG4D-HUH7- Bcl-2


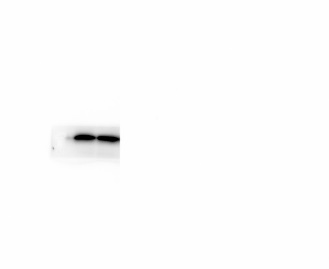


FIG4D-HUH7- GAPDH


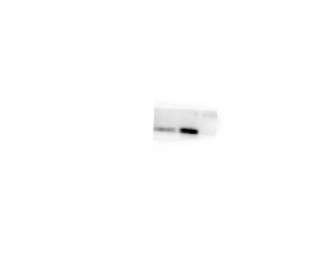


FIG4D-HEP3B-Bax


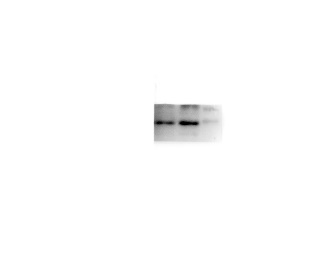


FIG4D-HEP3B-Bak


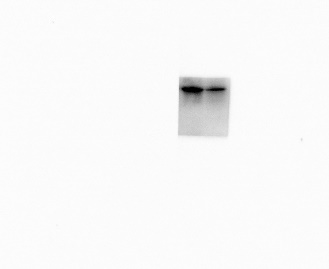


FIG4D-HEP3B- Bcl-2


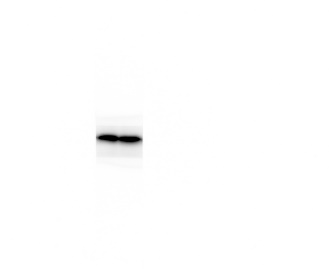


FIG4D-HEP3B- GAPDH

**Figure5L**


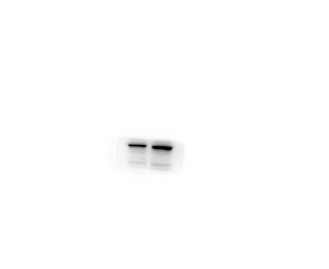


FIG5L-HUH7-HSP90AA1


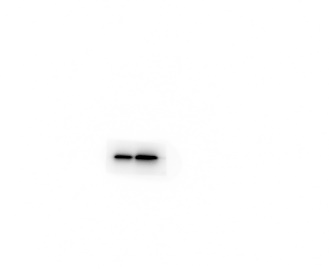


FIG5L-HUH7- NAT10


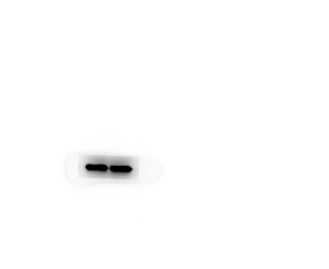


FIG5L-HUH7- GAPDH


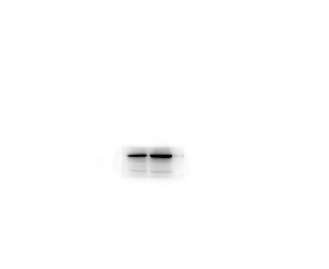


FIG5L-HEP3B-HSP90AA1


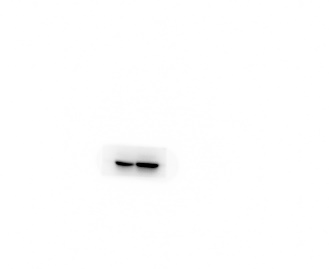


FIG5L-HEP3B- NAT10


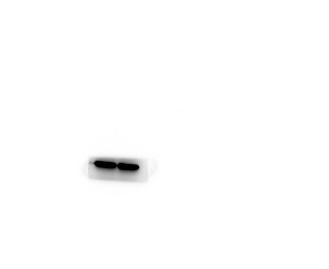


FIG5L-HEP3B-GAPDH

**Figure5M**


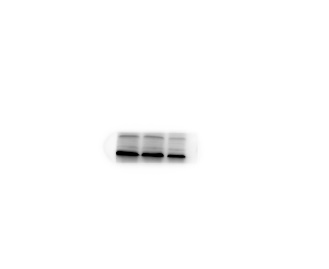


FIG5M-HUH7- HSP90AA1


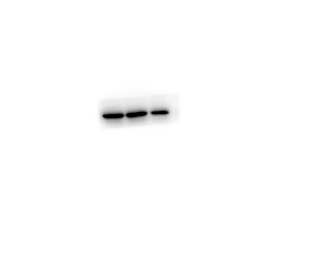


FIG5M-HUH7- NAT10


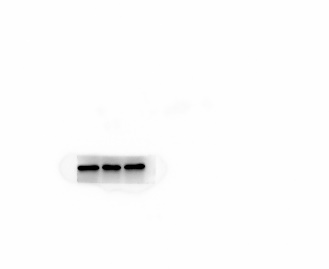


FIG5M-HUH7- GAPDH


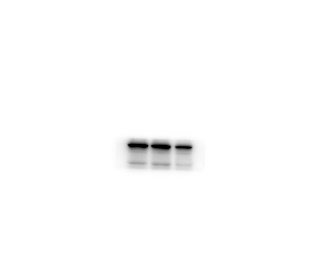


FIG5M-HEP3B-HSP90AA1


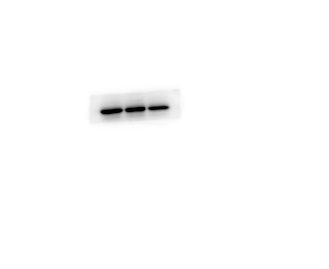


FIG5M-HEP3B- NAT10


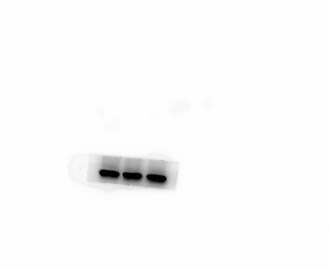


FIG5M-HEP3B- GAPDH

**Figure6A**


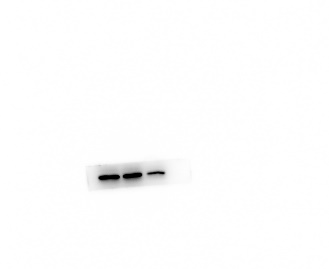


FIG6A-HUH7- HSP90AA1


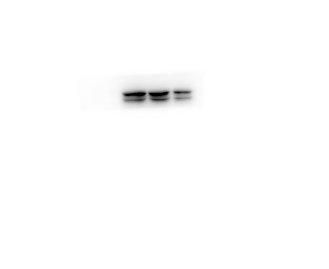


FIG6A-HUH7- GRP78


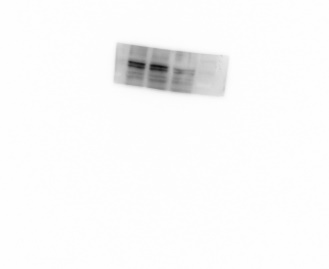


FIG6A-HUH7- ATF6


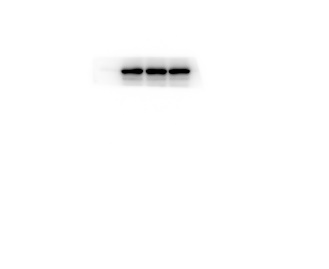


FIG6A-HUH7- IRE-1


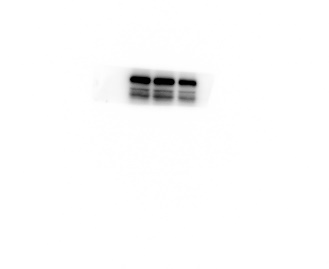


FIG6A-HUH7- PERK


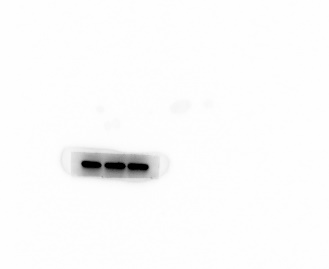


FIG6A-HUH7- GAPDH


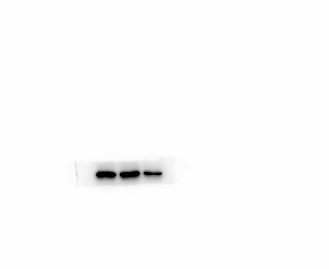


FIG6A-HEP3B- HSP90AA1


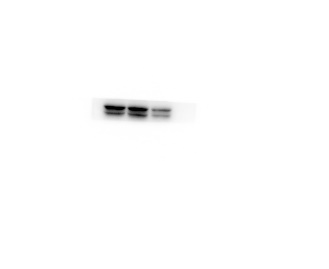


FIG6A-HEP3B- GRP78


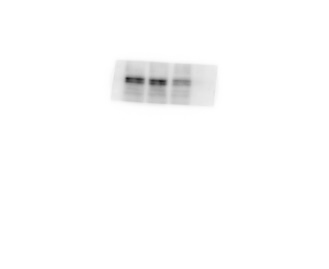


FIG6A-HEP3B- ATF6


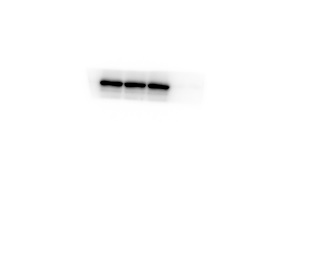


FIG6A-HEP3B- IRE-1


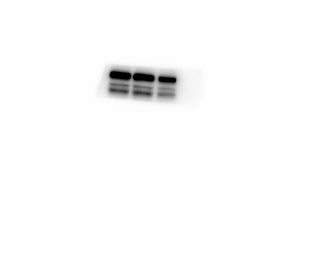


FIG6A-HEP3B- PERK


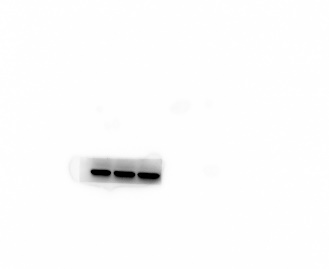


FIG6A-HEP3B- GAPDH

**Figure6H**


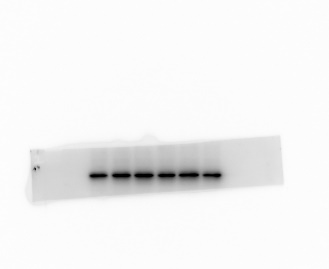


FIG6H-control-NAT10


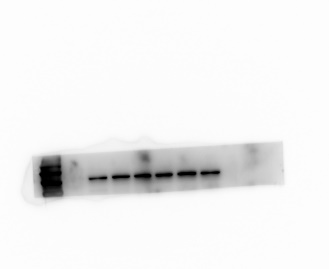


FIG6H-control-HSP90AA1


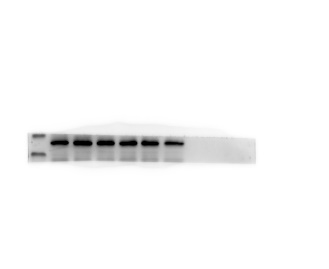


FIG6H-control-GAPDH


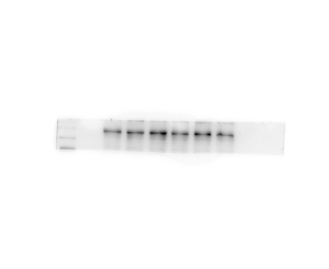


FIG6H-rem-NAT10


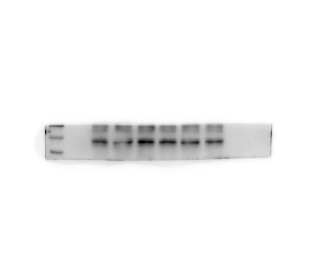


FIG6H-rem-HSP90AA1


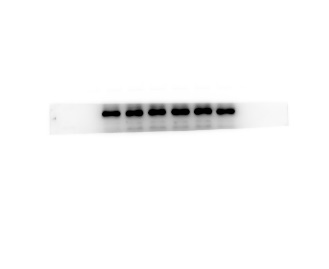


FIG6H-rem-GAPDH

**Figure7F**


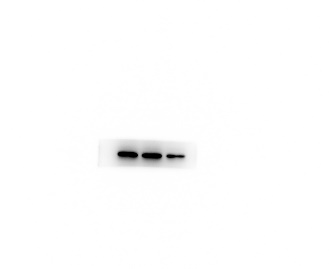


FIG7F-HUH7- CDK2


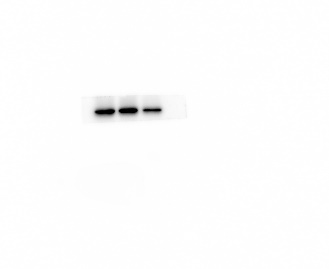


FIG7F-HUH7- CyclinA


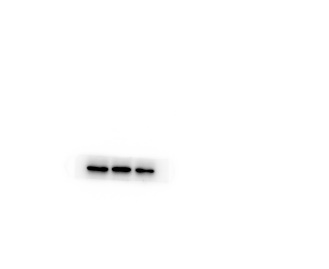


FIG7F-HUH7- PCNA


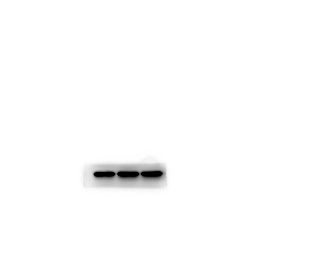


FIG7F-HUH7- GAPDH


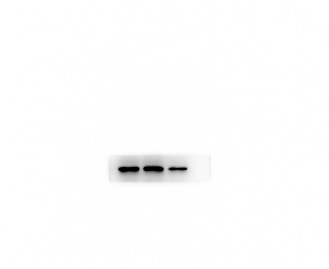


FIG7F-HEP3B- CDK2


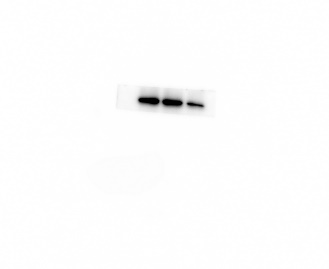


FIG7F-HEP3B- CyclinA


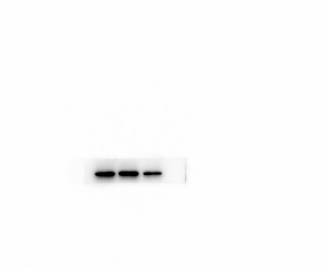


FIG7F-HEP3B- PCNA


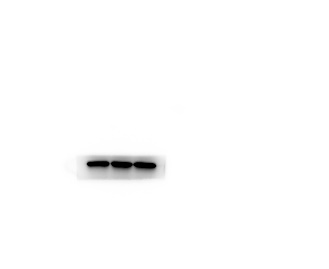


FIG7F-HEP3B- GAPDH
